# Theoretical Insights into ESIPT and GSIPT in Schiff Base Cu(II)-BSSMO Complexes: Substituent Effects Explored via DFT, Molecular Docking, and Dynamics Simulations for Optoelectronic and Biomedical Applications

**DOI:** 10.1021/acsomega.5c06772

**Published:** 2025-11-12

**Authors:** Murugesan Panneerselvam, Anantha Narayanan Sri Gayathri, Singaravel Nathiya, Jarede Da Silva Martins, Iravatham Rama, Frederico W. Tavares, Luciano T. Costa

**Affiliations:** † MolMod-CS, Institute of Chemistry, Fluminense Federal University (UFF), Niterói, RJ CEP: 24020-141, Brazil; ‡ Chemical Engineering Program (PEQ/COPPE), Federal University of Rio de Janeiro (UFRJ), Rio de Janeiro, RJ CEP: 21941-594, Brazil; § PG and Research Department of Chemistry, Seethalakshmi Ramaswami College, Affiliated to Bharathidasan University, Tiruchirappalli, Tamil Nadu 620 002, India; ∥ Chemical and Biochemical Process Engineering, School of Chemistry, Federal University of Rio de Janeiro (UFRJ), Rio de Janeiro, RJ CEP: 21941-594, Brazil

## Abstract

Excited-state intramolecular proton transfer (ESIPT) is a key photophysical mechanism with broad implications for the development of advanced materials for optoelectronics, sensing, and biomedical applications. In this study, the effects of electron-donating (EDG) groups (−NH_2_, −OCH_3_, and −CH_3_) and electron-withdrawing (EWG) groups (−Cl, −Br, −COOH, −CF_3_, −CN, and −NO_2_) on ESIPT and ground-state intramolecular proton transfer (GSIPT) were systematically explored in 10 BSSMO derivatives using a combination of density functional theory (DFT), time-dependent DFT (TD-DFT), transition state analysis, and molecular simulations. The results show that EDGs enhance ESIPT efficiency by increasing electron density around proton donor/acceptor sites, promoting charge delocalization, and lowering proton transfer barriers, as evidenced by bathochromic shifts in absorption and emission spectra. On the other hand, EWGs strengthen hydrogen bonding in the ground state and cause shifts to shorter wavelengths, which make the molecules stiffer and reduces ESIPT. Frontier molecular orbital (FMO) analysis indicates that EDG substitution reduces *E*_g_, improving reactivity and facilitating photoinduced transitions. Further insights from QTAIM and MESP analyses reveal distinct substituent-dependent electronic and bonding characteristics. Biological assessments through molecular docking show that keto tautomers, especially with EDGs, possess stronger binding affinities toward the human histo-aspartic protease (HAP) protein, consistent with improved pharmacokinetic properties predicted via ADMET analysis. Molecular dynamics (MD) simulations further highlight the specific roles of −NH_2_ and −NO_2_ in mediating protein–ligand interactions. Overall, correlation analysis shows that electron-withdrawing substituents (positive σ) enhance electronic stability by increasing the energy gap, ionization potential (IP), and electron affinity (EA), while electron-donating substituents (negative σ) promote charge delocalization and transport. This study further establishes a clear structure–property relationship, highlighting that strategic substitution can precisely tune ESIPT/GSIPT behavior, optoelectronic properties, and biological activity, making Cu-BSSMO derivatives promising multifunctional candidates for advanced applications.

## Graphical abstract

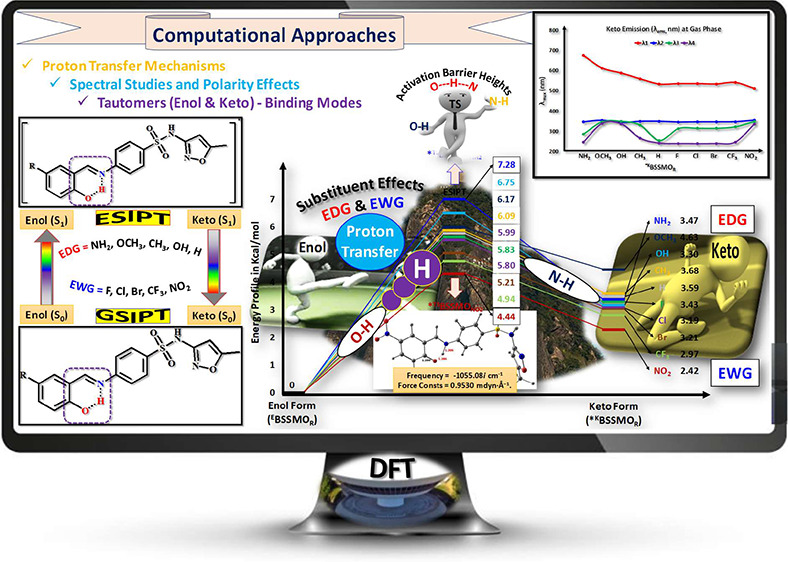

## 1 Introduction

Schiff bases have garnered significant interest due to their facile synthesis and exceptional versatility in coordination chemistry.^1^ Although Hugo Schiff extensively studied the reaction between primary amines and carbonyl compounds in 1864, the term “Schiff base” was later introduced by the scientific community in recognition of his contributions to the field. These compounds are characterized by the −C=N– group, a carbon–nitrogen double bond, and are synthesized by combining various alkyl or aryl substituents, giving rise to a diverse array of Schiff bases. Even more than a decade after their discovery, these bases play a pivotal role in coordination chemistry as essential ligands. The azomethine group in particular remains a key component in revealing the potential of Schiff bases to form complexes with transition metal ions, thereby enhancing their applications.^2–5^ Schiff bases are renowned for their capacity to form stable and robust complexes with a diverse range of metal ions, and such complexes are typically synthesized through the reaction of Schiff bases with appropriate metal precursors under specific conditions.^6–8^ They are commonly employed as chelating ligands to enhance the stability and reactivity of metal complexes.^9,10^ Additionally, these compounds serve various functions, including acting as catalysts in chemical reactions,^11,12^ as dyes in coloring processes,^13,14^ as initiators in polymerization reactions, and as luminescent materials in light-emitting applications.^15,16^ In biological research, Schiff bases and their metal complexes have been explored for a variety of therapeutic applications.^17,18^ These compounds have demonstrated considerable potential as antibacterial,^19,20^ antifungal,^21^ antitumor,^22^ and antiviral agents^23,24^ and have also been investigated for their effectiveness as insecticides.^25^ Their diverse biological activities highlight their potential for developing new treatments and pest control strategies. The discovery of cisplatin in the 1970s and auranofin in the 1990s reignited interest in metal complexes as promising drug candidates, continuing to captivate researchers in drug design and discovery.^26^

Metal complexes of Schiff bases possess flexible structures, enabling them to adapt their conformation to the coordination geometry of the metal ion. These structural adjustments can alter the electronic and magnetic properties of the complexes, enhancing their utility in a variety of applications.^27^ The distinctive properties of the metal ions are significantly affected by both intra- and intermolecular interactions, such as hydrogen bonding, π···π stacking, coordinate bonding, and electron transfer interactions.^28^ Furthermore, the formation of metal complexes improves their capacity to develop supramolecular structures through hydrogen bonding and other intermolecular forces.^29^ Among various metal ions, copper (Cu) complexes are of particular interest due to Cu’s biological relevance and redox activity. As the third most abundant trace metal in the human body, copper contributes to oxidative balance by participating in redox reactions. It exhibits both antioxidant properties—by scavenging reactive oxygen species (ROS) like peroxides and free radicals—and prooxidant behavior, by catalyzing the oxidation of other molecules.^30^ To combat the harmful effects of prooxidants, biological systems have developed strategies to counteract them, producing enzymes and nonenzymatic agents like superoxide dismutase (SOD) and catalase, which act as ROS scavengers.^31^ Due to the ability of Cu to inhibit free radical production, it is a promising candidate for cancer therapy with minimal side effects. Moreover, it is a vital micronutrient, playing a pivotal role in numerous enzymatic processes and being essential for the formation of connective tissues, the insulation of nerve fibers, and the strengthening of bones.^32–34^ Computational approaches such as density functional theory (DFT) provide detailed insights into the electronic structures, coordination geometries, and reaction mechanisms of Schiff base-metal complexes.^35^

This study explores key photophysical processes, ground and excited-state intramolecular proton transfer (GSIPT and ESIPT), to advance the design of novel optoelectronic materials.^36–40^ This research aims to enhance our understanding of these mechanisms and contribute to developing novel materials with promising optoelectronic applications.^41–46^ Based on our previous studies,^47^ we design ESIPT-active metal complexes by incorporating tailored electron donors and acceptors. Substituent effects play a vital role in tuning the thermodynamics, kinetics, and directionality of proton transfer, enabling the design of materials with targeted photo reactivity, such as photo switches and responsive systems.^48–52^ To achieve this, we employ detailed quantum chemical calculations with the following specific objectives: to systematically investigate the impact of electron-donating (−NH_2_, −OCH_3_, and −CH_3_) and electron-withdrawing (−Cl, −Br, −COOH, −CF_3_, −CN, and −NO_2_) groups on GSIPT and ESIPT processes in BSSMO derivatives using DFT, TD-DFT, transition state analysis, and molecular simulations; to elucidate structure–property relationships by connecting substituent effects to variations in electronic distribution, molecular geometry, hydrogen bonding, vibrational features, and charge delocalization; and to analyze optical absorption and emission behaviors, assess reactivity and stability trends through frontier molecular orbitals (FMOs), and predict solvent-dependent photophysical properties. Additional goals include characterizing bonding via QTAIM, NBO, and MESP analyses, evaluating interactions with the human HAP protein through docking and molecular dynamics, predicting ADMET pharmacokinetic profiles, and examining the specific roles of −NH_2_ and −NO_2_ groups in protein–ligand binding. Ultimately, this work aims to demonstrate how rational substituent engineering can fine-tune ESIPT/GSIPT behaviors, electronic and optical properties, receptor binding, and drug-likeness, positioning Cu-BSSMO derivatives as multifunctional candidates for optoelectronic, sensing, and therapeutic applications.

## 2 Computational Methodology

DFT has proven to be a reliable and efficient approach for analyzing system energies and examining the electronic structure of the ligand and its corresponding complex. Geometry optimizations were conducted using the B3LYP^53,54^ functional, employing the 6-31G­(d)^55^ basis set for C, H, O, N, S, F, Cl, and Br atoms, while the Cu center was treated with the LANL2DZ^56^ basis set. To validate the optimized structures, vibrational frequency analysis was carried out, confirming the absence of imaginary frequencies, thereby ensuring that the obtained geometries correspond to true energy minima and are thermodynamically stable. All the aforementioned calculations were performed using Gaussian 09 software.^57^ TD-DFT calculations were performed using the B3LYP functional with the 6-311+G­(d,p) basis set.^58^ Solvation effects were included using the SCRF method with the CPCM model.^59–61^ To assess and visualize noncovalent interactions (NCI) in BSSMO at both the S_0_ and S_1_ states, we carried out NCI analysis using Multiwfn 3.7.^62^ Furthermore, QTAIM analysis was performed using the AIM 2000 program package.^63–65^ Molecular docking analyses were conducted to evaluate the binding affinities of the ligand with the target protein, providing valuable insights into drug design. Molecular docking and molecular dynamics (MD) simulations were performed using Schrödinger software (Maestro 13.6).^66^ Docking studies were carried out with the Glide^67^ module in Schrödinger.^68^ The receptor grid was generated by centering it on the centroid of the cocrystallized ligand, and ligands were docked into the active site using the standard precision (SP) mode.^69^ The methodologies for docking are given in the Supporting Information (SI) (Section 1). Furthermore, ADMET and pharmacokinetic properties were predicted using the computational tool Quikproz.^70^ These in silico approaches facilitate the assessment of drug-like properties, bioavailability, and potential toxicity, aiding in the rational design of bioactive compounds. For molecular dynamics (MD) simulations, the enol forms of the NH_2_ (strong electron-donating) and NO_2_ (strong electron-withdrawing)-substituted BSSMO derivatives complexed with the target HAP protein were selected based on prior docking results. The simulations aimed to assess the stability and dynamic behavior of the ligands within the protein’s active site. Energy minimization of the protein–ligand complexes was first carried out using the steepest descent algorithm for 50,000 steps to remove steric clashes and optimize the geometry.^71–74^ The minimized systems were then equilibrated under the NVT ensemble (constant number of particles, volume, and temperature) for 100 ps, followed by the NPT ensemble (constant number of particles, pressure, and temperature) for another 100 ps, maintaining the system at 300 K and 1 bar. The V-rescale thermostat and Parrinello–Rahman barostat were applied with coupling constants of 0.1 and 2.0 ps, respectively, to ensure proper thermal and pressure equilibration. Production MD runs were performed for 100 ns using a 2 fs integration time step and the leapfrog integrator. Long-range electrostatics were treated using the Particle Mesh Ewald (PME) method with a Fourier spacing of 0.12 nm, while a 1.0 nm cutoff was applied for both van der Waals and short-range electrostatic interactions. All bonds involving hydrogen atoms were constrained by using the LINCS algorithm. Structural stability and conformational dynamics were analyzed through root-mean-square deviation (RMSD) and root-mean-square fluctuation (RMSF) over the 100 ns trajectory, providing insights into the flexibility and stability of the complexes and highlighting the impact of substituent electronic effects on protein–ligand interactions.^75,76^

## 3 Results and Discussion

In this study, we designed and analyzed a series of 10 molecules (Scheme 1), using a previously reported molecule as a reference.^31^ In this regard, we modified the terminal positions (R) by introducing EDGs and EWGs of varying strengths. These groups were selected according to their Hammett constant values. Scheme 1 illustrates the GSIPT and ESIPT mechanisms for these molecules. To assess the impact of different substituents, the reference molecule ^E^BSSMO_H_ was utilized, with further substitutions applied to explore substituent effects. Our focus extends beyond the GSIPT and ESIPT mechanisms, aiming to establish a relationship between various substituents and their corresponding Hammett sigma para constants­(σ_p_). The molecules are organized in the following order based on their EDG to EWG σ_p_ values: −NH_2_ (−0.57), −OCH_3_ (−0.28), −OH (−0.37), −CH_3_ (−0.14), −H (0), −Cl (+0.24), −Br (+0.26), −CF_3_ (+0.46), and −NO_2_ (+0.81). These molecules are labeled as ^E^BSSMO_R_ and ^K^BSSMO_R_, with “E” and “K” indicating the enol and keto forms, respectively, and “R” denoting the specific substituent. The excited-state forms are denoted as ^*E^BSSMO_R_ and ^*K^BSSMO_R_, respectively.

**Scheme 1:**
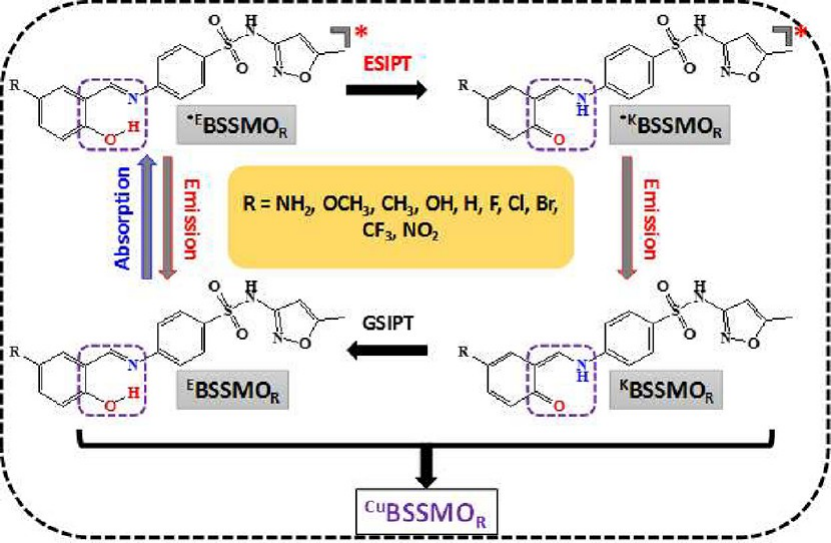
Mechanism of ESIPT and GSIPT in BSSMO Derivatives with Various Substituents, Including EDG and EWG Groups

### 3.1 Optimized Geometry

The optimized geometries of BSSMO derivatives in both their enol and keto forms were analyzed using DFT calculations, with a particular focus on selected bond lengths related to O–H and N–H bonds. The hydrogen-bonding interactions (O–H···N and O···H–N) in the ground state (S_0_) are illustrated in Figure 1 (enol form) and in the Supporting Information (Figure S1, keto Form). Additionally, the bond lengths for the non-ESIPT form and for the excited state (S_1_) are provided in the Supporting Information (Figures S2–S4). The hydrogen-bond distances (O–H···N and N–H···O) indicate that these molecules are stabilized by intramolecular hydrogen bonding in both the enol and keto forms at the S_0_ and S_1_ states with various substituent groups involved. In the keto form, the N_1_–H_1_ and the O_1_–H_1_ bond lengths show a noticeable increase when moving from EDGs to EWGs. For instance, in the enol form, the bond lengths are 0.99 and 1.77 Å for NH_2_ and 1.00, 1.74 Å for NO_2_, whereas in the keto form, they increase to 1.04 and 1.71 Å for NH_2_ and 1.04 and 1.69 Å for NO_2_. Meanwhile, the C_1_–O_1_ bond length decreases from 1.34 Å (NH_2_) and 1.33 Å (NO_2_) in the enol form to 1.26 Å (NH_2_) and 1.25 Å (NO_2_) in the keto form. Notably, the bond length of O_1_–H_1_ gradually increases from −NH_2_ to −NO_2_ substitutions, such as from 0.99 to 1.00 Å in the enol form at the S_0_ state.

**Figure 1:**
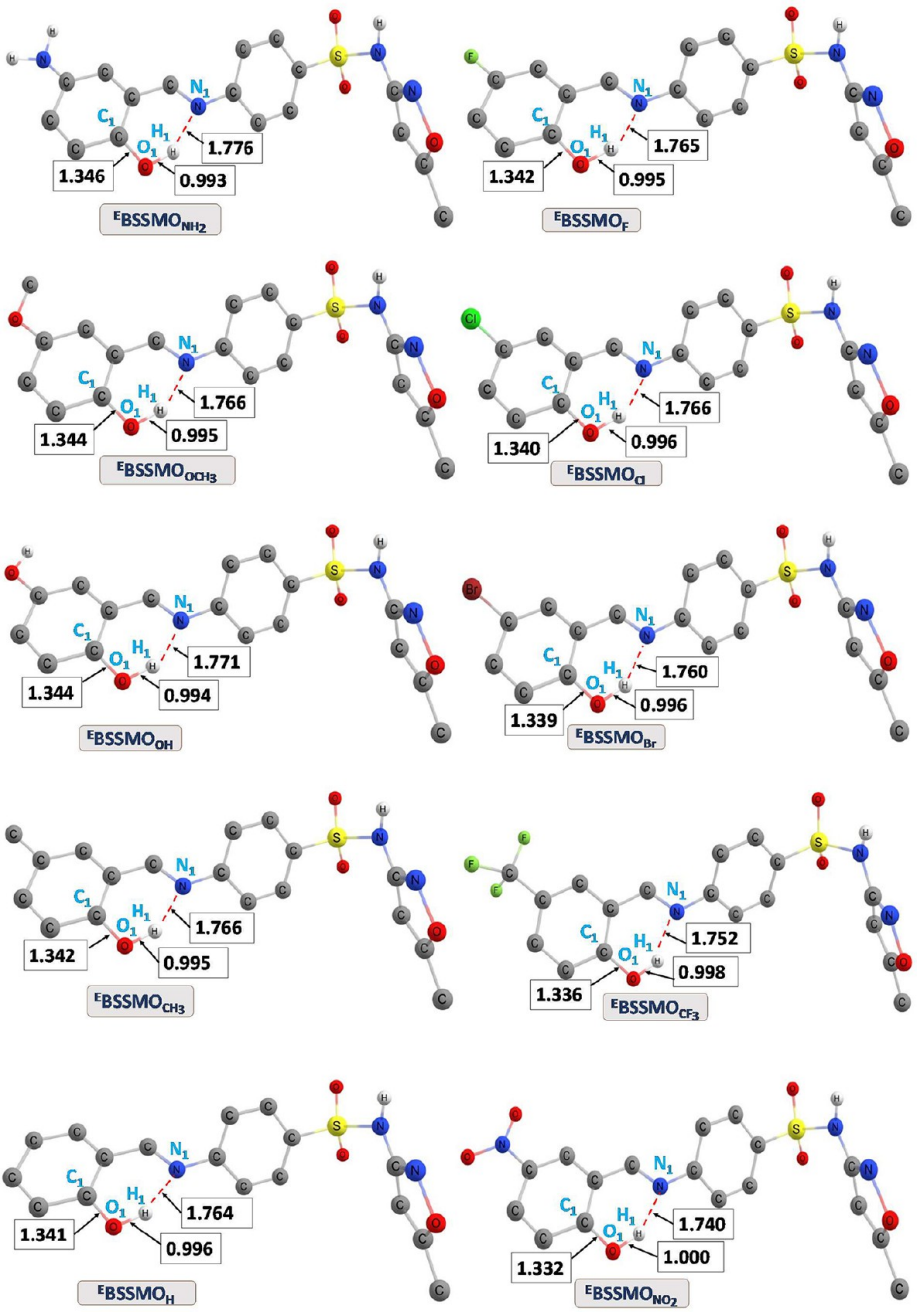
Selected bond lengths for the optimized ground-state geometries of various substituents in their enol (^E^BSSMO_R_) forms in the gas phase in the S_0_ state.

Similarly, the hydrogen-bonding interaction of O_1_–H_1_···N_1_ for all of the substitutions in BSSMO derivatives is found to be strengthened. These variations enhance the proton transfer ability from the enol to the keto form, as observed in bond length variations from 1.77 Å for ^E^BSSMO_NH2_ to 1.74 Å for ^E^BSSMO_NO2_. Conversely, the bond length increases progressively from EDG to EWG substituents. This trend suggests that the EWG strengthens hydrogen-bonding interactions, leading to the elongation of these bonds. Additionally, the C_1_–O_1_ bond length gradually decreases from EDGs (1.26 Å for ^K^BSSMO_NH2_ and 1.26 Å for ^K^BSSMO_OH_) to EWGs (1.25 Å for ^K^BSSMO_CF3_ and 1.25 Å for ^K^BSSMO_NO2_), indicating an increased double-bond character in the keto form due to electronic effects. Furthermore, in the enol form, both the N_1_–H_1_ and C_1_–O_1_ bond lengths exhibit decreasing trends, further demonstrating the influence of substituents on molecular geometry. The variations in N_1_–H_1_ bond lengths follow the order: ^E^BSSMO_NH2_ (1.77 Å) > ^E^BSSMO_OCH3_ (1.76 Å) > ^E^BSSMO_H_ (1.76 Å) > ^E^BSSMO_Br_ (1.76 Å) > ^E^BSSMO_CF3_ (1.75 Å) > ^E^BSSMO_NO2_ (1.74 Å). Similarly, the decreasing trend in C_1_–O_1_ bond lengths is observed as ^E^BSSMO_NH2_ (1.34 Å) > ^E^BSSMO_OCH3_ (1.34 Å) > ^E^BSSMO_CH3_ (1.34 Å) > ^E^BSSM_OH_ (1.34 Å) > ^E^BSSMO_Br_ (1.33 Å) > ^E^BSSMO_CF3_ (1.33 Å) > ^E^BSSMO_NO2_ (1.33 Å). The presence of EWGs enhances hydrogen-bonding interactions, inducing structural adjustments that stabilize both the enol and keto forms. In the S_1_ state, the N_1_–H_1_ and C_1_–O_1_ bonds contract, whereas the O_1_–H_1_ bond elongates (SI, Figures S3 and S4). In both tautomeric forms, transitioning from EDGs to EWGs leads to an increase in the O_1_–H_1_ and N_1_–H_1_ bond lengths, while the C_1_–O_1_ bond length decreases due to the push–pull effect. In the keto form, the O_1_–H_1_ and N_1_–H_1_ bonds are further elongated compared to the enol form in the S_0_ state, an effect attributed to the push–pull interaction governing hydrogen bonding. This trend is consistent with the nature of hydrogen-bonding interactions: O–H···N in the atoms was the enol form, and the other was the keto form.

The stronger hydrogen bonding in the keto form enhances molecular reactivity relative to the enol form. Notably, within the keto tautomer, EDGs result in longer bond distances than EWGs. Consequently, molecules bearing EWGs may exhibit a higher reactivity than those with EDGs. The infrared (IR) stretching frequencies of the molecules in both the enol and keto forms are presented in Table 1. It indicates that the enol form exhibits higher vibrational frequencies than the keto form across all substituents, with ^K^BSSMO_NH2_ displaying the highest frequency (3275 cm^–1^; force constant: 6.74 mdyn Å^–1^) and ^K^BSSMO_NO2_ the lowest (3150 cm^–1^; force constant: 6.25 mdyn Å^–1^). This trend suggests a weakening of the hydrogen bond of O–H···N in the keto form. The force constant follows a decreasing order from NH_2_ (6.74 mdyn Å^–1^) to NO_2_ (6.25 mdyn Å^–1^) in the enol form and from NH_2_ (6.20 mdyn Å^–1^) to NO_2_ (5.95 mdyn Å^–1^) in the keto form, indicating that EWGs such as CF_3_ and NO_2_ attenuate hydrogen bonding and enhance structural flexibility.

**Table 1:** Selected Bond Lengths (O–H and N–H) of Vibrational Frequencies for the Optimized Ground-State Geometries of Various Substituents in Their Enol and Keto Forms of BSSMO_R_ in the Gas Phase in the S_0_ State

	O–H stretching from enol forms			N–H stretching from keto forms		
substituents (R) (BSSMO_R_)	frequency (cm^–1^)	force constant	IR intensity	frequency (cm^–1^)	force constant	IR intensity
NH_2_	3275	6.74	373.89	3113	6.20	81.75
OCH_3_	3247	6.63	374.11	3058	5.98	82.95
OH	3260	6.69	373.11	3109	6.18	87.04
CH_3_	3238	6.60	447.88	3075	6.05	115.78
H	3234	6.58	458.01	3052	5.96	125.16
F	3243	6.61	421.67	3079	6.06	94.22
Cl	3223	6.55	431.96	3071	6.03	105.24
Br	3221	6.55	415.09	3072	6.04	106.18
CF_3_	3192	6.41	523.99	3064	6.00	124.23
NO_2_	3150	6.25	642.07	3021	5.95	131.25

The IR absorption intensity is notably higher for molecules with EWGs, with the NO_2_ substituent exhibiting the most pronounced absorption intensity (642.07 in the enol form and 131.25 in the keto form), reflecting significant dipole moment variations. In contrast, EDGs such as NH_2_ and OCH_3_ reinforce hydrogen bonding, leading to increased O–H stretching frequencies and force constants in the S_0_ state. The observed trend suggests that EWGs preferentially stabilize the keto form, whereas EDGs favor the enol form, thereby modulating the tautomeric equilibrium in the S_0_ state. Furthermore, the variation in BSSMO before and after photoexcitation correlates with the IR vibrational peak of the O_1_–H_1_ bond in the S_0_ state. Notably, the IR stretching frequencies of the enol and keto forms of the O_1_–H_1_ and N_1_–H_1_ bonds exhibit a shift from stronger to weaker relative to those observed in the BSSMO derivatives of the enol and keto forms, respectively. These findings align well with structural analyses, reinforcing the proposed effects of substituent-induced electronic influences on hydrogen bonding and tautomeric stability.

### 3.2 Frontier Molecular Orbitals (FMOs)

The frontier molecular orbitals (FMOs) play a pivotal role in analyzing the excitation properties of ESIPT and GSIPT molecules. To comprehensively understand the mechanism underlying hydrogen bond strengthening, it is crucial to examine the distribution of FMOs.^31,49,54,77,78^ In this regard, the highest occupied molecular orbitals (HOMOs) and lowest unoccupied molecular orbitals (LUMOs) for the S_0_ state in both the enol and keto forms (Figures 2 and 3) as well as the non-ESIPT enol form are given in the SI (Figure S5). To gain a deeper understanding, the computed FMOs, including HOMO–1, HOMO, LUMO, and LUMO+1 in the S_0_ state, were thoroughly analyzed and discussed. The HOMO is primarily stabilized on the tautomeric unit in the BSSMO derivatives along with contributions from the substituents. In contrast, the LUMO and LUMO+1 are predominantly stabilized throughout the entire molecule, except for the substituent units. A similar stabilization pattern is observed in the keto forms of the BSSMO derivatives.

**Figure 2:**
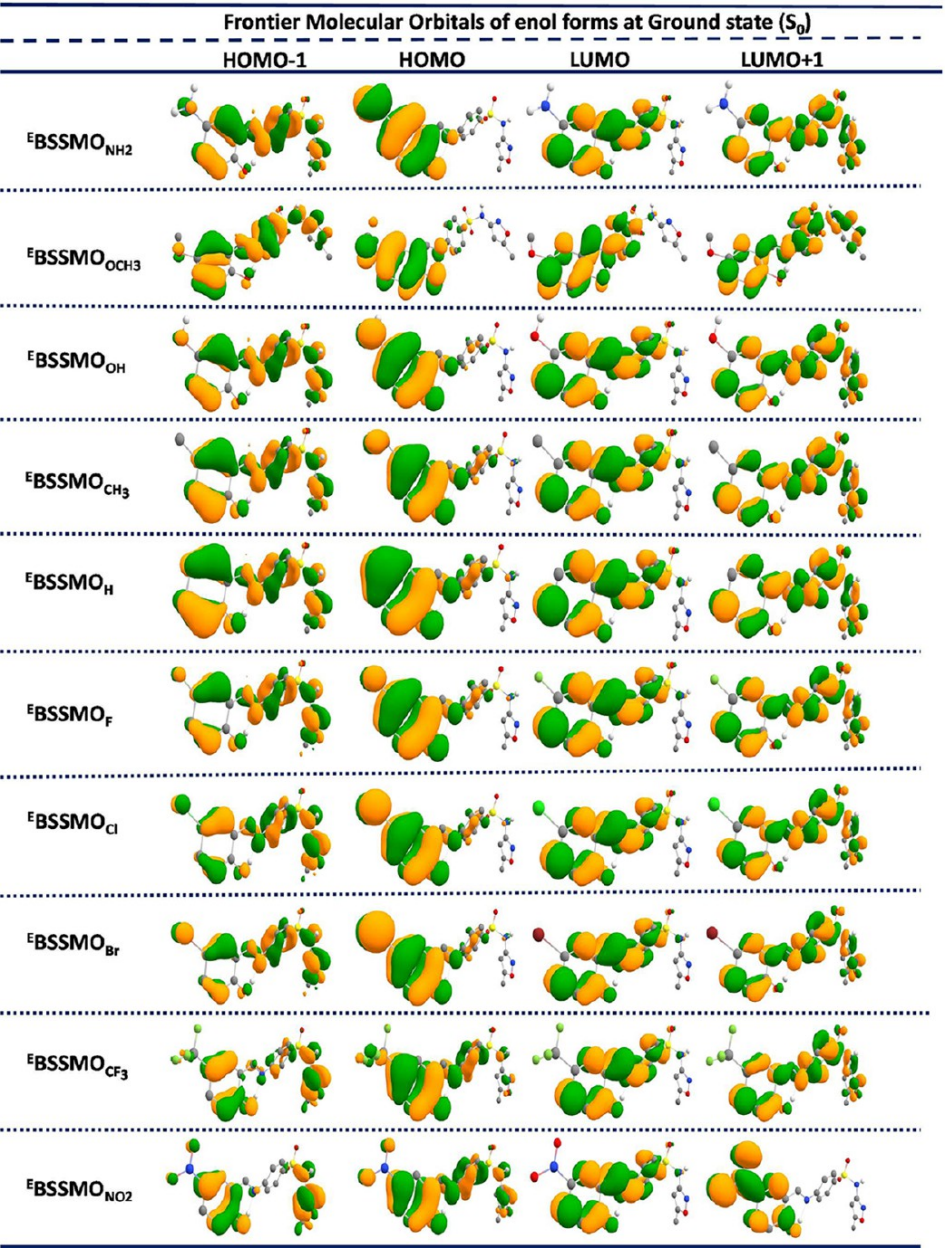
Frontier molecular orbitals (FMOs) for the enol forms of all substituents (^E^BSSMO_R_) in the gas phase in the ground state (S_0_).

**Figure 3:**
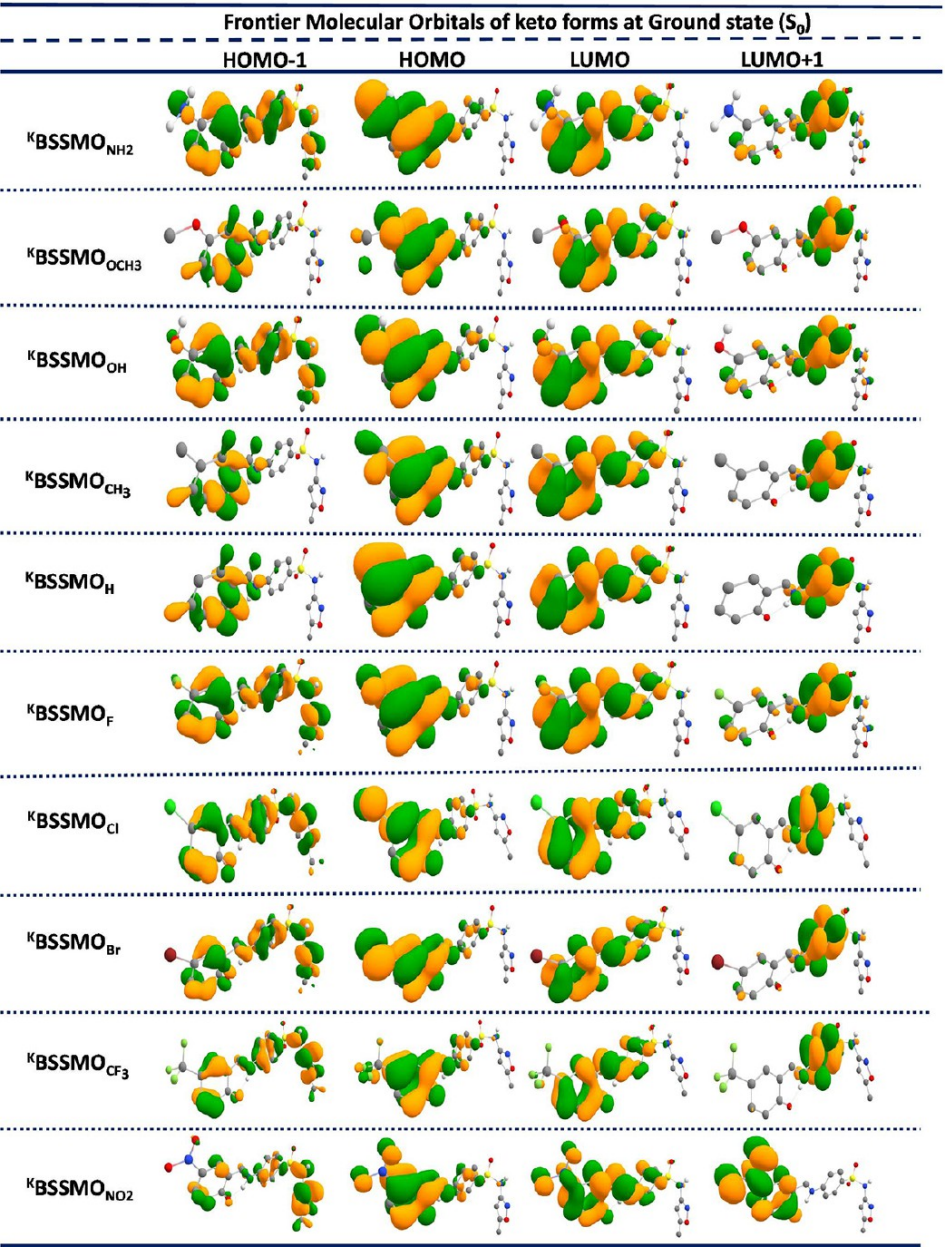
Frontier molecular orbitals (FMOs) for the keto forms of all substituents (^K^BSSMO_R_) in the gas phase in the ground state (S_0_).

The FMOs’ energy levels and the energy gap (*E*_g_) for the enol, keto, and Cu-BSSMO molecules are depicted in Figure 4 and Tables S1–S4 (SI). As shown in Figures 2 and 3, the EDGs increase the electron density in the molecule, thereby elevating the HOMO levels, which is reflected in the *E*_g_ values for all BSSMO derivatives. Among all of the molecules, the enol (Non-ESIPT) forms exhibit the highest *E*_g_. Specifically, for the EWGs, the gaps are as follows: 4.29 eV for ^Non‑ESIPT^BSSMO_H_, 4.17 eV for ^Non‑ESIPT^BSSMO_F_, 4.19 eV for ^Non‑ESIPT^BSSMO_Cl_, 4.25 eV for ^Non‑ESIPT^BSSMO_CF3_, and 4.00 eV for ^Non‑ESIPT^BSSMO_NO2_. Similar trends are observed in the enol (^E^BSSMO_R_) and keto (^K^BSSMO_R_) forms of the molecules. For both the enol and keto forms, the presence of EDGs results in a reduction of the *E*_g_. Specifically, BSSMO_NH2_ exhibits an *E*_g_ of 3.23 eV for the enol form and 2.67 eV for the keto form, which further decreases to 2.16 eV in ^Cu^BSSMO_NH2_. As discussed in our previous work,^31^ the incorporation of the −Br substituent into the ^E^BSSMO_Br_ and ^K^BSSMO_Br_ molecules leads to higher *E*_g_, with the LUMO values being predominantly higher in both the enol and keto forms. From Figure 4, ^Cu^BSSMO_R_ (D) is evident that EDGs result in smaller energy gaps compared to EWGs, particularly in copper complex formations. ^Cu^BSSMO_NH2_ exhibits an *E*_g_ of 2.16 eV, with the LUMO levels progressively decreasing in energy from ^Cu^BSSMO_NO2_, as clearly illustrated in Figure 4. The lower *E*_g_ in EDGs is attributed to their increased reactivity, which facilitates the formation of more stable copper complexes. However, this enhanced reactivity trend is more pronounced in EDGs than in EWGs in both enol and keto forms. Schiff base copper complexes demonstrate significant interactions between EDGs and EWGs, which promote efficient electron transfer within the molecule.

**Figure 4:**
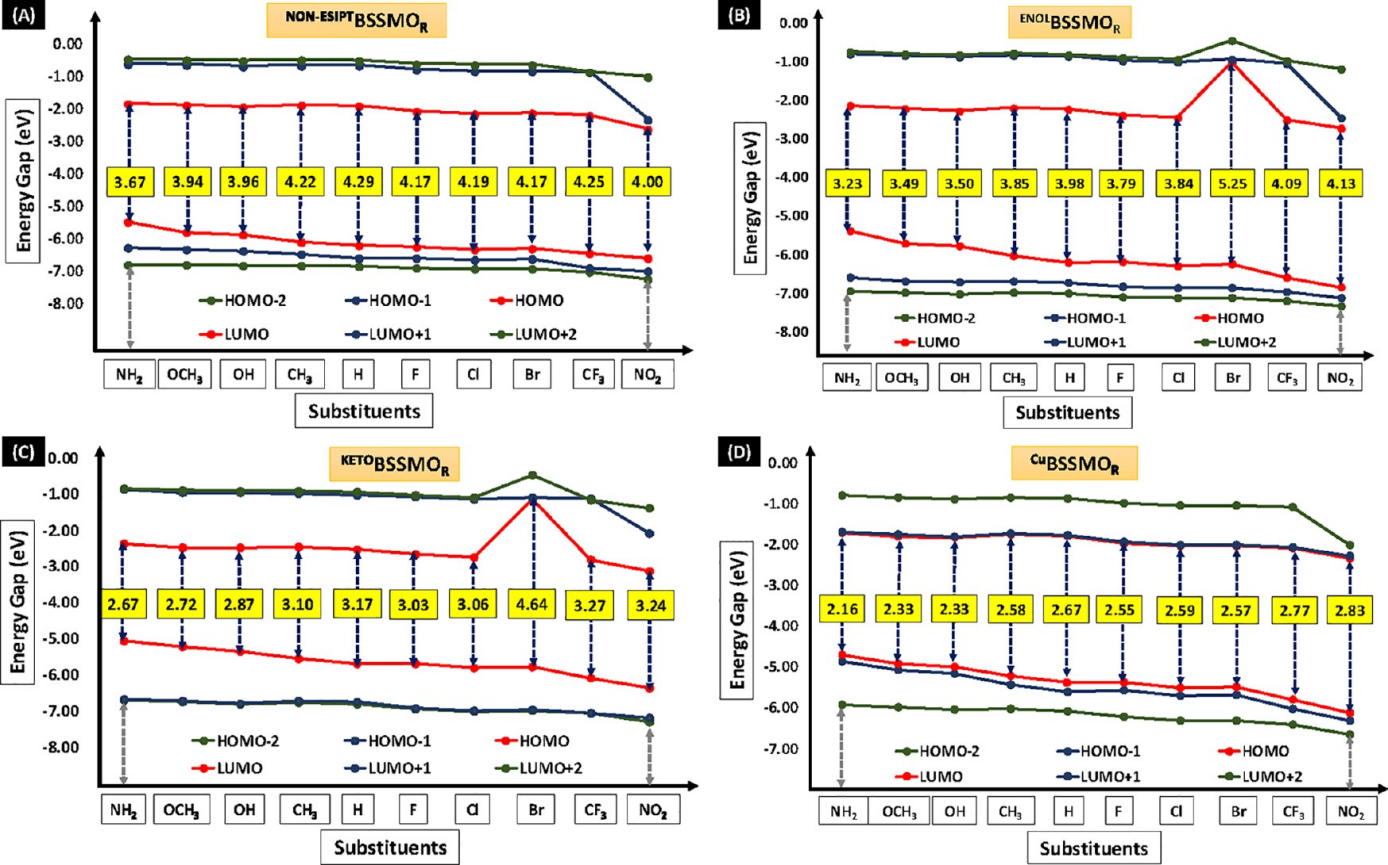
Energy gap (*E*_g_ in eV) for enol (Non-ESIPT, (A)), enol (ESIPT, (B)), keto (ESIPT, (C)) and Cu-BSSMO forms (D) of substituted BSSMO derivatives at the S_0_ state in the gas phase.

EDGs such as −NH_2_, −OCH_3_, −OH, and −CH_3_ lower the HOMO energy, thereby enhancing electron donation, narrowing the *E*_g_ and facilitating charge transfer. Additionally, these EDGs increase the molecule’s electron-donating ability, enhancing the stability of metal coordination. This is attributed to their relatively low intramolecular charge transfer ability. In the S_1_ state of the keto forms, similar *E*_g_ are observed across all EWG derivatives compared to the enol forms, indicating the formation of highly stabilized keto forms that favor the ESIPT mechanism. In contrast, for EDG derivatives, the increased reactivity of the enol forms in the S_1_ state is due to reduced *E*_g_. The *E*_g_ decreases in the excited state, and EDGs are believed to influence both the intramolecular hydrogen bonding and ESIPT processes.^49^ In contrast, the opposite trend is observed with the introduction of EWGs, suggesting that ESIPT fluorescence can be tuned only by EDG substituents. To further explore the effects of substituents, the mechanisms of the ESIPT and GSIPT processes were exhibited by using transition state theory calculations.

### 3.3 Comparative Mechanistic Insights into ESIPT and GSIPT Processes in Substituted BSSMO Derivatives

A detailed computational investigation of the ESIPT and GSIPT intramolecular proton transfer processes in BSSMO derivatives has been performed, following similar approaches to those reported in previous studies.^31,35,45,49,79–84^ This investigation reveals distinct substituent-dependent behaviors that modulate proton transfer kinetics and thermodynamics. For the ESIPT process, transition state (TS) calculations show that the activation barriers (SI Tables S5 and S6) are significantly influenced by the electronic nature of the substituents. The potential energy landscape, transition states (*^TS^BSSMO_R_), and corresponding products (*^K^BSSMO_R_) for the ESIPT mechanism of EDG/EWG-substituted derivatives were computed (at the S_1_ state) in the gas phase (Figure 5). EDGs such as NH_2_, OCH_3_, OH, and CH_3_ exhibit higher energy barriers, ranging from 6.09 to 7.28 kcal/mol, indicating reduced ESIPT efficiency due to intramolecular stabilization of the enol form (refer to Figure 5). Notably, BSSMO_NH2_ displays the highest ESIPT barrier (7.28 kcal/mol), reflecting a strong resistance to proton transfer in the excited state. Conversely, EWGs such as CF_3_ and NO_2_ significantly lower the TS barrier with values of 4.94 and 4.44 kcal/mol, respectively. These groups facilitate ESIPT by increasing proton acidity and stabilizing the charge-separated transition state. The keto products formed via ESIPT are also more stabilized in the presence of EWGs, as seen in BSSMO_NO2_ (2.42 kcal/mol) and BSSMO_CF3_ (2.97 kcal/mol), compared to EDG-substituted derivatives such as BSSMO_OCH3_ (4.63 kcal/mol). In contrast, for the GSIPT process, activation barriers are universally low, typically between 0.31 and 0.74 kcal/mol, indicating facile proton transfer across all substituents.

**Figure 5:**
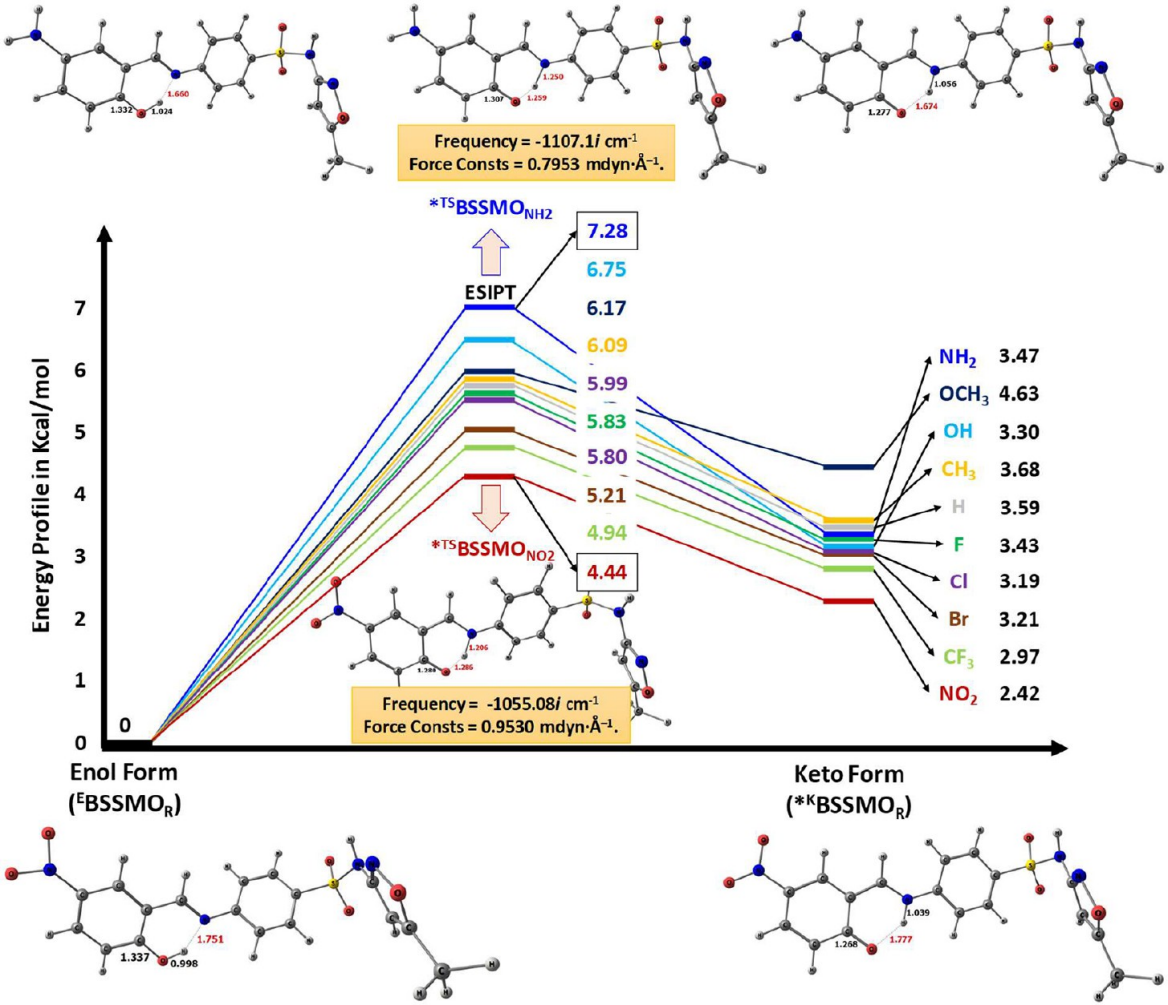
Potential energy landscape, transition states (*^TS^BSSMO_R_), and corresponding products (*^K^BSSMO_R_) for the ESIPT mechanism of EDG/EWG-substituted derivatives in the gas phase at the S_1_ state. The estimated energies, referenced to the enol form of the *^E^BSSMO_R_ reactants, are reported in kcal/mol.

However, subtle trends emerge: EDGs of OH, CH_3_, and NH_2_ show relatively higher GSIPT barriers (SI Table S6), suggesting minor resistance due to stabilization of the enol form. An exception is BSSMO_OCH3_, which shows a barrierless transition potentially due to specific stabilizing interactions. EWGs of CF_3_ and NO_2_ yield slightly lower GSIPT barriers (0.51–0.62 kcal/mol), although the difference from EDGs is less pronounced than in ESIPT. From a thermodynamic perspective, all keto forms are more stable than their enol counterparts, with relative energies ranging from −2.42 to −4.63 kcal/mol. The most stabilized keto product is observed for BSSMO_OCH3_, while BSSMO_NO2_ forms the least stable keto tautomer, indicating that strong EWGs might slightly reduce thermodynamic favorability despite lowering the activation barrier.

Overall, the ESIPT process is more sensitive to the electronic nature of the substituents than GSIPT, with EWGs significantly enhancing both the reaction kinetics and product stability in the excited state. In contrast, GSIPT remains a low-barrier process across all derivatives, with only minor variations due to substituent effects, primarily affecting the thermodynamic stability of the keto product rather than the activation energy. These mechanistic insights emphasize that EWGs are beneficial for promoting ESIPT, while EDGs enhance the ground-state keto stability. This differential control over proton transfer dynamics offers a valuable strategy for the rational design of BSSMO-based materials, particularly in photoresponsive systems, molecular electronics, and biologically relevant tautomeric switches.

### 3.4 Electronic Properties: Absorption and Emissions

TD-DFT plays a pivotal role in the investigation of electronic properties by enabling accurate prediction of excited-state energies, which are essential for understanding absorption and emission processes.^85,86^ In contrast to ground-state DFT, TD-DFT accounts for the dynamics of electronic excitations, facilitating the calculation of absorption wavelengths (λ_abs),_ emission energies, oscillator strengths (*f*_0_), and the detailed characterization of electronic transitions. The UV–visible λ_abs_ and fluorescence spectra of the molecules in both their S_0_ and S_1_ states were computed based on the optimized geometries of their normal and tautomeric forms (Figure 6). These computational results were compared with experimental data from a previous study.^31^ Additionally, the effects of various substituents on the absorption spectra in dimethylformamide (DMF, ε = 36.7), dichloromethane (DCM, ε = 8.9), toluene­(ε = 2.4), and cyclohexane­(ε = 2.02) solvents were investigated (SI, Tables S7–S10). The electronic spectroscopic data for a series of substituted compounds in the gas phase were analyzed in three categories: enol absorption (λ_abs_), enol emission (λ_ems_), and keto emission (λ_ems_). The λ_abs_ (enol forms) and λ_ems_ (enol and keto forms) of BSSMO derivatives were computed in the gas phase using the B3LYP/6-311+G­(d,p) level of theory, yielding spectral peak positions at λ1, λ2, λ3, and λ4, along with their corresponding oscillator strengths (*f*_0_), as shown in Figure 6 and Table 2.

**Table 2:** Calculated Electronic Properties [λ_abs_, λ_ems_ in nm, *f*_0_] of BSSMO Derivatives at B3LYP/6-311+G (d, p) in the Gas Phase along with the Respective δ_p_

enol form of ^E^BSSMO_R_ (λ_abs,_ nm) at gas phase										
R	NH_2_	OCH_3_	OH	CH_3_	H	F	Cl	Br	CF_3_	NO_2_
λ1	449.195	408.763	402.220	370.570	354.770	366.660	366.620	368.970	341.660	356.739
*f*_0_	0.1688	0.2839	0.279	0.3777	0.4850	0.4245	0.3939	0.3636	0.6113	0.2419
λ2	322.118	318.086	320.544	317.885	315.545	318.344	318.867	319.710	308.530	338.353
*f*_0_	0.7627	0.6014	0.6057	0.5829	0.4445	0.4099	0.4573	0.4995	0.2020	0.6716
λ3	282.523	289.184	277.810	284.050	282.235	280.833	242.990	280.970	274.640	319.246
*f*_0_	0.1789	0.0920	0.1221	0.1995	0.2207	0.1876	0.2099	0.1682	0.2576	0.1927
λ4	261.985	278.227	245.074	216.540	214.557	214.305	214.312	245.381	210.510	266.923
*f*_0_	0.1648	0.1434	0.1375	0.1610	0.1014	0.2388	0.1816	0.2171	0.1322	0.1719

enol form of * ^**E**^ **BSSMO**_**R**_ **(λ**_**ems,**_ **nm) at gas phase**										
**R**	**NH**_**2**_	**OCH**_**3**_	**OH**	**CH**_**3**_	**H**	**F**	**Cl**	**Br**	**CF**_**3**_	**NO**_**2**_
λ1	637.197	519.880	514.490	487.538	425.974	464.376	443.185	467.736	404.243	375.709
*f*_0_	0.1333	0.2355	0.2141	0.1952	0.2997	0.2628	0.2947	0.2549	0.4165	0.0990
λ2	347.940	340.086	343.538	348.139	346.393	343.457	345.286	345.615	339.935	337.880
*f*_0_	0.7365	0.9634	0.9811	1.0226	0.9305	0.8935	0.9299	0.9435	0.7481	0.8077
λ3	344.430	247.380	253.321	248.470	235.350	240.869	240.757	243.632	224.408	269.107
*f*_0_	0.2575	0.0853	0.0968	0.1578	0.0988	0.1060	0.0966	0.1386	0.0210	0.2551
λ4	340.450	231.521	235.500	237.065	236.180	233.794	233.639	223.172	209.207	265.483
*f*_0_	0.1402	0.1090	0.0994	0.0705	0.0295	0.0832	0.1407	0.1355	0.1716	0.2580

keto form of * ^**K**^ **BSSMO**_**R**_ **(λ**_**ems,**_ **nm) at gas phase**										
**R**	**NH**_**2**_	**OCH**_**3**_	**OH**	**CH**_**3**_	**H**	**F**	**Cl**	**Br**	**CF**_**3**_	**NO**_**2**_
λ1	676.789	613.020	588.204	559.442	534.221	536.231	536.460	534.490	541.961	512.438
*f*_0_	0.2508	0.2604	0.2665	0.2278	0.2139	0.2726	0.2744	0.2844	0.1951	0.3069
λ2	345.401	353.216	342.021	348.150	348.283	344.561	345.429	345.481	346.207	353.720
*f*_0_	0.8987	0.0794	0.2716	0.8537	0.8457	0.7080	0.7495	0.7680	0.7078	0.3966
λ3	284.788	346.07	348.325	329.560	252.099	312.706	314.383	314.068	321.391	347.367
*f*_0_	0.0320	0.1352	0.3095	0.0663	0.0662	0.0863	0.1253	0.1223	0.0834	0.3849
λ4	243.624	338.243	334.947	263.285	239.373	237.252	236.702	237.439	244.520	333.846
*f*_0_	0.2062	0.7426	0.3338	0.0800	0.0087	0.0960	0.1437	0.2084	0.1186	0.2591

**Figure 6:**
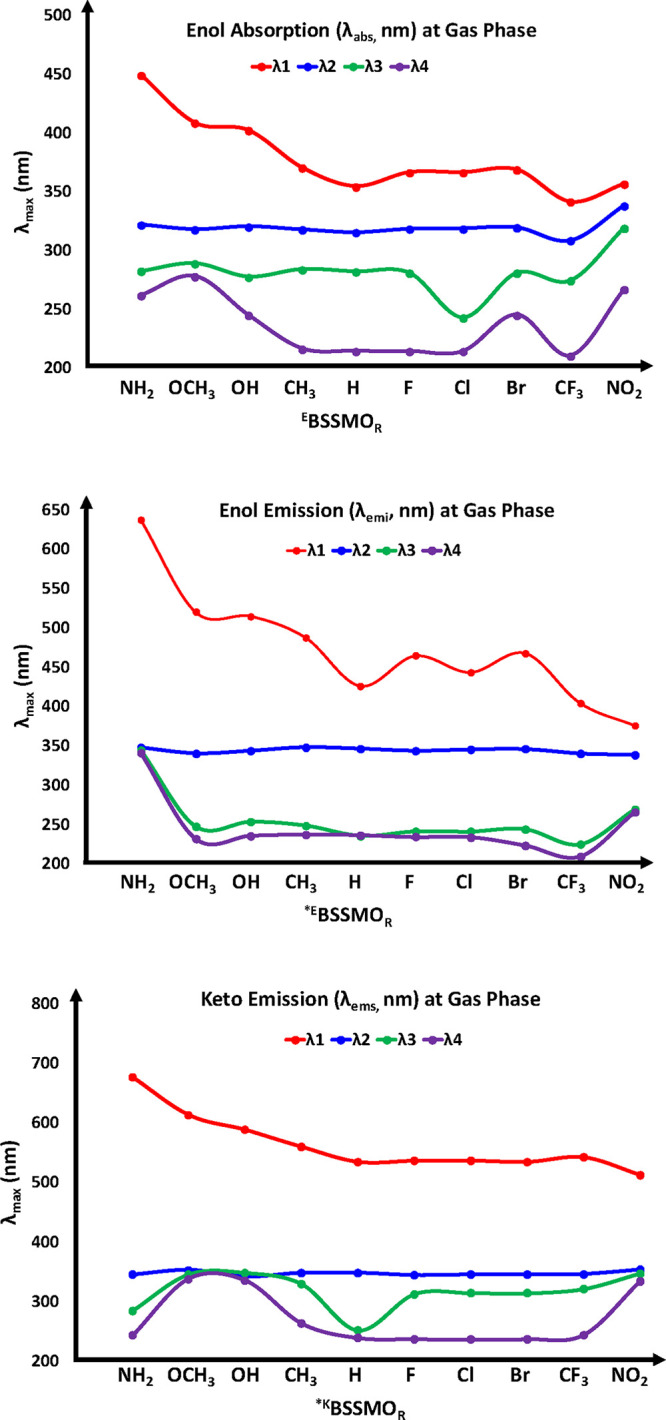
Absorption (enol forms) and emission (enol and keto forms) spectral peaks (λ1, λ2, λ3, and λ4) of BSSMO derivatives at the B3LYP/6-311+G­(d,p) level in the gas phase.

As shown in Table 2, for enol absorption (^E^BSSMO_R_): λ1 range from 449 nm (for NH_2_) to 341 nm (for CF_3_), with intensity differences indicated by *f*_0_ values. EWGs such as ^E^BSSMO_CF3_ and ^E^BSSMO_NO2_ exhibit stronger absorption at λ1, while ^E^BSSMO_NH2_ shows the weakest absorption. The absorptions at λ2 and λ3 exhibit relatively consistent patterns across different substituents, suggesting a common underlying mechanism for the π→π* and ICT transitions. As shown in Table 2, for enol emission (^*E^BSSMO_R_): emission maxima (λ1) range from 637 nm for NH_2_ to 375.7 nm for NO_2_. EDGs such as ^*E^BSSMO_NH2_ and ^*E^BSSMO_OCH3_ tend to exhibit longer emission wavelengths. The intensity at λ1 (*f*_0_) is greatest for ^*E^BSSMO_F_, ^*E^BSSMO_Cl_, and ^*E^BSSMO_CF3_ substituents, with ^*E^BSSMO_NH2_ and ^*E^BSSMO_NO2_ showing weaker emission intensities. The second emission maximum (λ2) shows stronger intensities for the ^*E^BSSMO_OCH3_ and ^*E^BSSMO_OH_ groups, suggesting that these substituents enhance the emission efficiency.

As shown in Table 2, for keto emission (^*K^BSSMO_R_): keto emission maxima (λ1) are at the longest wavelengths for the NH_2_ and OCH_3_ groups (676 and 613 nm), and the shortest wavelengths are found for NO_2_ (512.4 nm). The emission intensity at λ1 (*f*_0_) remains relatively consistent across different substituents, with ^*K^BSSMO_NH2_ and ^*K^BSSMO_OCH3_ showing higher intensities compared to those of ^*K^BSSMO_CF3_ and ^*K^BSSMO_NO2_. The spectra at shorter wavelengths (λ4) show significant variations, especially for ^*K^BSSMO_NH2_ and ^*K^BSSMO_OCH3,_ which shift toward higher energies.

Overall, the electronic spectral data (at the gas phase) highlight the significant impact of substituents on the electronic and photophysical behavior of the enol and keto forms. EDGs (NH_2_, OCH_3_) lead to bathochromic (red) shifts in both absorption and emission spectra, while strong EWGs (NO_2_, CF_3_) induce hypsochromic (blue) shifts. To provide a quantitative understanding of the bathochromic and hypsochromic shifts, the oscillator strengths (*f*_0_) of BSSMO derivatives were analyzed in both enol and keto forms across different solvents (gas phase, DMF, DCM, toluene, and cyclohexane).

In the gas phase, enol absorption (λ_1_) shows higher *f*_0_ values for EWGs such as CF_3_ (0.6113) and moderate values for EDGs like NH_2_ (0.1688), while enol emission (λ_1_) is enhanced by EDGs (NH_2_- 0.1333; OCH_3_- 0.2355) and weaker for EWGs. Keto emission (λ_1_) exhibits higher intensities for EDGs (NH_2_- 0.2508; OCH_3_- 0.2604) than EWGs (CF_3_- 0.1951; NO_2_- 0.3069). In polar solvents such as DMF, *f*_0_ values for absorption increase slightly for polarizable substituents (NH_2_- 0.1771; OCH_3_- 0.2966; CF_3_- 0.633), while enol and keto emission trends remain consistent. Moderately polar DCM and low-polar toluene display similar patterns, with EWGs showing strong absorption (CF_3_- 0.627; NO_2_- 0.328) and EDGs favoring emission (NH_2_- 0.142; OCH_3_: 0.246). In nonpolar cyclohexane, the trends persist, highlighting intrinsic substituent effects, with EDGs enhancing emission (NH_2_- 0.153; OCH_3_- 0.256) and EWGs favoring absorption (CF_3_-0.614; NO_2_- 0.748). Overall, these *f*_0_ values quantitatively explain the variations in transition intensities across solvents, showing that EDGs generally increase emission intensity, whereas EWGs strengthen absorption, providing a mechanistic understanding of substituent-dependent photophysical behavior in BSSMO derivatives.

### 3.5 Solvent Effects on Electronic Spectra of Absorption and Emissions

Solvent polarity plays a crucial role in modulating the electronic absorption and emission spectra of molecules.^87–90^ TD-DFT was employed to investigate how solvents with different dielectric constants—DMF (ε = 36.7), DCM (ε = 8.9), toluene (ε = 2.4), and cyclohexane (ε = 2.02)—affect the spectral behavior of the studied compound. This analysis helps understand solvent-induced spectral shifts and the influence of the solvent environment on excited-state properties.^91–94^ For Table S7 (SI), in DMF solvent (ε = 36.7), the absorption and emission spectra for the enol form exhibited the following trends: (i) enol absorption (λ_abs_, nm): ^E^BSSMO_NH2_ showed the longest λ1 (450.3 nm), indicative of an electron-donating effect that stabilizes the LUMO and lowers excitation energy. In contrast, ^E^BSSMO_CF3_ (342.87 nm) and ^E^BSSMO_NO2_ (356.8 nm) exhibited blue shifts due to their electron-withdrawing effects. The highest *f*_0_ for λ1 was observed for ^E^BSSMO_CF3_ (0.633), indicating a strong transition probability despite the blue shift. Higher transitions (λ3 and λ4) were found in the UV region (262–289 nm), with ^E^BSSMO_CF3_ showing the highest *f*_0_ (0.2629). (ii) Enol emission (λ_ems_, nm): ^*E^BSSMO_NH2_ exhibited the longest λ1 emission (639.21 nm), while ^*E^BSSMO_NO2_ showed the lowest λ1 emission (370.28 nm). ^*E^BSSMO_CF3_ exhibited the highest *f*_0_ for λ1 (0.4393), indicating intense emission despite the shorter wavelength. λ2 emission peaks were stable (336–350 nm), with the highest *f*_0_ observed for ^*E^BSSMO_CH3_ (1.0385), ^*E^BSSMO_OH_ (0.997), and ^*E^BSSMO_OCH3_ (0.9766). Emission transitions λ3 and λ4 showed lower *f*_0_ values, with ^*E^BSSMO_NO2_ exhibiting a higher *f*_0_ value (0.2186), potentially due to ICT. (iii) Keto emission (λ_ems_, nm): the longest λ1 emission was observed for ^*K^BSSMO_NH2_ (681.24 nm), indicating stabilization of the keto form via intramolecular proton transfer. ^*K^BSSMO_NO2_ exhibited the shortest λ1 emission (510.18 nm) due to its electron-withdrawing effect. *f*_0_ was moderate across all compounds, with ^*K^BSSMO_NO2_ and ^*K^BSSMO_Br_ showing higher *f*_0_ values (0.3369 and 0.2952, respectively). λ2 transitions remained consistent (345–356 nm), with ^*K^BSSMO_CH3_ showing the highest *f*_0_ (0.8766). λ3 transitions (285–348 nm) were dominated by ^*K^BSSMO_NH2_, with the highest *f*_0_ (0.916) observed for this substituent. In DCM solvent (ε = 8.9), the absorption and emission spectra exhibited the following characteristics (SI, Table S8): (i) enol absorption (λ_abs_, nm): ^E^BSSMO_NH2_ showed the longest λ1 absorption (450.32 nm), while ^E^BSSMO_CF3_ (343.47 nm) and ^E^BSSMO_NO2_ (353.8 nm) exhibited blue shifts. The *f*_0_ values were highest for ^E^BSSMO_CF3_ (0.627) and ^E^BSSMO_H_ (0.504), indicating stronger transitions. λ2 ranged from 307–323 nm, with ^E^BSSMO_NH2_ showing the longest λ2 (323.4 nm) and ^E^BSSMO_NO2_ the shortest (307.49 nm). (ii) Enol emission (λ_ems_, nm): ^*E^BSSMO_NH2_ exhibited the longest λ1 emission (638.21 nm), while ^*E^BSSMO_NO2_ showed the shortest (366.09 nm). ^*E^BSSMO_CF3_ exhibited the strongest *f*_0_ (0.434), and λ2 transitions showed high *f*_0_ values (^*E^BSSMO_CH3_ = 1.029, ^*E^BSSMO_OH_ = 0.983), indicating efficient secondary emissions. (iii) Keto emission (λ_ems_, nm): the λ1 emissions for the keto form ranged from ^*K^BSSMO_NH2_ (679.57 nm) to ^*K^BSSMO_NO2_ (516.34 nm), with ^*K^BSSMO_NH2_ showing the largest red shift. It was moderate (≈0.2–0.3), with EWGs like ^*K^BSSMO_NO2_ and ^*K^BSSMO_CF3_ exhibiting moderate-to-strong emission intensities. λ2 transitions were consistent (340–356 nm), with ^*K^BSSMO_CH3_ showing the highest *f*_0_ (0.861). In toluene (ε = 2.4), the enol and keto forms exhibited distinct spectral behavior (SI, Table S9): (i) enol absorption (λ_abs_, nm): ^E^BSSMO_NH2_ showed the longest λ1 (451.53 nm), while ^E^BSSMO_CF3_ exhibited the shortest (346.51 nm). Oscillator strengths were higher for electron-withdrawing groups (^E^BSSMO_NO2_, *f*_0_ = 0.728). Higher transitions (λ2λ4) showed subtle trends, with ^E^BSSMO_CF3_ exhibiting relatively high *f*_0_ (0.262) and ^E^BSSMO_OCH3_ having the highest λ4 (277.9 nm). (ii) Enol emission (λ_ems_, nm): the main emission peak ranged from *^E^BSSMO_NH2_ (637.36 nm) to *^E^BSSMO_NO2_ (362.65 nm), with *^E^BSSMO_NH2_ showing the longest emission wavelength. *^E^BSSMO_NO2_ showed weak fluorescence (*f*_0_ = 0.0002), likely due to photoinduced electron transfer (PET). Higher transitions (λ2) exhibited high *f*_0_ values (>0.9) for most substituents. (iii) Keto emission (λ_ems_, nm): NH_2_ showed the longest λ1 emission (678.12 nm), while *^K^BSSMO_NO2_ exhibited the shortest emission (537.92 nm). *f*_0_ values were moderate, with *^K^BSSMO_NH2_ and *^K^BSSMO_NO2_ showing higher *f*_0_ values (∼0.28), indicating strong emission. λ2 transitions (340–384 nm) were dominated by *^K^BSSMO_NH2_ and *^K^BSSMO_CH3_, showing high *f*_0_ values. Besides, enol absorption (λ_abs_, nm) trends in cyclohexane as a low polarity (ε = 2.02) (SI, Table S10): ^E^BSSMO_NH2_, ^E^BSSMO_OCH3_, and ^E^BSSMO_OH_, cause red-shifted absorption maxima (λ1) with ^E^BSSMO_NH2_ exhibiting the longest wavelength (∼450 nm), suggesting enhanced conjugation. In contrast, ^E^BSSMO_CF3_ and ^E^BSSMO_NO2_ induce blue shifts in absorption, with ^E^BSSMO_CF3_ showing the shortest λ1 (∼346 nm). Oscillator strengths (*f*_0_) are highest for EWGs, particularly ^E^BSSMO_CF3_ and ^E^BSSMO_NO2_, indicating strong transition probabilities, despite the shorter absorption wavelengths. Enol emission (λ_ems_, nm) trends in cyclohexane: *^E^BSSMO_NH2_ and *^E^BSSMO_OCH3_ show red-shifted emission wavelengths, with *^E^BSSMO_NH2_ having the longest emission peak (∼635 nm). EWGs such as *^E^BSSMO_CF3_ and *^E^BSSMO_NO2_ result in blue-shifted emissions, with *^E^BSSMO_NO2_ showing the shortest emission wavelength (∼352 nm). The highest fluorescence intensities (*f*_0_) are observed for*^E^BSSMO_CF3_ (0.422) and *^E^BSSMO_H_ (0.309), while *^E^BSSMO_NO2_ exhibits weak emission. Keto emission (λ_ems_, nm) trends in cyclohexane: the keto form displays bathochromic shifts in emission compared to that of the enol form. NH_2_ shows the longest λ1 (∼674 nm), while NO_2_ exhibits the shortest (∼540 nm). Oscillator strengths (*f*_0_) are moderate and relatively consistent across different substituents, with *^K^BSSMO_NH2_ and *^K^BSSMO_NO2_ showing slightly higher values (∼0.28), indicating strong emission for these groups. The λ2 transitions in the keto form display a consistent trend across all substituents, with *^K^BSSMO_NH2_ and *^K^BSSMO_CH3_ exhibiting the strongest *f*_0_ values. The spectra reveal a clear structure–property relationship: EDGs such as NH_2_, OCH_3_ and OH lead to red shifts, enhancing conjugation and dual fluorescence, particularly for NH_2_. Conversely, EWGs such as CF_3_ and NO_2_ result in blue shifts, reducing conjugation but often increasing transition probabilities.

The enol form shows more significant spectral shifts, while the keto form dominates emission in the near-IR/visible range, supporting keto–enol tautomeric dynamics in the excited state. Figure 7 reveals important insights into the absorption and emission behaviors of enol and keto tautomers across different solvents (DMF, DCM, toluene, and cyclohexane) and compared with the gas phase for various substituted compounds. In contrast, strong EWGs like CF_3_ and NO_2_ cause blue shifts, which reflect reduced conjugation, with CF_3_ exhibiting the shortest absorption wavelengths and NO_2_ leading to blue-shifted emissions in both enol and keto forms. Regarding phase dependence, the absorption and emission wavelengths are generally red-shifted in polar solvents such as DMF and DCM compared to the gas phase, with NH_2_-substituted compounds exhibiting the largest shifts.

**Figure 7:**
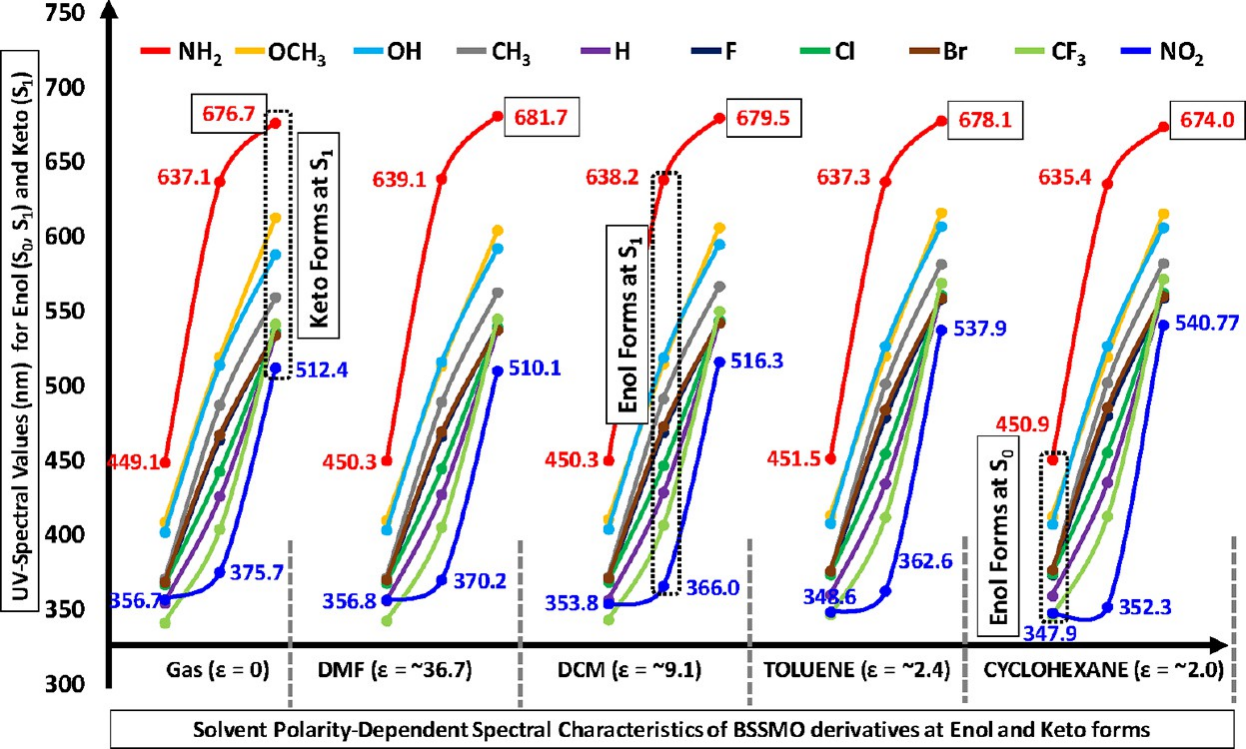
Absorption (enol form) and emission (enol and keto forms) spectra of BSSMO derivatives in various solvents—DMF (ε = 36.7), DCM (ε = 8.9), toluene (ε = 2.4), and cyclohexane (ε ≈ 2.0)—at the B3LYP/6-311+G­(d,p) level and the solvent-dependent spectral shifts and highlight the influence of solvent polarity on electronic transitions.

In nonpolar solvents like toluene and cyclohexane, although the shifts are smaller, NH_2_ still demonstrates significant red shifts, indicating the influence of solvent polarity on the electronic structure and excitation energies. Tautomeric behavior shows that the keto forms typically have longer λ_ems_ than their enol counterparts across all phases, with NH_2_ exhibiting the longest emission wavelengths in both forms. Emission intensities and shifts suggest that the keto form predominates in polar solvents (like DMF and DCM), while the enol form dominates in less polar solvents (like toluene and cyclohexane), with NH_2_ and OCH_3_ compounds showing stronger emissions in nonpolar solvents. Solvent polarity further influences absorption and emission, with increased polarity in DMF and DCM causing bathochromic shifts for most compounds, particularly those with NH_2_ and OCH_3_ substituents. In nonpolar solvents (toluene, cyclohexane), the shifts are less pronounced, but the general trend of red-shifted behavior for EDGs and blue-shifted behavior for EWGs remains. These insights contribute to a deeper understanding of how substituents, solvent polarity, and tautomeric behavior collectively influence the absorption and emission properties of the compounds, offering valuable implications for the design of materials with tunable optical properties for applications in fluorescence-based sensors, light-emitting devices, and other optoelectronic technologies.

To further support the observed solvent-dependent spectral shifts, we have calculated the dipole moments of the studied compounds in the gas phase and in solvents of varying polarity (DMF, DCM, toluene, and cyclohexane) (Table 3). The results reveal that substituents significantly modulate dipole moments, with EDGs such as −NH_2_ and −OCH_3_ showing higher values compared to EWGs. Notably, ^E^BSSMO_NH2_ exhibited the highest dipole moment (6.1128 D in DMF), which correlates with the largest red shifts in both the absorption and emission spectra. In contrast, strongly electron-withdrawing substituent −NO_2_ also showed high dipole moments (6.1120 D in DMF), but due to reduced conjugation, they induced blue shifts in absorption and emission. The general trend indicates that solvent polarity stabilizes molecules with higher dipole moments more effectively, resulting in bathochromic shifts in DMF and DCM, while nonpolar solvents (toluene, cyclohexane) produce smaller shifts.

**Table 3:** Dipole Moment (μ) Values (in Debye) Calculated at the Optimized Ground-State Geometries Using TD-DFT^a^

substituents (R)	gas	DMF	DCM	toluene	cyclohexane
NH_2_	4.6912	6.1128	5.9978	5.6235	5.5418
OCH_3_	2.9721	4.4503	4.3979	4.2218	4.1820
CH_3_	4.2662	5.6791	5.5713	5.2192	5.1421
OH	2.8939	3.7282	3.6630	3.4542	3.4092
H	4.0335	5.0901	4.9928	4.6766	4.6078
F	3.3924	4.1142	4.0454	3.8176	3.7672
Cl	3.2660	4.0031	3.9384	3.7230	3.6750
Br	3.2505	4.0048	3.9401	3.7239	3.6756
CF_3_	3.2420	3.8015	3.7533	3.5898	3.5528
NO_2_	4.4064	6.1121	6.0000	5.6448	5.5689

a Solvents considered include DMF (ε = 36.7), DCM (ε = 8.9), toluene (ε = 2.4), and cyclohexane (ε = 2.02).

### 3.6 Reorganization Energy (λ_RE_)

The electronic properties of the enol and keto of BSSMO derivatives were evaluated by calculating ionization potentials (IPs), electron affinities (EAs), and reorganization energies for both hole (λ_hole_) and electron (λ_electron_) transport and are given in Tables 4 and 5. For the ^E^BSSMO series, the vertical IPs range from 7.06 to 8.11 eV, while the adiabatic IPs fall between 6.71 and 7.86 eV, reflecting the strong electron-donating character of the Schiff base core. Among these, ^E^BSSMO_NH2_ (6.71 eV) exhibits the lowest adiabatic IP, indicating its greater ease of hole injection and better hole-donating ability. In contrast, ^E^BSSMO_NO2_, with the highest IP of 7.86 eV, shows the least favorable hole-injection capability, consistent with the strong electron-withdrawing effect of the nitro group. The electron affinities show a complementary trend, with vertical EAs ranging from −0.72 to −1.28 eV and adiabatic EAs ranging from −1.13 to −1.66 eV. The increase in EA from electron-donating to electron-withdrawing substituents indicates stabilization of the anionic state upon electron addition. ^E^BSSMO_NO2_ (EA­(a) = −1.66 eV) displays the strongest electron-accepting tendency, making it the most promising candidate for electron injection processes, whereas ^E^BSSMO_NH2_ (EA­(a) = −1.13 eV) is relatively less favorable for electron acceptance. The reorganization energies further clarify the charge-transport capabilities. For hole transport, λ_hole_ varies from 0.22 to 0.67 eV. The lowest λ_hole_ value for ^E^BSSMO_Cl_ (0.22 eV) implies excellent hole mobility, while ^E^BSSMO_NH2_ (0.67 eV) may experience a reduced hole transport efficiency. In contrast, the electron reorganization energies (λ_electron_) range from 0.68 to 1.55 eV. ^E^BSSMO_H_ (1.55 eV) shows the highest λ_electron_, indicating weaker electron transport, while ^E^BSSMO_NO2_ (0.68 eV) and ^E^BSSMO_H_ (0.68 eV) demonstrate more favorable electron-transport characteristics. Molecules such as ^E^BSSMO_Cl_ and ^E^BSSMO_Br_, which display a moderate difference between λ_hole_ and λ_electron_, may support balanced ambipolar charge transport—an advantage for optoelectronic applications. Turning to the keto derivatives, the vertical and adiabatic IPs lie within 6.59–7.89 eV and 6.26–7.78 eV, respectively. The lowest adiabatic IP for ^K^BSSMO_NH2_ (6.26 eV) suggests high hole-injection efficiency, while ^K^BSSMO_NO2_ (7.78 eV) again shows the least favorable hole-donating ability due to the strong electron-withdrawing nitro group. The vertical EAs vary from −0.98 to −1.61 eV, and the adiabatic EAs vary from −1.22 to −1.85 eV, indicating that ^K^BSSMO_NO2_ (−1.85 eV) is the strongest electron acceptor and is best suited for electron injection, whereas ^K^BSSMO_NH2_ (−1.22 eV) exhibits the weakest electron affinity. The λ_hole_ values in the KBSSMO derivatives span 0.27–0.57 eV, with ^K^BSSMO_CF3_ (0.27 eV) and ^K^BSSMO_OH_ (0.30 eV) emerging as the most efficient hole-transport materials. For electron transport, λ_electron_ ranges from 0.38–0.68 eV, with ^K^BSSMO_OH_ showing the lowest λ_electron_ (0.38 eV), indicative of efficient electron mobility. Overall, these results emphasize the substituent-dependent tunability of the charge-transport properties in both series. ^E^BSSMO_Cl_ and ^E^BSSMO_Br_ are promising hole-transport candidates due to their low λ_hole_ values.

**Table 4:** Calculated Ionization Potentials (IPs Vertical/Adiabatic in eV), Electron Affinities (EAs (Vertical/Adiabatic) in eV), and Reorganization Energies (λ_hole_ and λ_electron_ in eV) of the Enol Derivatives

molecules	IP(v)	IP(a)	EA(v)	EA(a)	λ_hole_	λ_electron_
^E^BSSMO_NH2_	7.06	6.71	–0.72	–1.13	0.67	0.75
^E^BSSMOO_CH3_	7.27	7.07	–0.82	–1.22	0.41	0.71
^E^BSSMO_OH_	7.37	7.18	–0.82	–1.22	0.38	0.73
^E^BSSMO_CH3_	7.50	7.35	–0.76	–1.17	0.31	0.71
^E^BSSMO_H_	7.65	7.48	–0.79	–1.17	0.33	0.68
^E^BSSMO_F_	7.70	7.51	–0.93	–1.33	0.38	0.73
^E^BSSMO_Cl_	7.70	8.73	–1.03	–1.44	0.22	0.71
^E^BSSMO_Br_	7.67	7.51	–1.03	–1.44	0.33	0.71
^E^BSSMO_CF3_	7.92	7.73	–1.06	–1.47	0.44	0.73
^E^BSSMO_NO2_	8.11	7.86	–1.28	–1.66	0.52	0.68

**Table 5:** Calculated Ionization Potentials (IPs Vertical/Adiabatic in eV), Electron Affinities (EAs (Vertical/Adiabatic) in eV), Reorganization Energies (λ_hole_ and λ_electron_ in eV) of the Keto Derivatives

molecules	IP(v)	IP(a)	EA(v)	EA(a)	λ_hole_	λ_electron_
^K^BSSMO_NH2_	6.59	6.26	–0.98	–1.22	0.57	0.46
^K^BSSMOO_CH3_	6.75	6.50	–1.09	–1.31	0.49	0.46
^K^BSSMO_OH_	6.80	6.61	–1.14	–1.39	0.30	0.38
^K^BSSMO_CH3_	7.05	6.91	–1.03	–1.25	0.30	0.46
^K^BSSMO_H_	7.35	7.07	–1.09	–1.31	0.41	0.44
^K^BSSMO_F_	7.43	7.27	–1.01	–1.25	0.54	0.68
^K^BSSMO_Cl_	7.43	7.29	–1.14	–1.39	0.46	0.63
^K^BSSMO_Br_	7.40	7.27	–1.17	–1.39	0.46	0.60
^K^BSSMO_CF3_	7.54	7.40	–1.39	–1.63	0.27	0.49
^K^BSSMO_NO2_	7.89	7.78	–1.61	–1.85	0.35	0.60

### 3.7 Correlation Analysis between the Hammett Constant (σ) and Computed Properties of Enol and Keto Forms

A correlation analysis between the Hammett constant (σ) and the computed properties was carried out to understand the substituent effects on both the enol and keto forms (SI Figures S6 and S7).

For the enol form, σ values were correlated with *E*_g_, λ_hole_, λ_electron_, and μ. A strong positive correlation was found between σ and *E*_g_, where electron-donating substituents (negative σ, specifically −NH_2_ and −OCH_3_) exhibited smaller gaps (3.23–3.49 eV), while electron-withdrawing groups (positive σ, specifically, −CF_3_ and −NO_2_) showed larger gaps (4.08–4.13 eV), indicating that increasing σ widens *E*_g_ through stabilization of the LUMO. A moderate positive correlation between σ and λ_hole_ revealed that electron-donating groups increased reorganization energy (0.67 eV for −NH_2_), whereas electron-withdrawing groups lowered it (0.22–0.44 eV), implying greater structural relaxation during hole transport for donor substituents. In contrast, λ_electron_ exhibited a weak and nonlinear correlation, with most substituents showing similar values (0.68–0.75 eV), except for the −OH group (1.55 eV), suggesting that hydrogen bonding and resonance effects dominate over inductive behavior. Dipole moment showed a moderately positive correlation with σ, where electron-withdrawing substituents (NO_2_) enhanced molecular polarity by increasing charge separation.

For the keto form, similar correlations were examined. A positive correlation between σ and *E*_g_ indicated that electron-donating groups (−NH_2_, −OCH_3_, and −OH) lowered the energy gap (2.67–2.86 eV), while electron-withdrawing groups (−CF_3_, −NO_2_) increased it (3.24–3.27 eV). The −Br and −Cl derivatives exhibited localized stabilization effects with relatively higher *E*_g_ values (4.63 and 3.06 eV, respectively). A weak correlation between σ and λ_hole_ suggested that donor groups (−NH_2_, −OCH_3_) showed higher values (0.49–0.57 eV), while withdrawing groups (−CF_3_, −NO_2_) displayed lower ones (0.27–0.35 eV), confirming that electron-donating substituents promote greater charge delocalization during hole transport. λ_electron_ showed a moderate positive correlation, increasing from 0.38–0.46 eV for donor groups to 0.60–0.68 eV for strong electron-withdrawing substituents (−F, −NO_2_), reflecting enhanced LUMO stabilization. In contrast, the dipole moment exhibited a negative correlation with σ: donor groups (−NH_2_, −OCH_3_) with higher dipole moments (4.37 and 3.85 D), while electron-withdrawing substituents (−Cl, −Br, −CF_3_) showed much lower values (1.9–1.93 D). In addition, analysis of IP and EA values further supports these substituent effects. For the enol form, the vertical IP (7.06–8.11 eV) and adiabatic IP (6.71–7.86 eV) increased progressively with σ, confirming that electron-withdrawing groups enhance ionization resistance. Conversely, the vertical EA (−0.72 to −1.28 eV) and adiabatic EA (−1.13 to −1.66 eV) became more negative with increasing σ, indicating stronger electron-accepting character for substituents such as −CF_3_ and −NO_2_. Similarly, in the keto form, IP­(v) and IP­(a) values (6.59–7.89 eV and 6.26–7.78 eV, respectively) increased systematically from the donor to acceptor groups, while EA­(v) and EA­(a) (−0.98 to −1.61 eV and −1.22 to −1.85 eV, respectively) showed greater negativity for electron-withdrawing substituents. Hence, donor groups facilitate charge release with lower IP, whereas acceptor groups accept electrons having a higher EA. Overall, these results confirm that the Hammett constant effectively explains how electron-donating and electron-withdrawing substituents modulate the electronic, charge-transport, and polarity characteristics of both the enol and keto forms.

### 3.8 Molecular Docking Analysis

Molecular docking studies were performed to predict and optimize the binding of BSSMO derivatives to PPAR−γ (HAP), an anticancer target that regulates cell proliferation, apoptosis, and differentiation. These studies provide insights for the rational design of potent derivatives with enhanced therapeutic potential. The results indicate that the keto tautomers generally exhibit stronger binding than the enol forms, due to increased polarity and planarity of the carbonyl group facilitating hydrogen bonding and dipole–dipole interactions within the receptor’s active site.^95,96^ Molecular docking studies enable the prediction, optimization, and enhancement of the binding of BSSMO derivatives to their target, providing key insights for the rational design of new, more potent compounds with improved therapeutic potential.^31,97,98^ Furthermore, docking studies revealed that the keto form of BSSMO derivatives generally exhibits enhanced binding affinities compared with their enol counterparts. All enol derivatives displayed moderate binding affinities, with glide scores ranging from −4.27 to −7.42 (Figure 8). While less potent than their keto counterparts, several enol forms still showed favorable interactions with the receptor. Among the enol derivatives, ^E^BSSMO_NO2_ exhibited the strongest binding, with the most negative glide score (−7.42) and the lowest glide energy (−74.00 kcal/mol), indicating highly favorable electrostatic interactions likely driven by the polar hydroxyl group in the enol form. ^E^BSSMO_CF3_ and ^E^BSSMO_F_ also showed relatively strong binding, with glide scores of −6.52 and −6.40 and glide energies of −66.43 and −66.23 kcal/mol, respectively. EWGs such as NO_2_, CF_3_, and F enhanced binding in the enol form, likely by increasing the polarity of the hydroxyl group, promoting hydrogen bonding within the receptor’s active site. On the other hand, enol derivatives with EDGs like NH_2_, OCH_3_, and CH_3_ showed weaker binding affinities, with glide scores above −6.0 and less favorable glide energies (CH_3_: −4.27, −56.44 kcal/mol).

**Figure 8:**
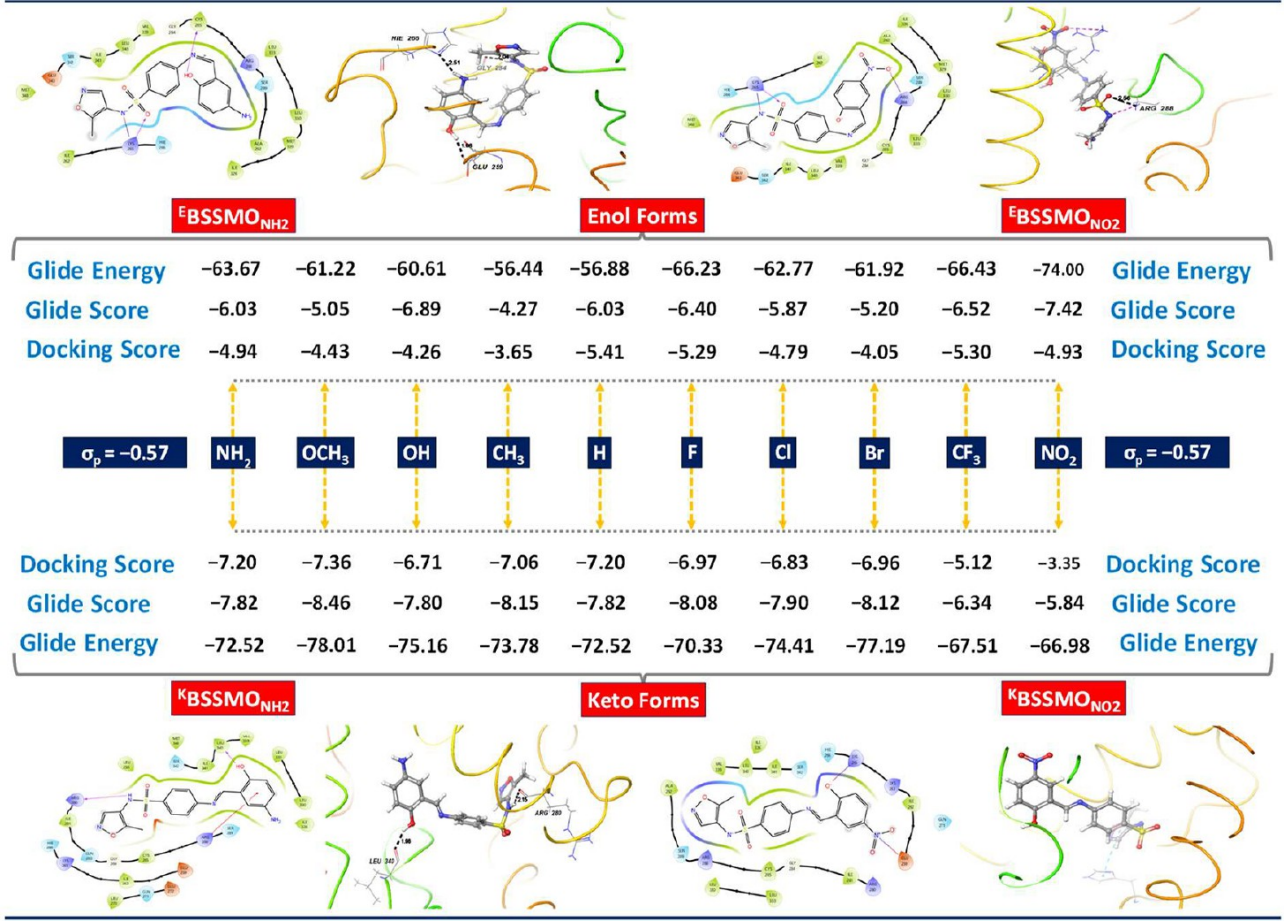
Docking score, glide score and glide energy for the enol and keto forms of BSSMO derivatives with various substituents (R = NH_2_, OCH_3_, OH, CH_3_, H, F, Cl, Br, CF_3_, and NO_2_), showcasing the influence of substituent groups on binding affinity and molecular interactions in the docking simulations.

This suggests that EDGs in the enol form may reduce interaction strength, possibly due to intramolecular hydrogen bonding that limits the external binding potential. Substituents like H, Cl, and Br exhibited intermediate docking scores, indicating moderate interaction potential, likely via van der Waals or nonspecific interactions. In conclusion, while the enol form is generally less favorable than the keto form for receptor binding, EWGs such as NO_2_ and CF_3_ significantly enhance binding affinity in the enol state by increasing polarity and hydrogen-bonding capability. This is evident from more negative docking scores, glide scores, and glide energies, such as those observed for ^K^BSSMO_NH2_ (−7.20, −7.82, and −72.52) and ^K^BSSMO_OCH3_ (−7.36, −8.46, and −78.01). The improved performance of the keto tautomers can be attributed to the increased polarity and planarity of the carbonyl group, which facilitates stronger hydrogen bonding and dipole–dipole interactions within the receptor’s active site. Among all substituents, OCH_3_ emerged as the most promising group, with the lowest glide score (−8.46) and glide energy (−78.01 kcal/mol) in the keto form, indicating an optimal molecular orientation and strong stabilization within the binding pocket. CH_3_ and Br derivatives also displayed strong binding, with glide scores of −8.15 and −8.12 and glide energies of −73.78 and −77.19 kcal/mol, respectively.

In combination, EDGs such as NH_2_, OCH_3_, OH, and CH_3_ greatly enhance docking performance in keto forms. These groups stabilize the keto form via resonance effects and enable favorable electrostatic and hydrogen-bonding interactions with the receptor. Conversely, EWGs CF_3_ (−6.34, −67.51 kcal/mol) and NO_2_ (−5.84, −66.98 kcal/mol) exhibited weaker binding affinities likely due to steric hindrance or reduced electronic compatibility with the active site. Interestingly, enol forms bearing EWGs, such as ^E^BSSMO_NO2_, still demonstrated moderately strong binding (glide energy = −74.00 kcal/mol), possibly due to increased hydroxyl polarity enhancing hydrogen bonding. However, enol tautomers, in general, showed reduced binding relative to keto forms, which can be attributed to intramolecular hydrogen bonding that limits the accessibility of interactive sites. Hydrophobic substituents such as F, Cl, Br, and CH_3_ contributed moderately to binding through van der Waals and hydrophobic interactions. The docking studies with PPAR−γ (HAP) reveal how keto and enol tautomers of BSSMO derivatives interact with the receptor, with substituents such as OCH_3_, NH_2_, and CH_3_ enhancing the binding affinity. These results suggest that specific derivatives may have potential anticancer activity, providing a mechanistic link between computational predictions and real-world therapeutic targets. These findings suggest that substituent type and tautomeric form critically influence binding affinity with keto derivatives containing electron-donating groups, representing the most promising candidates for receptor engagement due to their superior electronic features and conformational compatibility.

### 3.9 ADMET-Related Physicochemical Properties

The ADMET analysis of BSSMO derivatives revealed distinct trends between the enol and keto forms in terms of drug-likeness and oral bioavailability. Across all derivatives, the keto forms consistently showed slightly improved pharmacokinetic profiles compared to their enol counterparts.^99–102^ Specifically, the number of hydrogen bond donors and acceptors remained constant between tautomers for each substituent, indicating no structural changes in the hydrogen-bonding potential. However, the predicted octanol/water partition coefficient (QPlogPo/w) was higher in the keto forms, suggesting increased lipophilicity and, consequently, enhanced membrane permeability. This rise in lipophilicity translated into higher predicted human oral absorption (% HOA) for the keto forms. For instance, the CF_3_-substituted derivative showed the highest QPlogPo/w values (2.626 for enol and 2.998 for keto) and the greatest %HOA (81.295 and 84.79%, respectively).

Similarly, derivatives with halogens such as -Cl and -Br exhibited favorable oral absorption exceeding 82% in the keto form (Table 6). In contrast, derivatives with strong electron-donating groups like NH_2_ showed lower QPlogPo/w and reduced %HOA, with values around 60–63% for both forms. The NO_2_ derivative, bearing a strong electron-withdrawing group, displayed the lowest %HOA (57.19% enol; 58.48% keto), possibly due to increased polarity affecting membrane permeability. These ADMET predictions are preliminary and are based on computational analyses. Although these predictions offer useful insights into potential drug-likeness and oral absorption, they do not fully account for metabolism, toxicity, or bioavailability in a biological system. Therefore, experimental validation through in vitro and in vivo studies will be necessary to confirm the pharmacokinetic, safety, and therapeutic potential of these BSSMO derivatives. Overall, the keto tautomers appear more promising in terms of oral drug-likeness, owing to their increased lipophilicity and higher predicted human oral absorption, but further experimental studies are warranted to substantiate these predictions.

**Table 6:** ADMET-Related Physicochemical Properties of BSSMO Derivatives, including the Number of Hydrogen Bond Donors (Donor HB) and Acceptors (Acceptor HB), Predicted Octanol/Water Partition Coefficient (QPlogPo/w), and Percentage of Predicted Human Oral Absorption (% HOA)^a^

	enol form of ^E^BSSMO_R_				keto forms of ^K^BSSMO_R_			
R	donor HB	acceptor HB	QPlogPo/w	% human oral absorption	donor HB	acceptor HB	QPlogPo/w	% human oral absorption
NH_2_	3.5	8.75	0.955	60.928	3.5	8.75	1.162	63.533
OCH_3_	2	8.5	1.909	76.340	2	8.5	2.130	79.837
CH_3_	3	8.5	1.130	63.065	3	8.5	1.337	65.891
OH	2	7.75	2.053	77.425	2	7.75	2.335	80.884
H	2	7.75	1.761	75.524	2	7.75	2.045	79.280
F	2	7.75	1.841	75.96	2	7.75	2.275	80.565
Cl	2	7.75	1.975	76.772	2	7.75	2.522	82.092
Br	2	7.75	2.069	74.825	2	7.75	2.597	82.465
CF_3_	2	7.75	2.626	81.295	2	7.75	2.998	84.790
NO_2_	2	8.75	1.143	57.186	2	8.75	1.340	58.479

a These parameters are critical for evaluating drug-likeness and oral bioavailability.

### 3.10 Enol-Keto Bonding Unveiled: QTAIM and NCI Insights

QTAIM provides a rigorous framework to analyze chemical bonding and intermolecular interactions through topological analysis of the electron density, ρ­(*r*). This approach is particularly valuable for distinguishing subtle variations in bond character, especially in systems exhibiting tautomerism,^31,103–105^ such as the enol and keto forms of BSSMO derivatives. In this study, these QTAIM descriptors were employed to systematically investigate the electronic and bonding characteristics of both enol and keto tautomers of BSSMO derivatives with various electron-donating and electron-withdrawing substituents. Through comparative analysis of ρ­(*r*), *L*(*r*), *G*(*r*), *V*(*r*), and λ-values across both forms, the influence of substitution on bond strength, polarity, and hydrogen bonding behavior is elucidated, offering deeper insights into the tautomeric preferences and stabilization mechanisms in these molecular systems (SI, Tables S11 and S12, Figures 9, S8, and S9). In the enol form of BSSMO derivatives, the O–H bond exhibits strong intramolecular hydrogen bonding, as indicated by high electron density at the bond critical point (ρ­(*r*) = 0.308–0.316 au), with EDGs such as −OCH_3_, −COOCH_3_, and −OH further enhancing ρ­(*r*) values (∼0.314–0.316 au). These interactions are characterized by positive Laplacians (*L*(*r*) ≈ 0.390–0.404 au), suggesting a polar covalent nature, while increasingly negative potential energy densities (*V*(*r*)) for EDG-substituted derivatives reflect greater stabilization. Conversely, EWGs exhibit slightly lower ρ­(*r*), indicating a weakened interaction strength. In the keto form, the O–H interaction is noncovalent, evidenced by substantially reduced ρ­(*r*) (0.049–0.051 au). EDG-substituted derivatives such as ^K^BSSMO_OCH3_ and ^K^BSSMO_H_ display marginally higher ρ­(*r*) (∼0.0512 au), while EWGs maintain moderate values (∼0.0503–0.0505 au). Negative Laplacians confirm weak shared-shell character consistent with hydrogen bonding. EDGs result in slightly diminished ρ­(*r*), implying weaker interactions in the keto form. The N–H interaction in the enol form is weak, with ρ­(*r*) = 0.045–0.049 au and negative Laplacians, suggesting limited covalent character. ^K^BSSMO_NO2_ and ^K^BSSMO_NH2_ show relatively higher ρ­(*r*), potentially due to resonance stabilization.

**Figure 9:**
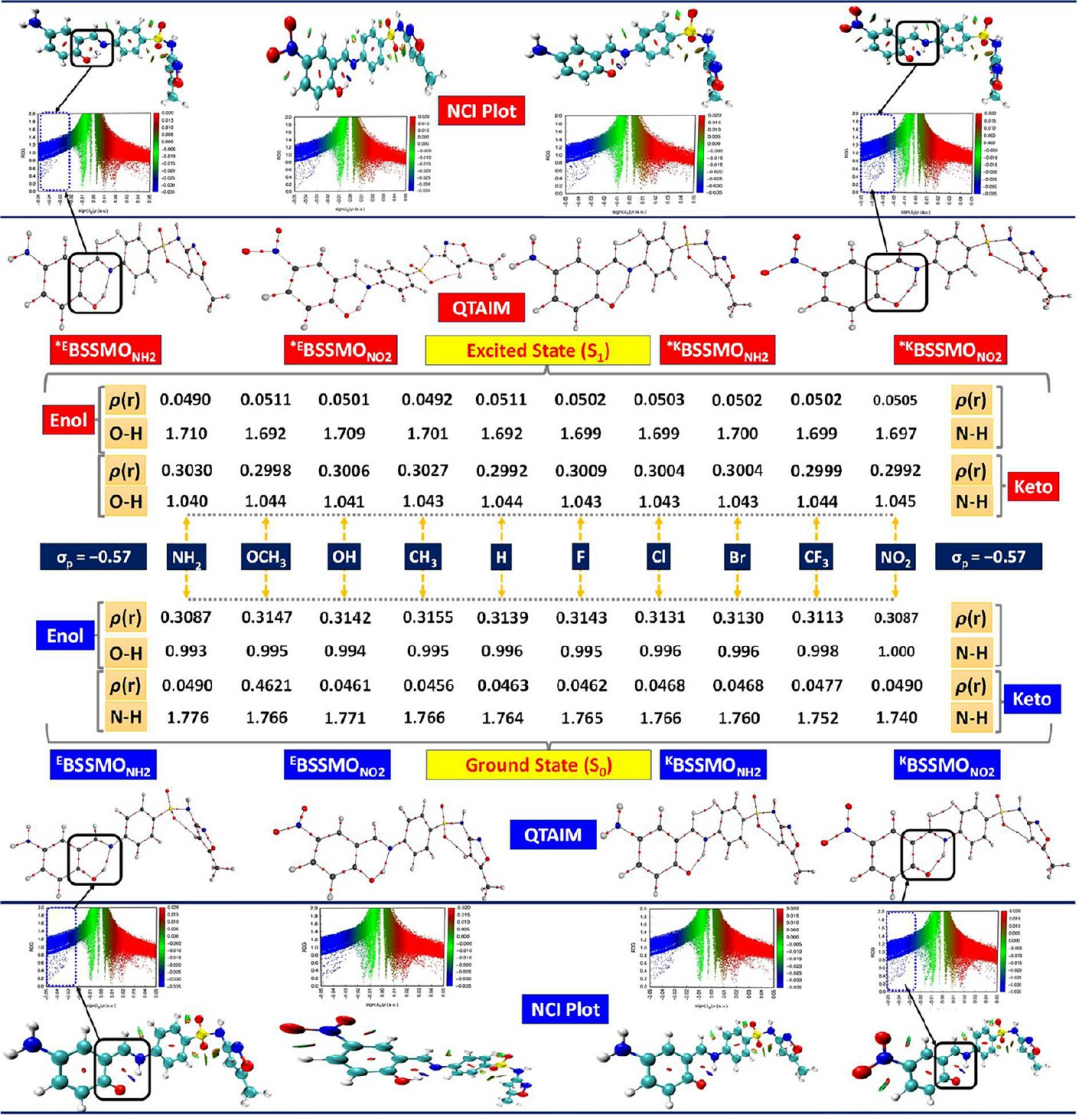
QTAIM and NCI (RDG isosurface maps and scatter plots) analyses of selected O–H and N–H bond lengths (in Å) and their interaction strengths for ^E^BSSMO_NH2_ to ^E^BSSMO_NO2_ derivatives in the S_0_ and S_1_ states.

In the keto form, the N–H bond exhibits a clear covalent character, as reflected by elevated ρ­(*r*) (∼0.299–0.303 au), positive Laplacians (*L*(*r*) ≈ 0.365–0.373 au), and high *V*(*r*) values (≈0.449–0.457 au). Minimal variation in *G*(*r*) and λ-values across derivatives indicates that substitution exerts little influence on the topological features of the N–H bond in the keto tautomer. Comprehensive, the QTAIM results highlight distinct patterns in bonding behavior across enol and keto forms. EDGs (CH_3_, OH, and OCH_3_) enhance electron density and stabilize the O–H bond in the enol form, leading to stronger hydrogen bonding and shorter bond lengths. Conversely, EWGs (NO_2_, CF_3_) tend to reduce ρ­(*r*) and weaken the bond, as shown by longer bond distances and less negative *V*(*r*) values. While the N–H bond remains relatively weak and constant in enol forms, it becomes significantly stronger and more covalent in keto forms, especially in the presence of EDGs. The O–H bond in keto forms exhibits moderate hydrogen bonding that is relatively stable across substitutions. Derivatives with OCH_3_ and H substituents show the most stabilized hydrogen bonding, while NH_2_ and CH_3_ derivatives promote stronger covalent character in the N–H bond. These trends underscore the substituent-dependent redistribution of electron density and the bonding nature during tautomerization in BSSMO derivatives.

To investigate the influence of weak intramolecular forces on the structural and electronic properties of the BSSMO derivatives, noncovalent interaction (NCI) analysis was carried out for both the enol (E) and keto (K) forms.^83,106^ The keto form corresponds to the tautomer bearing a carbonyl group (C=O) at the former phenolic position, stabilized by intramolecular hydrogen bonding with azomethine nitrogen. NCI visualizations, including isosurfaces mapped over the reduced density gradient (RDG) and RDG vs sign­(λ)­ρ scatter plots, clearly delineate regions of noncovalent interactions. Blue isosurfaces, representative of strong, attractive intramolecular hydrogen bonding, were most prominent in the −OH and −OCH_3_-substituted keto derivatives, with the −OH keto form exhibiting the most favorable interaction due to optimal hydrogen bond geometry and donor–acceptor alignment. Green isosurfaces, indicative of van der Waals interactions, were present in all derivatives but were particularly extensive in halogenated and alkyl-substituted species (−CH_3_, −Br, and −CF_3_), suggesting dispersion-dominated stabilization. In contrast, red isosurfaces—associated with steric repulsion and unfavorable lone pair–lone pair interactions—were evident in electronegative substituents (−F, −Cl, −Br, and −CF_3_).

Overall, the keto tautomers, especially the −OH-substituted form, displayed more localized and directionally favorable noncovalent interactions than their enol counterparts, indicating enhanced conformational stability driven by intramolecular hydrogen bonding. Inclusive QTAIM and NCI analyses reveal that the electronic and structural features of BSSMO derivatives are highly substituent-dependent. Electron-donating groups (−CH_3_, −OH, and −OCH_3_) enhance electron density and strengthen covalent and hydrogen bonding, especially in the enol form, while electron-withdrawing groups (−NO_2_, −CF_3_) weaken these interactions. The O–H bond is strong and covalent in enol forms but becomes longer and noncovalent in keto forms. In contrast, N–H bonds are weak in enol forms but gain covalent character in keto forms, particularly with EDGs. Strong intramolecular hydrogen bonding in −OH and −OCH_3_ keto derivatives, supported by topological and RDG features, highlights the −OH keto tautomer as the most stabilized structure (Figures 9 and S10–S13 (SI)).

### 3.11 MESP Analysis of Enol and Keto Forms of BSSMO Derivatives

The molecular electrostatic potential (MESP) maps^107,108^ of the enol and keto forms of BSSMO derivatives reveal distinctly different electrostatic environments, which are strongly governed by the nature of the substituents attached to the aromatic ring. This analysis effectively highlights regions prone to electrophilic and nucleophilic attack, offering detailed insights into the charge distribution and intramolecular interactions across the molecules. In the enol form, the O–H functional group consistently appears as a region of high negative electrostatic potential, typically visualized as intense red zones on the MESP surface (refer to Figure 10). This indicates strong electron density around the hydroxyl oxygen, which facilitates intramolecular hydrogen bonding. The effect of substituents on this electrostatic potential is pronounced.^109,110^ EDGs enhance the negative potential around the O–H group, intensifying the red color in these regions and increasing the molecule’s nucleophilic character. These changes promote stronger intramolecular hydrogen bonding and overall stabilization of the enol tautomer. On the other hand, EWGs diminish the negative potential on the hydroxyl oxygen, often shifting the electrostatic regions to more positive values, visualized as yellow to green zones. This indicates a reduction in electron density and weakening of the hydrogen-bonding capability. The strong electron-withdrawing nature of NO_2_ and CF_3_ induces highly polarized electrostatic surfaces, marked by distinct electron-rich (red) and electron-poor (blue) regions. Moderately withdrawing groups such as Br and Cl produce less polarized MESP patterns, with reduced charge delocalization compared to EDGs. Enol forms bearing EDGs display more uniform negative potential distributions and stronger hydrogen bonding, while EWGs exhibit localized positive potentials and diminished electron density at reactive sites.

**Figure 10:**
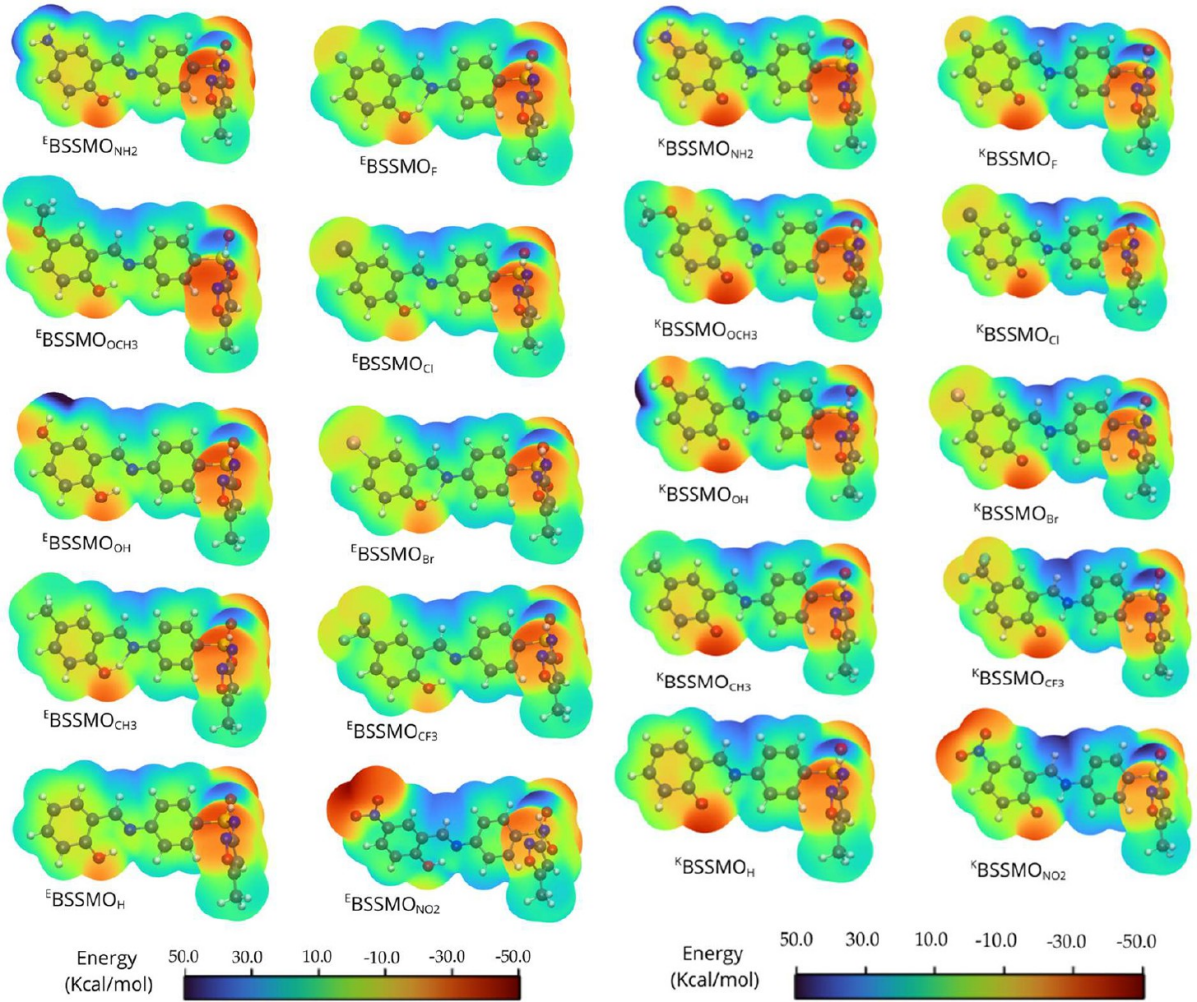
Molecular electrostatic potential (MESP) maps of the enol and keto forms of BSSMO derivatives with various substituents (R = NH_2_, OCH_3_, OH, CH_3_, H, F, Cl, Br, CF_3_, and NO_2_), illustrating the influence of electron-donating and electron-withdrawing groups on electrostatic potential distribution and intramolecular interactions.

In the keto form, the dominant electrostatic feature is the intramolecular N–H···O hydrogen bond, evident as a central interaction zone on the MESP surface. EDGs enhance the negative potential around the carbonyl oxygen, reinforcing the hydrogen bond strength and nucleophilicity through extended red regions. In contrast, electron-withdrawing substituents such as NO_2_ and CF_3_ introduce more positively charged regions, particularly around the aromatic ring and near the substituent site (Figure 10). However, these groups can also slightly increase the electrostatic potential near the N–H group, reflecting their ability to polarize the electron distribution across the molecule. Overall, keto forms present more diffuse electrostatic surfaces compared to enol forms, owing to the lack of a free hydroxyl group and the formation of a conjugated carbonyl system. Among all derivatives, the H and OCH_3_-substituted systems demonstrate a more balanced potential distribution, suggesting favorable stabilization in both tautomeric states. MESP analysis reveals that electron-donating groups enhance negative electrostatic potential at hydrogen-bonding sites, strengthening intramolecular interactions and stabilizing both enol and keto forms. In contrast, electron-withdrawing groups shift the potential positively, weakening hydrogen bonds but increasing molecular polarity. These trends directly influence the stability, reactivity, and tautomeric balance of BSSMO derivatives, underscoring the importance of substitution patterns in modulating their physicochemical properties.

### 3.12 Molecular Dynamics Investigation of −NH_2_ and −NO_2_-Substituted ^E^BSSMO

The ^E^BSSMO_NH2_ and ^E^BSSMO_NO2_ ligands analyzed in these molecular dynamics (MD) simulations include derivatives bearing either an amino (−NH_2_) or nitro (−NO_2_) substituent on the aromatic ring (refer to Figure 11). The NH_2_ group is identified by the presence of a nitrogen atom attached to the aromatic ring, capable of participating in hydrogen bonding as a donor due to its lone pair and associated hydrogen atoms.^111^ Conversely, the NO_2_ group consists of a nitrogen atom double-bonded to one oxygen and single-bonded to another negatively charged oxygen, exhibiting a delocalized electronic structure that renders the oxygens effective hydrogen bond acceptors and contributes to the ligands’ overall negative charge (−1). To investigate the behavior of these substituents during the 100.102 ns MD simulation, several components of the trajectory analysis must be considered. The Protein–Ligand Contacts section provides a detailed schematic and timeline of interactions such as hydrogen bonds, hydrophobic contacts, ionic interactions, and water bridges.^112^ For the NH_2_ group, the focus should be on hydrogen bonds involving the amino nitrogen, examining whether it acts as a donor to protein acceptor atoms. For the NO_2_ group, the negatively charged oxygens may accept hydrogen bonds or engage in ionic interactions with positively charged residues within 3.7 Å. Water-mediated bridges are also relevant for both groups, especially if the direct contacts are sterically hindered.

**Figure 11:**
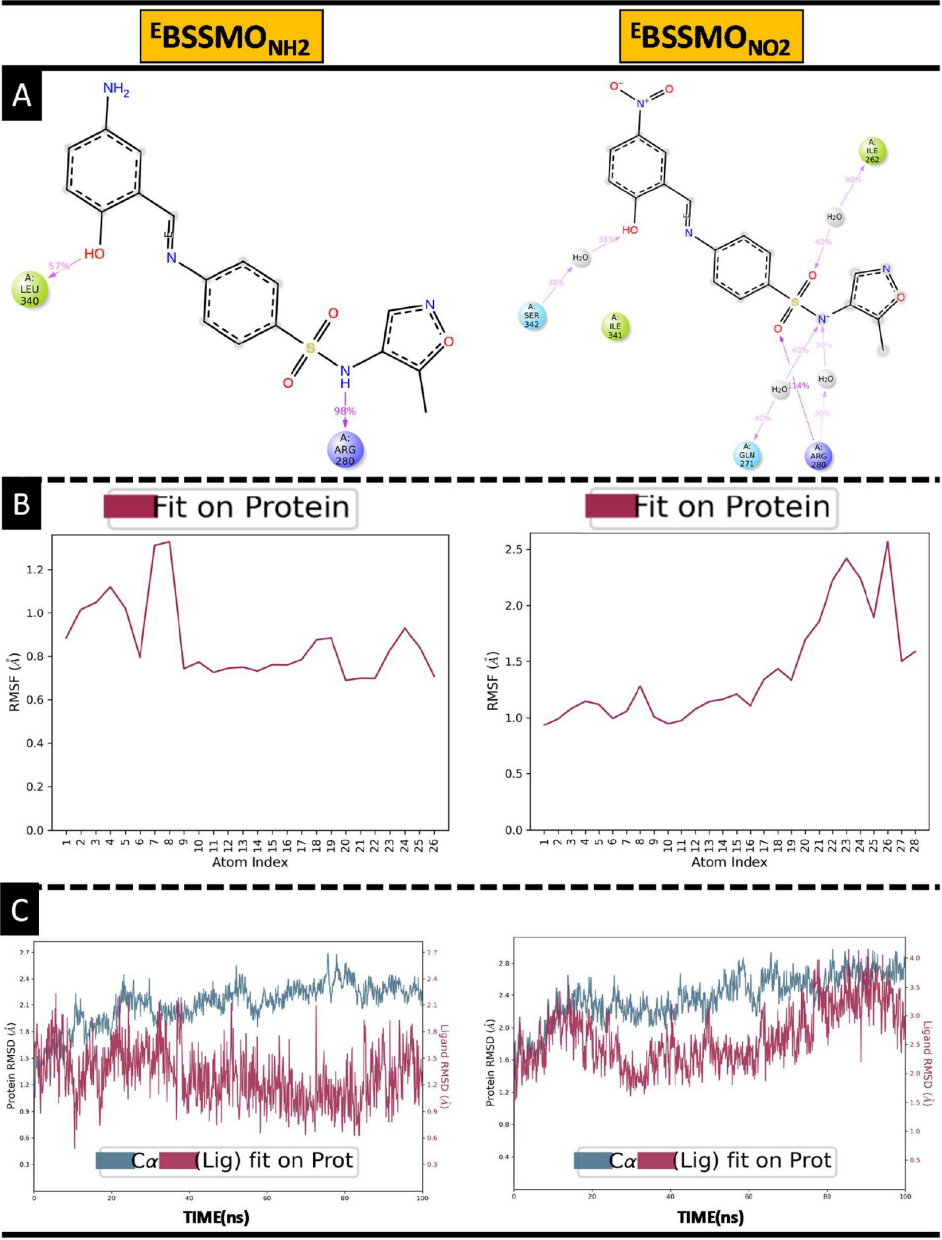
2D binding interactions of ^E^BSSMO_NH2_ and ^E^BSSMO_NO2_ with the target protein (A), ligand RMSF plots during 100 ns simulations (B), and protein and ligand RMSD plots over 100 ns (C).

Interactions persisting for over 30% of the simulation time are especially informative, reflecting potentially stable binding contributions. The ligand root-mean-square fluctuation (RMSF) plot (refer to Figure 11) provides insight into atomic flexibility, highlighting the dynamic behavior of individual atoms throughout the simulation. Monitoring the NH_2_ group or the NO_2_ group can reveal their relative mobility and potential solvent exposure. The “Fit Ligand on Protein” line within the RMSF analysis, which aligns the ligand to the protein structure, helps distinguish intrinsic ligand flexibility from movement relative to the binding pocket.^113^ In addition, the Ligand Torsion Profile is useful if the bonds connecting either substituent to the aromatic ring are defined as rotatable in the simulation. Dial plots and bar graphs capture the range and distribution of torsional angles sampled over time, reflecting the conformational preferences of these groups.

Potential energy profiles, if available, indicate whether certain orientations are energetically favored or disfavored within the protein environment—information particularly relevant if steric hindrance or electronic effects influence substituent conformation. Finally, the Ligand Properties section, especially the Polar Surface Area (PSA), captures the contribution of polar functional groups to the overall ligand polarity. The NO_2_ group, in particular, significantly increases PSA and enhances the ligand’s potential for polar interactions, whereas the NH_2_ group contributes both to polarity and hydrogen-bonding capability. In conclusion, a thorough understanding of the behavior of the −NH_2_ and −NO_2_ substituents during MD simulation requires integrated evaluation of protein–ligand contact persistence, atomic fluctuations *via* RMSF analysis, torsional preferences, and the influence of polarity as reflected in PSA. Among these, the ligand–protein contact schematic is particularly important for visualizing persistent, functionally relevant interactions and understanding each substituent’s role in ligand binding affinity and stability (Figure S14).

### 3.13 Modulation of Energy Gaps and Electronic Spectra of Absorption in Cu­(II)-BSSMO Complexes

Based on Table S13 (SI) of enol absorption (λ_abs_, nm) of the Cu­(II) complex at the gas phase for various substituents (R), key observations can be made regarding the energy gap (*E*_g_) and its correlation with the nature of the substituent groups. The energy gap shows a distinct trend, increasing in the order: NH_2_ (2.16 eV) < OCH_3_/ OH (2.33 eV) < CH_3_ (2.58 eV) < F (2.55 eV) < Cl (2.59 eV) < Br (2.57 eV) < CF_3_ (2.77 eV) < NO_2_ (2.83 eV) (SI Table S4). This trend reveals that electron-withdrawing groups (EWGs) like CF_3_ and NO_2_ result in a higher *E*_g_, while electron-donating groups (EDGs) such as NH_2_, OH, and OCH_3_ tend to lower the energy gap.^114,115^ EDGs contribute to longer wavelength (λ1) transitions, which are red-shifted and indicate lower energy transitions. This is consistent with their effect of stabilizing the HOMO and destabilizing the LUMO, thereby narrowing the *E*_g_. Conversely, EWGs lead to shorter wavelength (λ1) transitions (blue-shifted), which correspond to higher energy transitions and a larger *E*_g_ due to their LUMO-lowering effect. Comparing specific substituents, NH_2_ demonstrates the lowest *E*_g_ (2.16 eV), highlighting its strong donating character, whereas NO_2_ shows the highest *E*_g_ (2.83 eV), confirming its strong withdrawing nature. Halogen substituents such as F, Cl, and Br show intermediate *E*_g_ values (∼2.55–2.59 eV), reflecting a balance between their inductive and resonance effects. The CH_3_ group, with an *E*_g_ of 2.58 eV, displays a slightly lower gap than halogens, aligning with its mild electron-donating nature through hyperconjugation.

Notably, λ1 values decrease systematically from NH_2_ (778.61 nm) to NO_2_ (543.14 nm), supporting the inverse correlation between λ and *E*_g_ (since λ_max_ ∝ 1/*E*_g_) and further affirming the electronic effects of the substituents. While the *f*_0_ values are relatively low for transitions λ1 and λ2, they are significantly higher for λ3 and λ4, suggesting that these latter transitions are more allowed and likely of π→π* character. Although the substituent influence on *f*_0_ is evident, it is less systematic compared to their effects on *E*_g_ and λ1. Mostly, the substitution pattern plays a critical role in modulating the optical absorption and electronic properties of the Cu-BSSMO complex.^107–120^ The energy gap variation with different substituents underscores the potential of fine-tuning the electronic properties via substituent engineering, which is of great relevance for applications in molecular electronics, photophysics, and catalytic systems.

## 4 Conclusions

In comparison with prior studies, this work provides substituent-specific insights into ESIPT and GSIPT in BSSMO derivatives. Our results reveal how EDGs and EWGs differently modulate frontier orbital energies, optical properties, and binding affinities, highlighting structure–property relationships not previously reported. By combining DFT/TD-DFT calculations, transition state analysis, and molecular docking, we identify promising candidates—particularly keto tautomers with OCH_3_, NH_2_, or CH_3_ substituents—for optoelectronic and anticancer applications, thereby extending the technological relevance of previous studies. In this study, a detailed investigation of 10 BSSMO derivatives, functionalized with a range of EDGs and EWGs, aimed at elucidating their influence on GSIPT and ESIPT processes as well as on associated physicochemical and pharmacokinetic properties. The results demonstrate a well-defined structure–property relationship, wherein both EDGs and EWGs markedly alter the electronic distribution, molecular geometry, hydrogen-bonding patterns, vibrational characteristics, optical transitions, and binding affinities of the BSSMO molecules. EWGs were generally found to enhance intramolecular hydrogen bonding in both enol and keto tautomers, as reflected in the elongation of O–H and N–H bonds and the shortening of C=O bonds, whereas EDGs exhibited the opposite trend. Vibrational (IR) analysis further supported this observation, with EDGs leading to stronger hydrogen bonds and higher O–H stretching frequencies, while EWGs introduced more flexibility. FMO analysis revealed that EDGs raise the HOMO energy levels and narrow the *E*_g_, indicating greater reactivity, while EWGs increase this gap, implying enhanced stability. Photophysical analysis showed that EDGs induce bathochromic shifts in both absorption and emission spectra, while EWGs cause hypsochromic shifts; these effects are further modulated by solvent polarity, with polar solvents generally causing red shifts. Molecular docking studies revealed that keto tautomers tend to bind more strongly to receptors than enol forms, with EDGs containing keto derivatives displaying enhanced binding affinities, while EWG-containing molecules typically showed weaker interactions, though some EWG-bearing enol derivatives exhibited moderate binding. Additionally, keto forms generally demonstrated improved ADMET profiles, such as higher lipophilicity and better predicted oral absorption. QTAIM analysis showed that EDGs increase electron density and stabilize the O–H bond in the enol form, while EWGs weaken it; the N–H bond in keto forms was found to be stronger and more covalent, particularly with EDG substitution. MESP maps supported these findings, revealing that EDGs increased negative potential in hydrogen-bonding regions, enhancing stability, whereas EWGs shifted the potential toward positive values, decreasing the hydrogen bond strength but increasing molecular polarity. Finally, MD simulations of −NH_2_ and −NO_2_-substituted ligands highlighted the distinct functional roles of these groups in protein–ligand interactions: −NH_2_ acted predominantly as a hydrogen bond donor, while −NO_2_ functioned as a hydrogen bond acceptor or engaged in ionic interactions. Comprehensively, the substitution pattern plays a pivotal role in modulating the optical absorption and electronic properties of the Cu-BSSMO complex. The observed variation in energy gaps across different substituents highlights the potential of fine-tuning electronic characteristics through strategic substituent engineering, which is particularly valuable for applications in molecular electronics, photophysics, and catalysis. Furthermore, this study emphasizes the utility of rational substitution with EDGs and EWGs to effectively tailor GSIPT and ESIPT behaviors, and also, the correlation analysis clearly demonstrates that EWG substituents (positive σ) increase the energy gap, ionization potential, and electron affinity, thereby enhancing electronic stability and polarity, whereas electron-donating substituents (negative σ) reduce the band gap and reorganization energies, promoting charge delocalization and efficient charge transport. Overall, the Hammett constant (σ) effectively rationalizes the substituent-dependent modulation of electronic, charge transfer, and dipolar characteristics in both enol and keto forms. Such tuning enables optimization of spectroscopic and electronic properties, as well as enhancements in receptor binding affinity and drug-likeness. Notably, keto tautomers bearing EDG substitutions emerge as especially promising candidates for further exploration in both optoelectronic device development and therapeutic design.

## Supplementary Material



## Notes

Molecular docking analysis of protein preparation, receptor grid generation and ligand preparation; HOMO–LUMO energy gaps for all molecules, computed potential energy landscape energies, calculated electronic properties (absorption and emission wavelengths), and comparison of emission spectrum and docking scores and the correlation diagrams; topological parameters obtained from QTAIM analysis, as well as the calculated electronic properties for Cu­(II) complexes; optimized ground-state geometries, frontier molecular orbitals (FMO) of various substituents for non-ESIPT molecules at both the S_0_ and S_1_ states, correlation diagrams, molecular graphs and electron gradient image from QTAIM analysis, NCI plots and RDG isosurfaces, alongside RMSF plots of the ^E^BSSMO_NH2_ and ^E^BSSMO_NO2_ complexes with the 2HAP protein (PDF)
