# Supplementary material for: Theoretical Insights into ESIPT and GSIPT in Schiff Base Cu(II)-BSSMO Complexes: Substituent Effects Explored via DFT, Molecular Docking, and Dynamics Simulations for Optoelectronic and Biomedical Applications
Source: ACS Omega. 2025 Nov 12;10(46):55628–51. doi: 10.1021/acsomega.5c06772 (PMC12658660; doi:10.1021/acsomega.5c06772)
Supplement: Supplementary file 1 [file ao5c06772_si_001.pdf]

Supporting Information

Theoretical Insights into ESIPT and GSIPT in Schiff Base Cu(II)-BSSMO Complexes: Substituent Effects Explored *via* DFT, Molecular Docking, and Dynamics Simulations for Optoelectronic and Biomedical Applications

Murugesan Panneerselvam,[#1,2]* Anantha Narayanan Sri Gayathri,[#3] Singaravel Nathiya,[1] Jarede Da Silva Martins,[1] Iravatham Rama,[3] Frederico W. Tavares,[2,4]* Luciano T. Costa[1]*

[1]MolMod-CS, Institute of Chemistry, Fluminense Federal University (UFF), CEP: 24020-141, Niterói-RJ, Brazil.
[2]Chemical Engineering Program (PEQ/COPPE), Federal University of Rio de Janeiro (UFRJ), CEP: 21941-594, RJ, Brazil.
[3]PG and Research Department of Chemistry, Seethalakshmi Ramaswami College, Affiliated to Bharathidasan University, Tiruchirappalli 620 002, Tamil Nadu, India.
[4]Chemical and Biochemical Process Engineering, School of Chemistry, Federal University of Rio de Janeiro (UFRJ), CEP:21941-594, RJ, Brazil
[#]*Contributed equally to this work*
Email(s): *panneerchem130491@gmail.com, tavares@eq.ufrj.br & ltcosta@id.uff.br*

Section 1. (Extensive Descriptions of Methods for docking Analysis)

1.0 Protein Preparation

The crystal structure of the PPAR-γ protein (PDB ID: 2HAP) was retrieved from the Protein Data Bank[1], exhibiting a resolution of 1.60 Å. Protein preparation was performed using the Protein Preparation Wizard (PPW) in Maestro, encompassing three stages: structure importation and refinement, review and modification, and energy minimization. Missing side chains and loops were added, hydrogen bonds and disulfide bonds were assigned, and water molecules within 5 Å were removed. The structure was protonated at pH 7.0 using Epik, and energy minimization was carried out using the OPLS4 force field.[2]

1.1 Receptor Grid Generation

A receptor grid was generated using the prepared protein structure, maintaining the co-crystallized ligand to define the binding site. The grid dimensions were set to 10 Å × 12 Å × 14 Å along the X, Y, and Z axes, respectively, ensuring comprehensive coverage of the active site for accurate docking simulations.[3,4]

1.2 Ligand Preparation

The optimized geometries of the Ligands, including the enol and keto forms of BSSMO derivatives, are taken for the preparation. LigPrep was utilized for ligand preparation, with a molecular weight cutoff of 500 Da.[5,6] Ionization states were generated at pH 7.0 using Epik, preserving stereochemistry to maintain accurate 3D configurations for docking.[7,8]

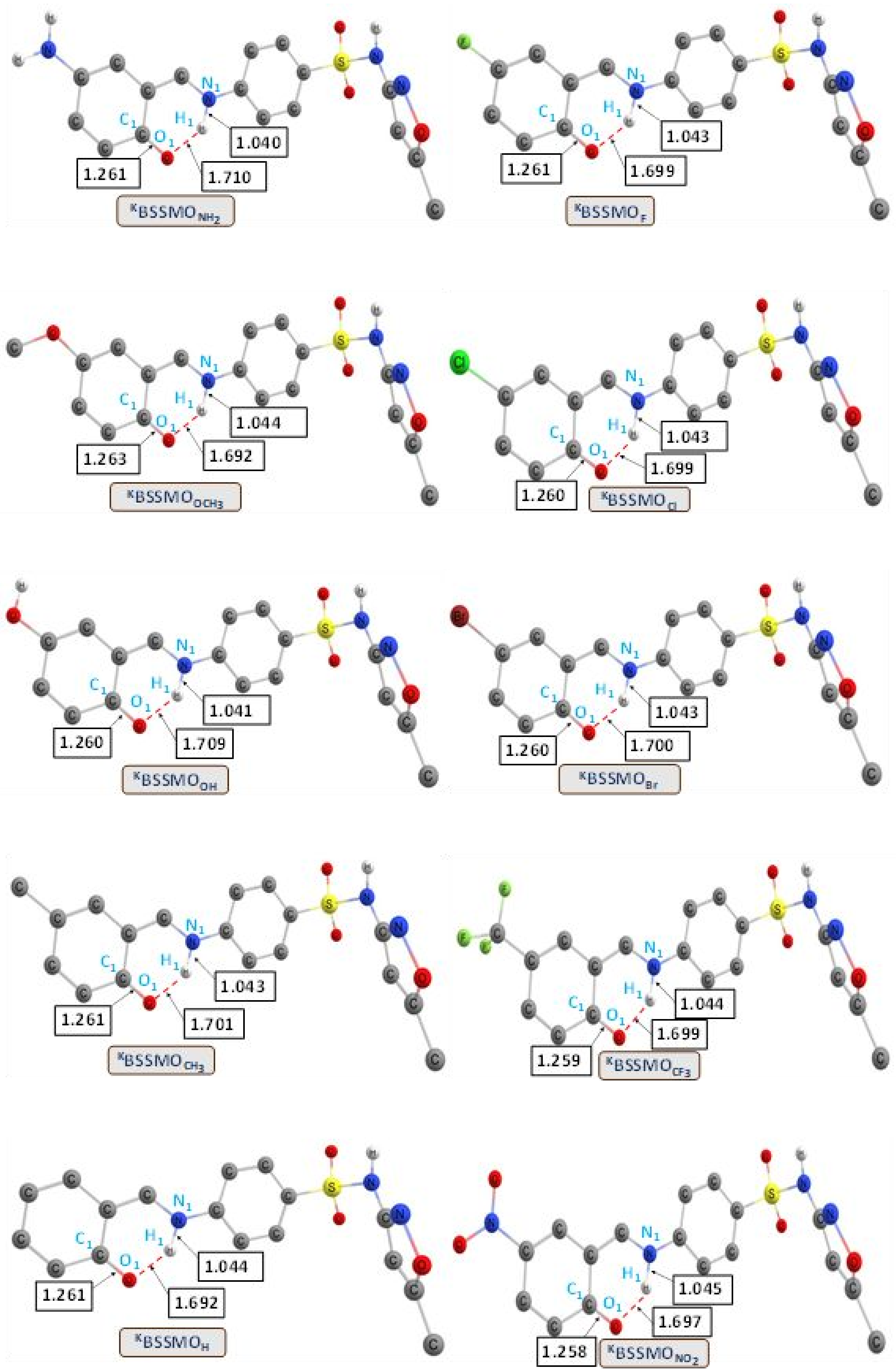

Figure S1. Selected bond lengths for the optimized ground-state geometries of various substituents in their keto ($^{K}BSSMO_{R}$) forms in the gas phase at the S_0 state.

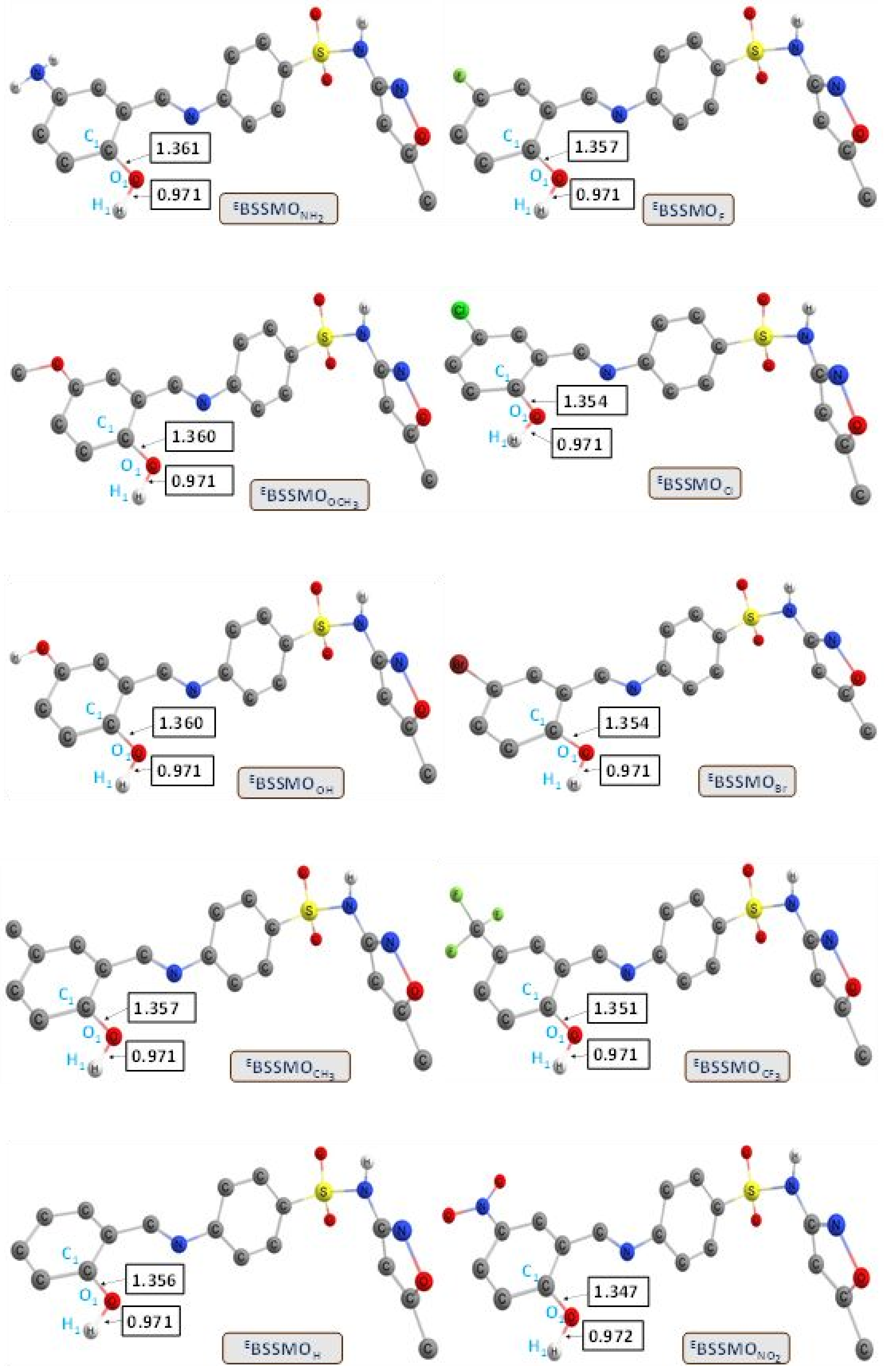

Figure S2. Selected bond lengths for the optimized ground-state geometries of various substituents in their enol ($^{NON\text{-}ESIPT}BSSMO_R$) forms in the gas phase at the S_0 state.

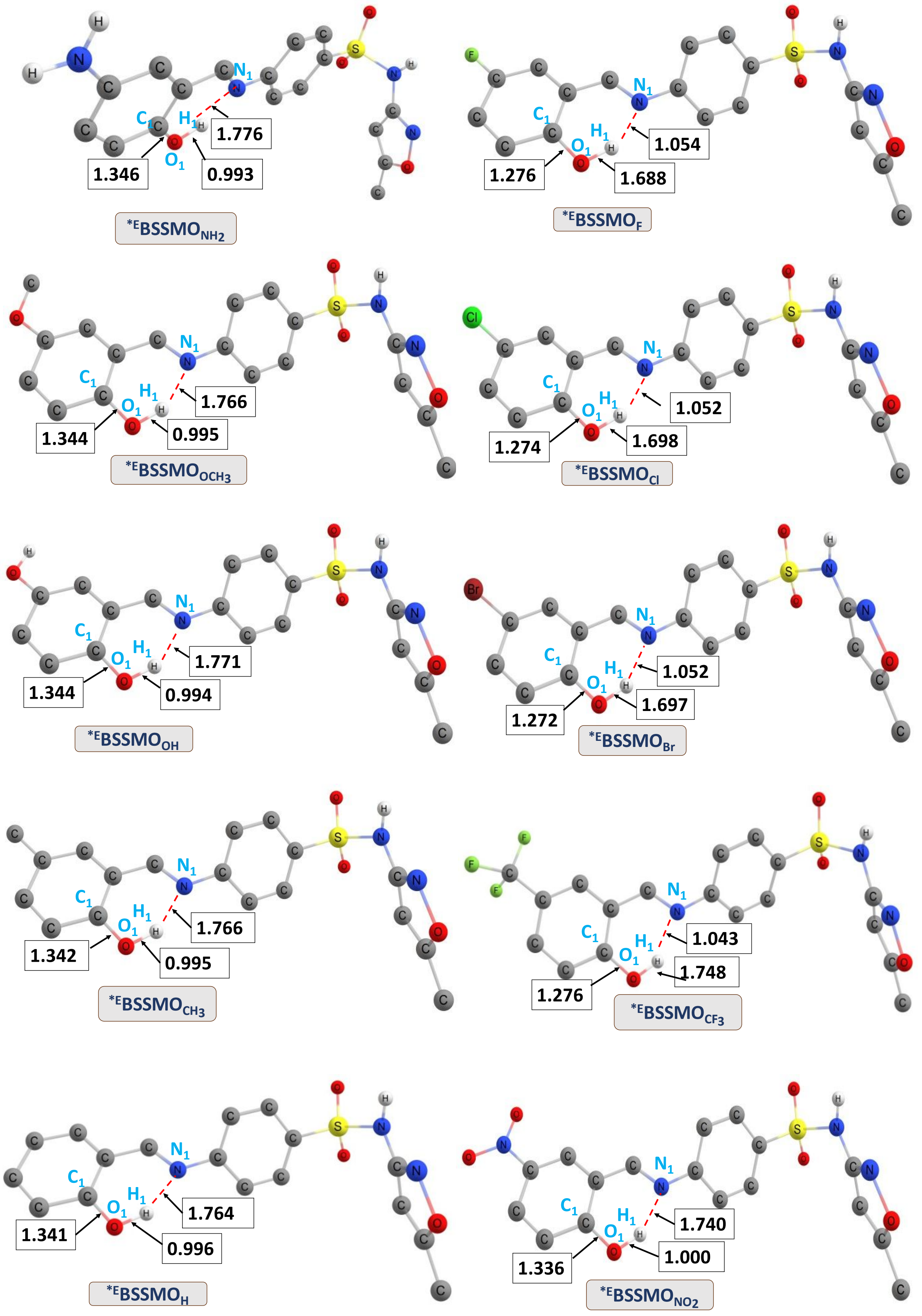

Figure S3. Selected bond lengths for the optimized excited-state geometries of various substituents in their enol (^{*E}BSSMO$_R$) forms in the gas phase at the S_1 state.

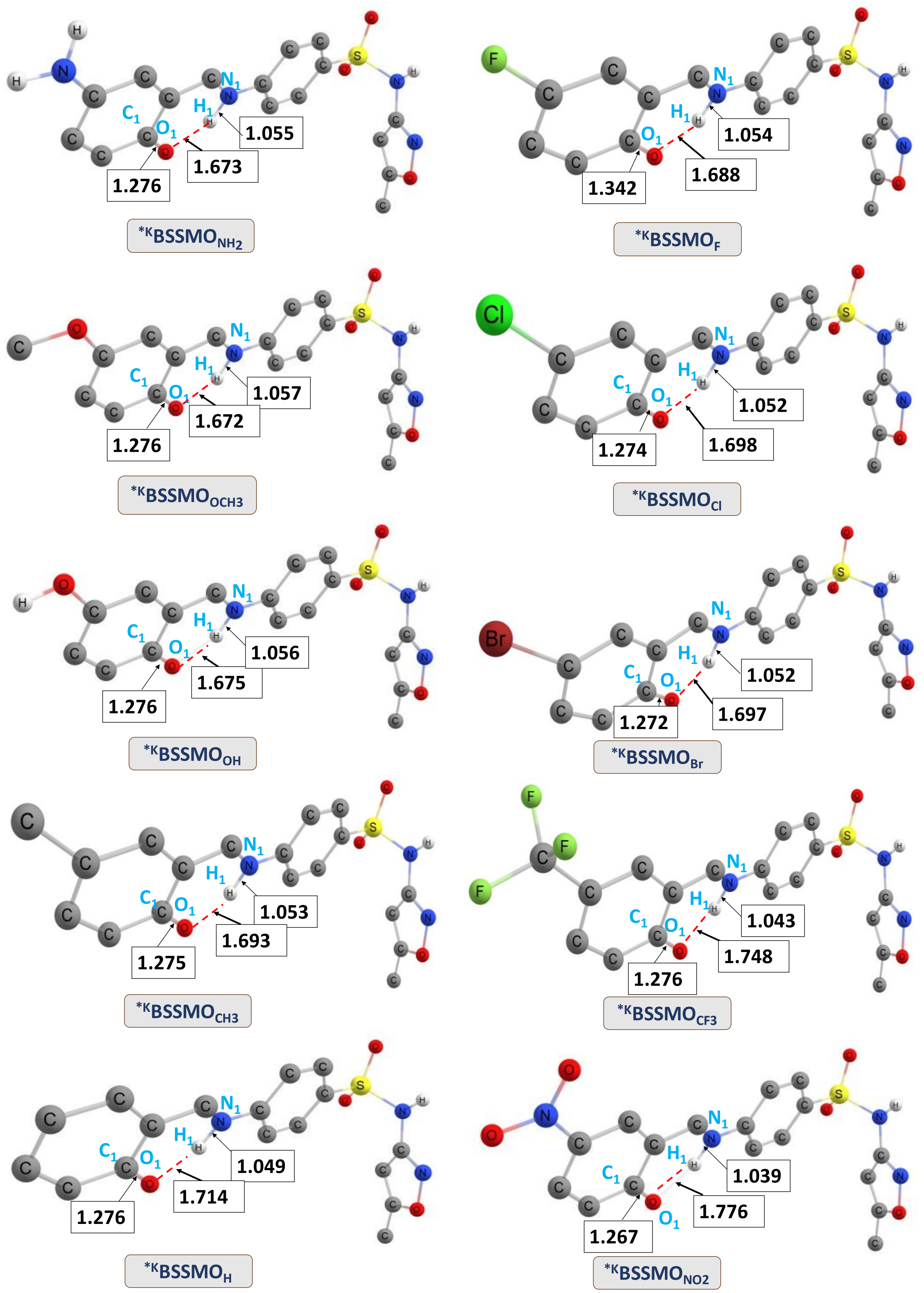

Figure S4. Selected bond lengths for the optimized excited-state geometries of various substituents in their keto ($^{*K}BSSMO_R$) forms in the gas phase at the S_1 state.

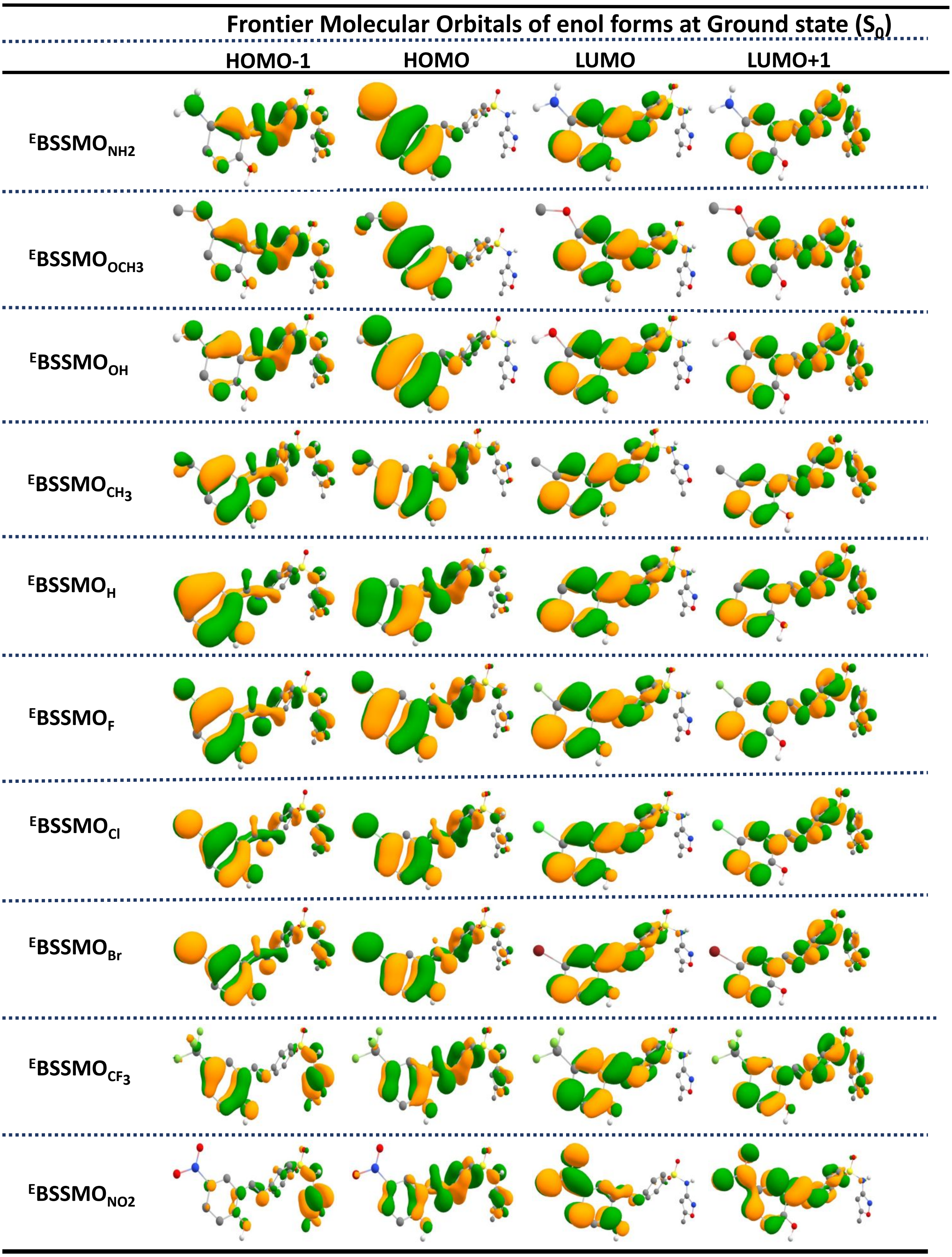

Figure. S5. Frontier molecular orbitals (FMOs) for the enol forms of all substituents ($^{NON\text{-}ESIPT}BSSMO_R$) in the gas phase at the ground state (S_0).

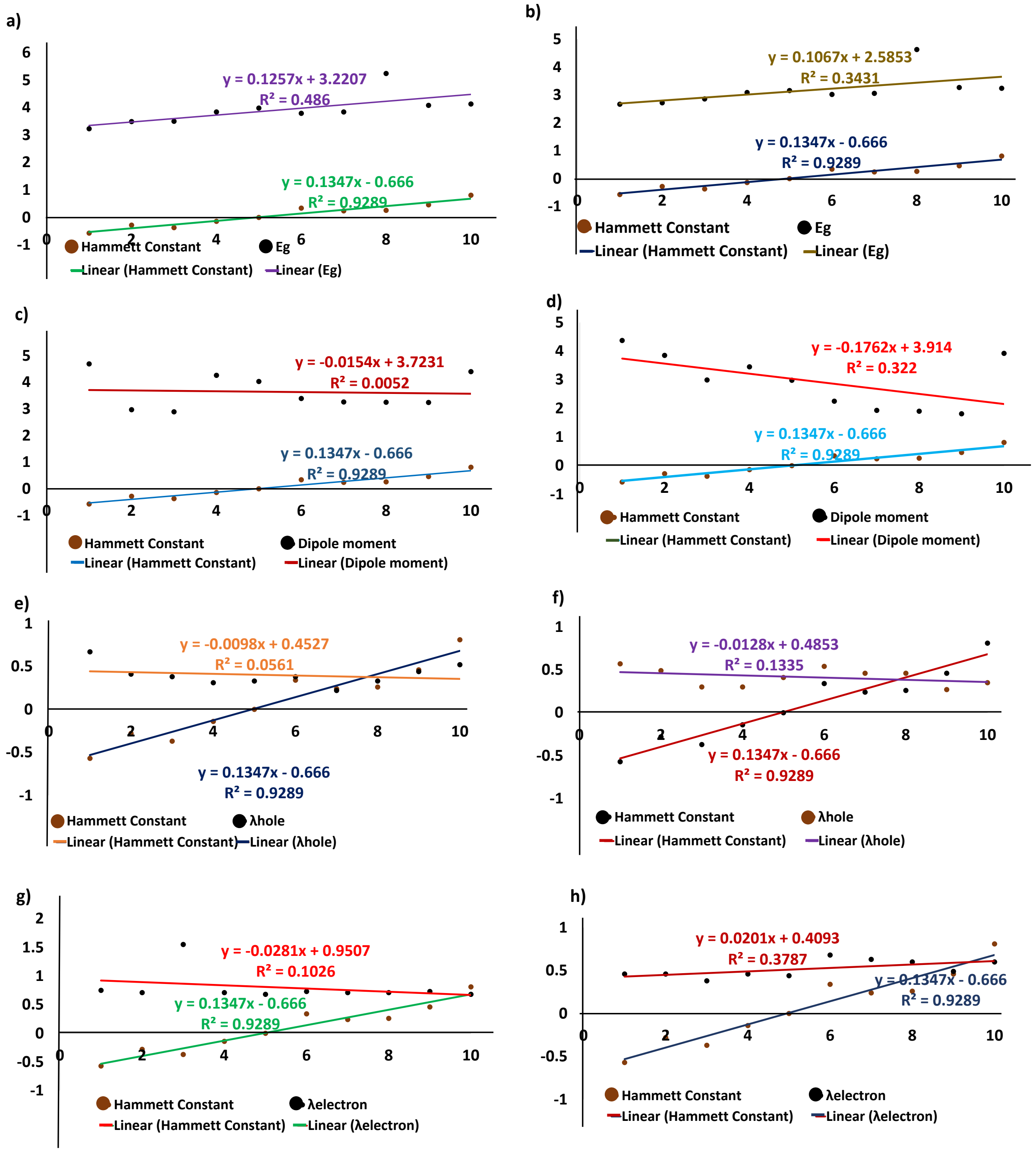

Figure S6. Correlation diagrams of a) & b) E_g of enol and keto forms; c) &d) Dipole moments of enol and keto forms e) & f) λ_{hole} of enol and keto forms; g) & h) $\lambda_{electron}$ of enol and keto forms respectively.

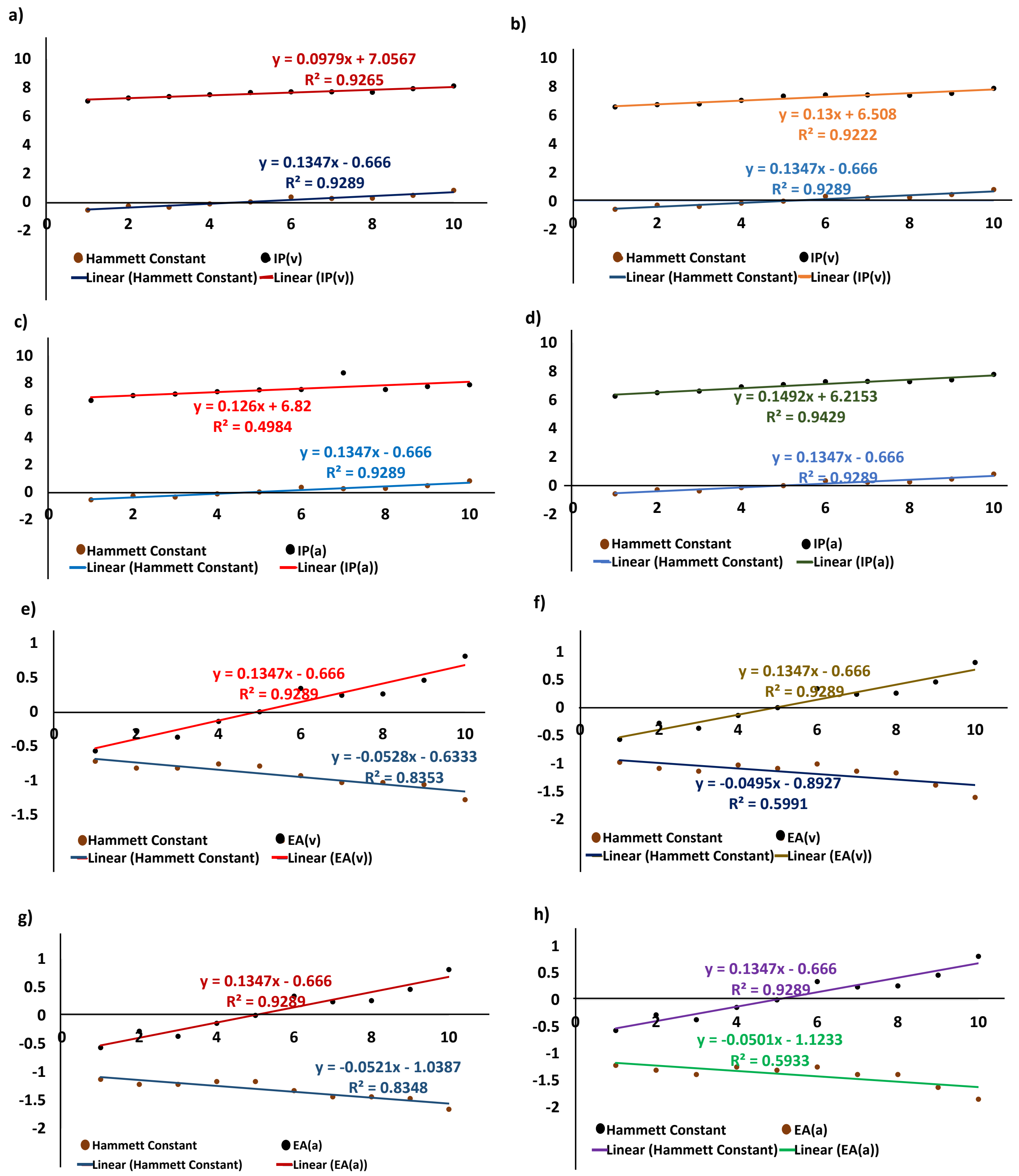

Figure S7. Correlation diagrams between hammett constant vs a) & b) IP(v) of enol and keto forms; c) &d) IP(a) of enol and keto forms; e) & f) EA(v) of enol and keto forms; g) & h) EA(a) of enol and keto forms, respectively.

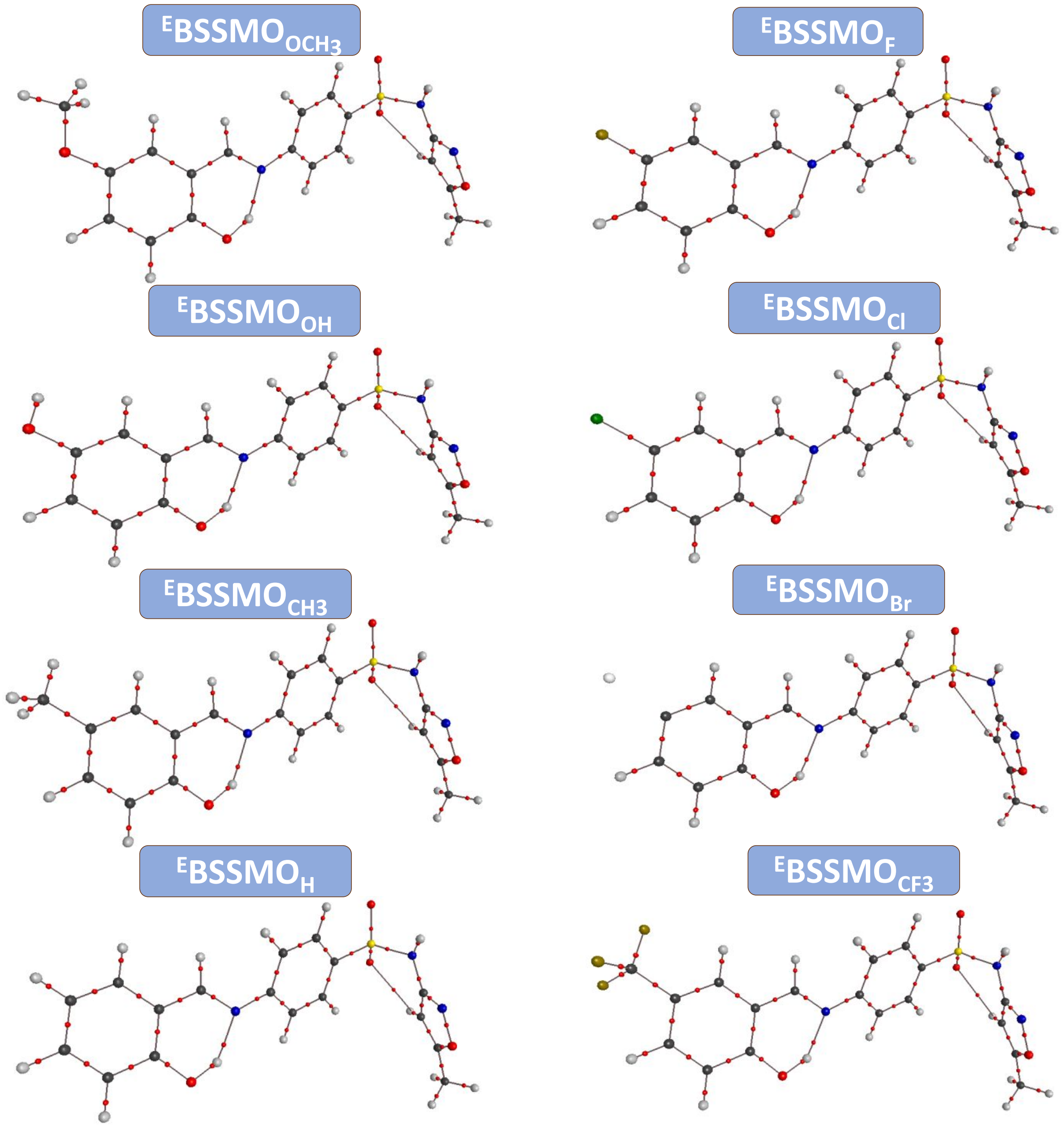

Figure S8. Molecular graphs and electron gradient image of various substituents in enol ($^{E}BSSMO_{R}$) forms at Ground (S_0) state.

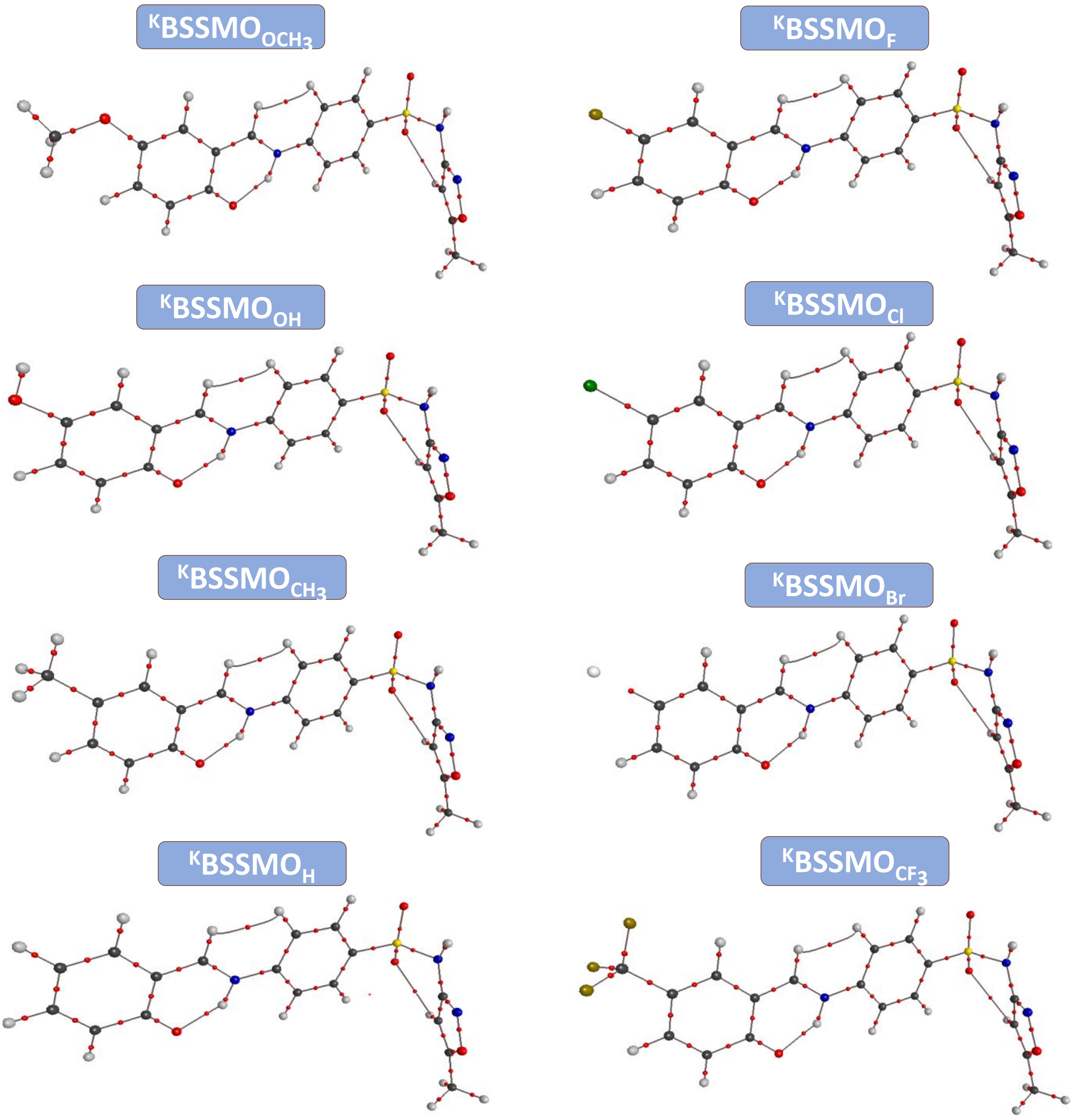

Figure S9. Molecular graphs and electron gradient image of various substituents in enol ($^{K}BSSMO_{R}$) forms at Ground (S_0) state.

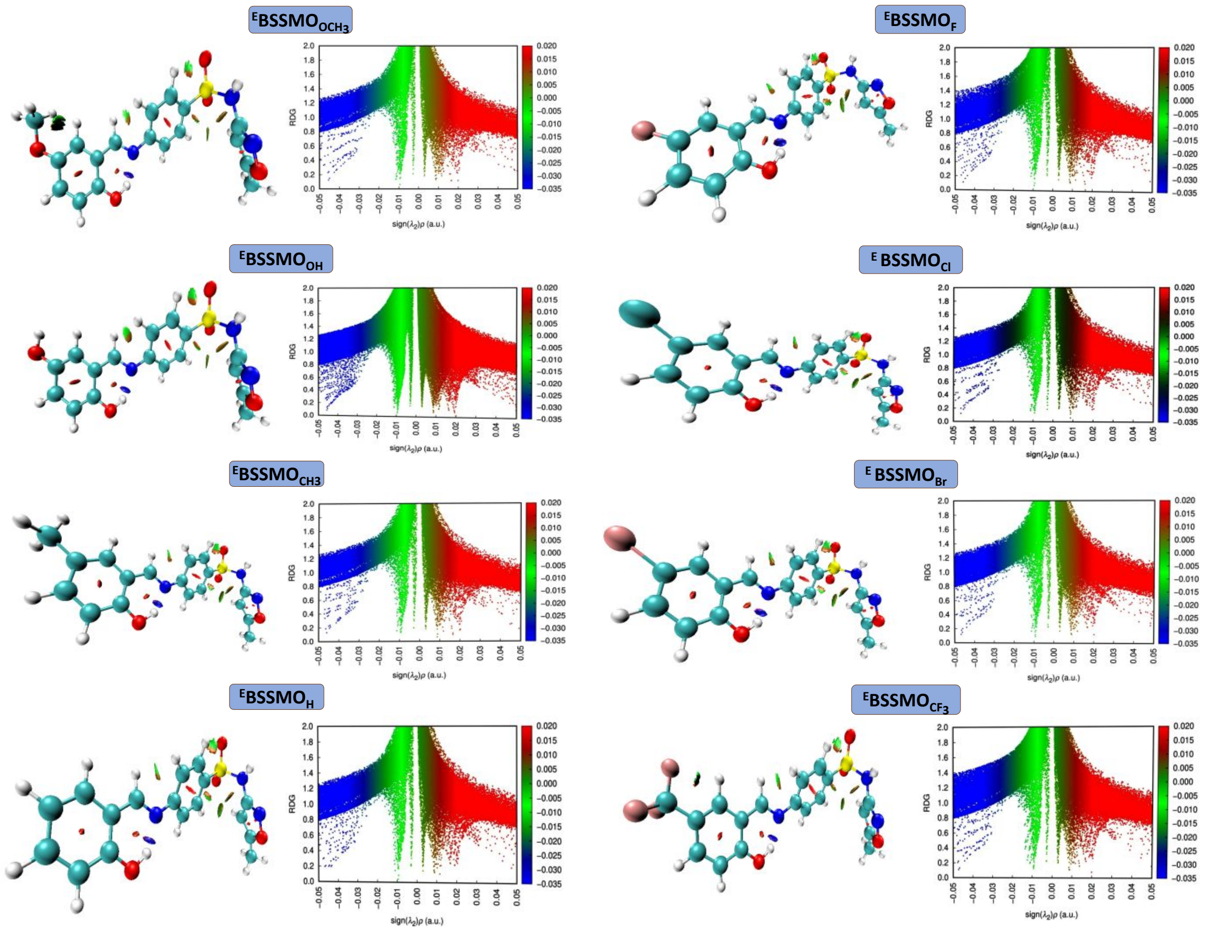

Figure S10. NCI Plots and RDG isosurfaces of enol forms for BSSMO derivatives ($^{E}BSSMO_{R}$) at S_0 State.

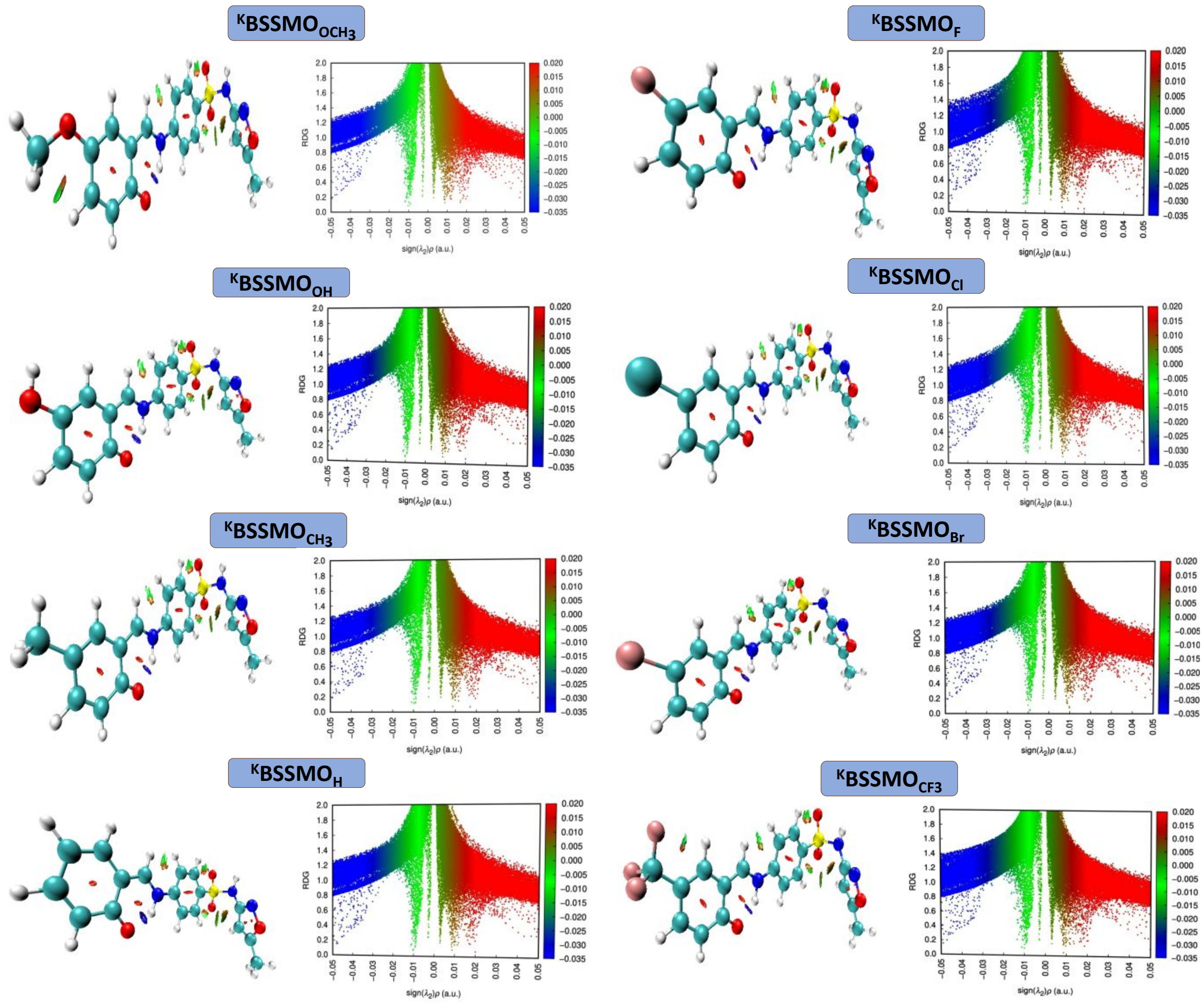

Figure S11. NCI Plots and RDG isosurfaces of keto forms for BSSMO derivatives (^{K}BSSMO$_R$) at S_0 State.

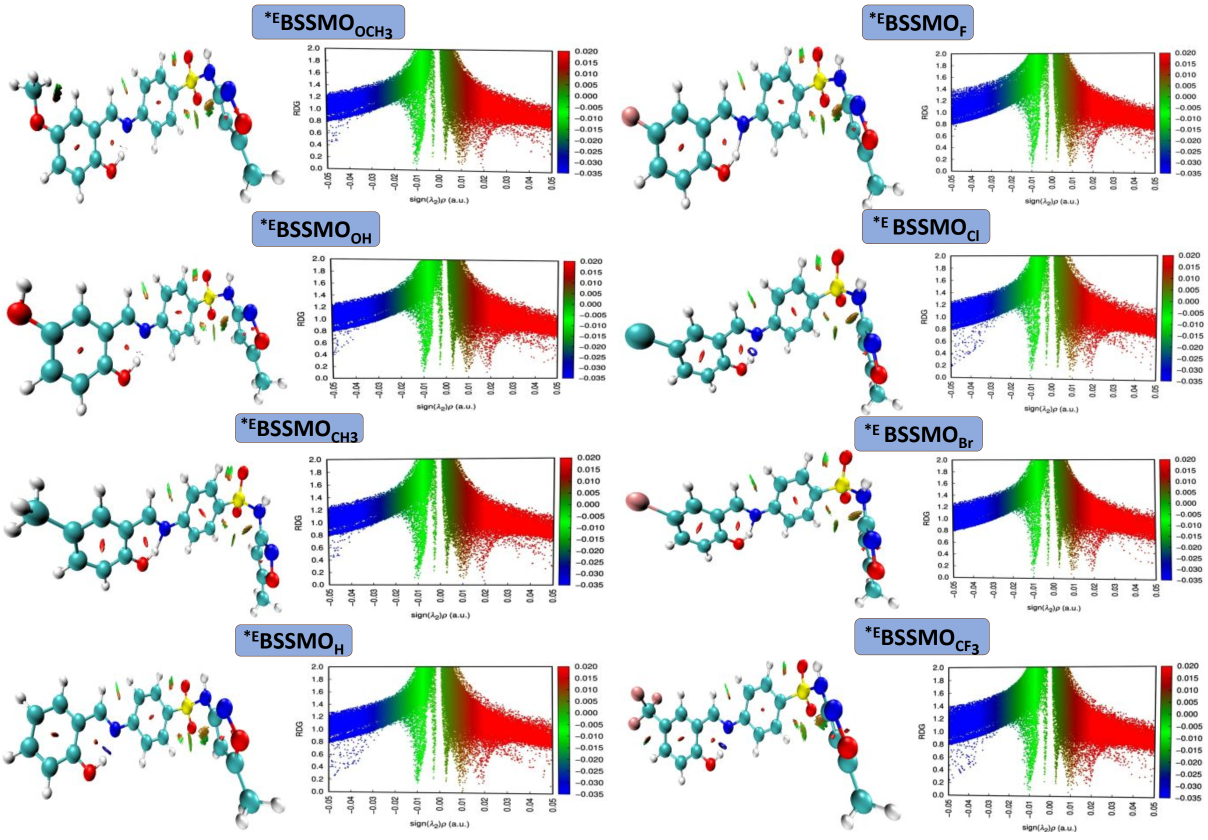

Figure S12. NCI Plots and RDG isosurfaces of keto forms for BSSMO derivatives (^{*E}BSSMO$_R$) at S_1 State.

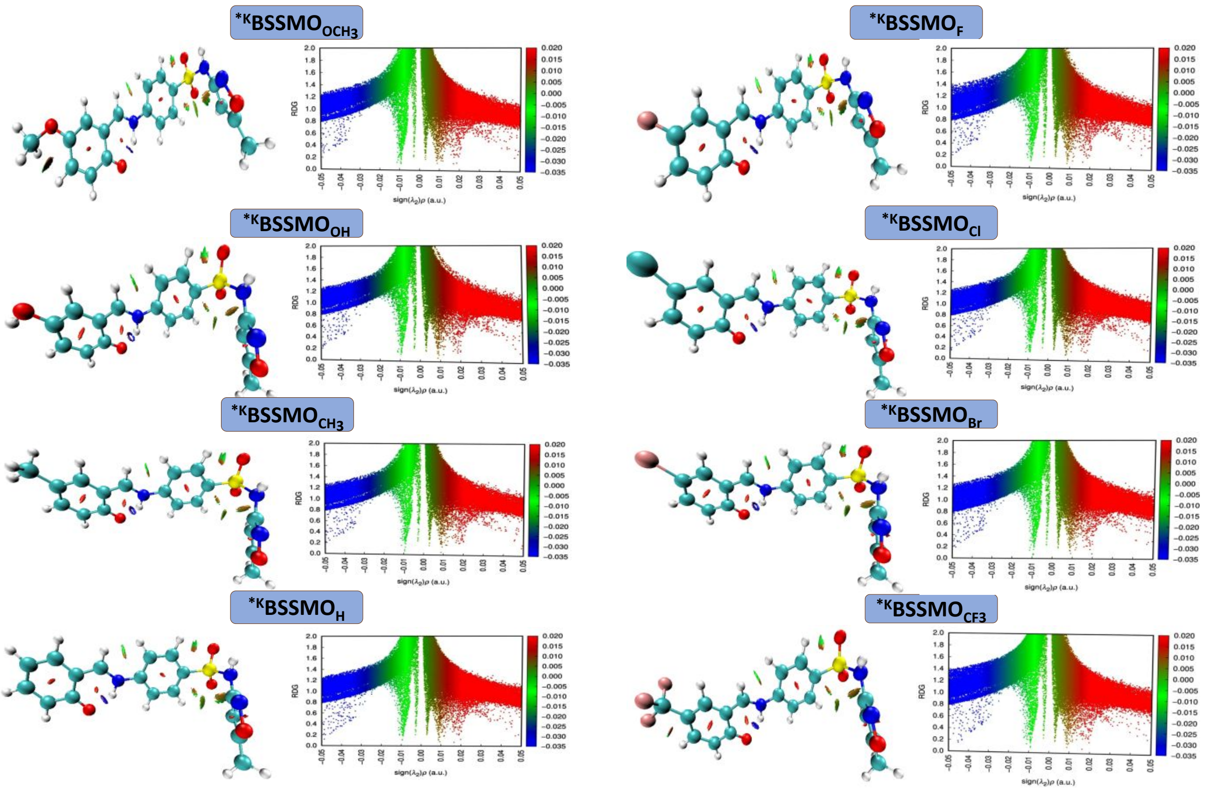

Figure S13. NCI Plots and RDG isosurfaces of keto forms for BSSMO derivatives ($^{*K}BSSMO_R$) at S_1 State.

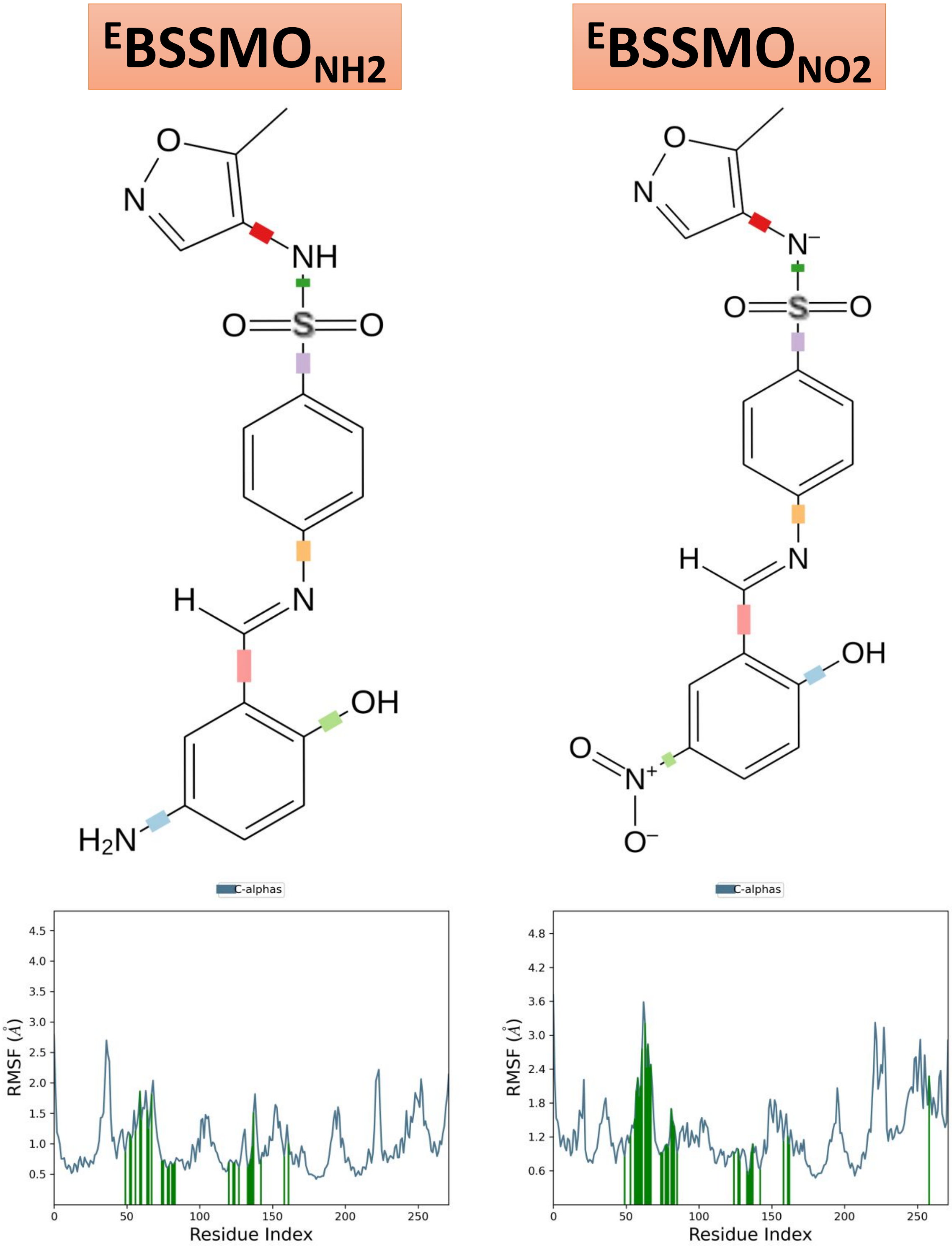

Figure S14.RMSF plots of the $^{E}BSSMO_{NH2}$ and $^{E}BSSMO_{NO2}$ with 2HAP Protein.

Table S1. Energy levels of E_g (in eV) for enol (NON-ESIPT) forms of substituted $^{NON-ESIPT}BSSMO_R$ derivatives at Ground state (S_0) in the gas phase.

Molecules	HOMO-2	HOMO-1	HOMO	LUMO	LUMO+1	LUMO+2	E_g (eV)
$^EBSSMO_{NH2}$	-6.78	-6.27	-5.48	-1.81	-0.60	-0.45	3.67
$^EBSSMO_{OCH3}$	-6.80	-6.32	-5.81	-1.87	-0.63	-0.48	3.94
$^EBSSMO_{OH}$	-6.81	-6.36	-5.87	-1.91	-0.66	-0.49	3.95
$^EBSSMO_{CH3}$	-6.81	-6.46	-6.08	-1.86	-0.63	-0.4	4.21
$^EBSSMO_H$	-6.83	-6.59	-6.19	-1.90	-0.66	-0.50	4.29
$^EBSSMO_F$	-6.89	-6.59	-6.24	-2.07	-0.78	-0.58	4.17
$^EBSSMO_{Cl}$	-6.91	-6.65	-6.32	-2.13	-0.82	-0.61	4.19
$^EBSSMO_{Br}$	-6.91	-6.61	-6.30	-2.12	-0.81	-0.61	4.17
$^EBSSMO_{CF3}$	-7.01	-6.88	-6.44	-2.19	-0.85	-0.83	4.25
$^EBSSMO_{NO2}$	-7.23	-6.99	-6.60	-2.60	-2.32	-1.00	3.99

Table S2. Energy levels of E_g (in eV) for enol forms of substituted $^EBSSMO_R$ derivatives at Ground state (S_0) in the gas phase.

Molecules	HOMO-2	HOMO-1	HOMO	LUMO	LUMO+1	LUMO+2	E_g (eV)
$^EBSSMO_{NH2}$	-6.95	-6.59	-5.38	-2.14	-0.78	-0.74	3.23
$^EBSSMO_{OCH3}$	-6.99	-6.68	-5.71	-2.22	-0.83	-0.78	3.49
$^EBSSMO_{OH}$	-7.02	-6.72	-5.77	-2.26	-0.87	-0.81	3.50
$^EBSSMO_{CH3}$	-6.98	-6.68	-6.04	-2.19	-0.83	-0.78	3.84
$^EBSSMO_H$	-7.00	-6.74	-6.21	-2.23	-0.85	-0.80	3.98
$^EBSSMO_F$	-7.09	-6.83	-6.19	-2.39	-0.97	-0.89	3.79
$^EBSSMO_{Cl}$	-7.13	-6.87	-6.29	-2.45	-1.01	-0.93	3.84
$^EBSSMO_{Br}$	-7.12	-6.86	-6.25	-1.00	-0.92	-0.44	5.24
$^EBSSMO_{CF3}$	-7.19	-6.97	-6.59	-2.50	-1.05	-0.97	4.08
$^EBSSMO_{NO2}$	-7.33	-7.11	-6.85	-2.71	-2.45	-1.19	4.13

Table S3. Energy levels of E_g (in eV) for keto forms of substituted $^KBSSMO_R$ derivatives at Ground state (S_0) in the gas phase.

Molecules	HOMO-2	HOMO-1	HOMO	LUMO	LUMO+1	LUMO+2	E_g (eV)
$^KBSSMO_{NH2}$	-6.69	-6.66	-5.05	-2.37	-0.87	-0.83	2.67
$^KBSSMO_{OCH3}$	-6.73	-6.71	-5.21	-2.49	-0.95	-0.90	2.72
$^KBSSMO_{OH}$	-6.81	-6.79	-5.36	-2.49	-0.96	-0.92	2.86
$^KBSSMO_{CH3}$	-6.76	-6.71	-5.55	-2.45	-0.97	-0.90	3.09
$^KBSSMO_H$	-6.80	-6.74	-5.68	-2.51	-1.02	-0.94	3.16
$^KBSSMO_F$	-6.94	-6.92	-5.69	-2.66	-1.08	-1.03	3.02
$^KBSSMO_{Cl}$	-7.00	-6.98	-5.80	-2.74	-1.14	-1.09	3.06
$^KBSSMO_{Br}$	-6.99	-6.97	-5.78	-1.14	-1.09	-0.46	4.63
$^KBSSMO_{CF3}$	-7.06	-7.05	-6.08	-2.81	-1.12	-1.15	3.27
$^KBSSMO_{NO2}$	-7.30	-7.19	-6.36	-3.12	-2.08	-1.39	3.24

Table S4. Energy levels of E_g (in eV) for Cu forms of substituted $^{Cu}BSSMO_R$ derivatives at Ground state (S_0) in the gas phase.

Molecules	HOMO-2	HOMO-1	HOMO	LUMO	LUMO+1	LUMO+2	E_g (eV)
$^{Cu}BSSMO_{NH2}$	-5.93	-4.86	-4.71	-1.70	-1.67	-0.79	2.16
$^{Cu}BSSMO_{OCH3}$	-5.98	-5.07	-4.92	-1.79	-1.74	-0.83	2.33
$^{Cu}BSSMO_{OH}$	-6.04	-5.16	-4.99	-1.81	-1.79	-0.88	2.33
$^{Cu}BSSMO_{CH3}$	-6.03	-5.44	-5.23	-1.73	-1.71	-0.83	2.58
$^{Cu}BSSMO_{H}$	-6.08	-5.60	-5.38	-1.78	-1.75	-0.86	2.67
$^{Cu}BSSMO_{F}$	-6.22	-5.58	-5.38	-1.96	-1.94	-0.98	2.55
$^{Cu}BSSMO_{Cl}$	-6.30	-5.70	-5.51	-2.02	-2.00	-1.03	2.59
$^{Cu}BSSMO_{Br}$	-6.31	-5.68	-5.50	-2.02	-2.00	-1.02	2.57
$^{Cu}BSSMO_{CF3}$	-6.42	-6.02	-5.80	-2.08	-2.06	-1.07	2.77
$^{Cu}BSSMO_{NO2}$	-6.66	-6.31	-6.11	-2.33	-2.27	-2.01	2.83

Table S5. Potential energy landscape energies for the enol form of $^{*E}BSSMO_R$ reactants, transition states ($^{*TS}BSSMO_R$), and products ($^{*K}BSSMO_R$) for the ESIPT mechanism of EDG/EWG-substituted derivatives in the gas phase. Energies are referenced to the enol form of the $^{*E}BSSMO_R$ reactants and reported in kcal/mol.

Substituent Derivatives (R)	Enol Form (Reactant)	ESIPT (TS)	Keto Form (Product)
**$BSSMO_{NH2}$**	0	7.28	3.47
**$BSSMO_{OCH3}$**	0	6.17	4.63
**$BSSMO_{OH}$**	0	6.75	3.30
**$BSSMO_{CH3}$**	0	6.09	3.68
**$BSSMO_{H}$**	0	5.99	3.59
**$BSSMO_{F}$**	0	5.83	3.43
**$BSSMO_{Cl}$**	0	5.80	3.19
**$BSSMO_{Br}$**	0	5.21	3.21
**$BSSMO_{CF3}$**	0	4.94	2.97
**$BSSMO_{NO2}$**	0	4.44	2.42

Table S6. Potential energy landscape energies for the enol form of $^{E}BSSMO_{R}$ reactants, transition states ($^{TS}BSSMO_{R}$), and products ($^{K}BSSMO_{R}$) for the GSIPT mechanism of EDG/EWG-substituted derivatives in the gas phase. Energies are referenced to the enol form of the $^{E}BSSMO_{R}$ reactants and reported in kcal/mol.

**Substituent Derivatives (R)**	**Enol Form (Reactant)**	**GSIPT (TS)**	**Keto Form (Product)**
**$BSSMO_{NH2}$**	0	0.65	-3.47
**$BSSMO_{OCH3}$**	0	Barrier-less	-4.63
**$BSSMO_{OH}$**	0	0.74	-3.30
**$BSSMO_{CH3}$**	0	0.46	-3.68
**$BSSMO_{H}$**	0	0.31	-3.59
**$BSSMO_{F}$**	0	0.57	-3.43
**$BSSMO_{Cl}$**	0	0.65	-3.19
**$BSSMO_{Br}$**	0	0.56	-3.21
**$BSSMO_{CF3}$**	0	0.51	-2.97
**$BSSMO_{NO2}$**	0	0.62	-2.42

Table S7. Calculated electronic properties [λ_{abs} and λ_{ems} in nm, f_0] of BSSMO derivatives at B3LYP/6- 311+G(d,p) in the DMF solvent along with the respective δ_p.

**Enol Absorption ($\lambda_{abs,}$ nm) in DMF solvent medium**										
**R**	**NH_2**	**OCH_3**	**OH**	**CH_3**	**H**	**F**	**Cl**	**Br**	**CF_3**	**NO_2**
**λ1**	450.315	410.053	403.512	371.721	355.97	367.961	367.85	370.16	342.879	356.809
**f_0**	0.1771	0.2966	0.2922	0.3969	0.5089	0.4433	0.4118	0.3805	0.633	0.2734
**λ2**	323.115	318.835	321.388	318.664	316.204	318.985	319.543	320.405	308.939	339.481
**f_0**	0.7855	0.6175	0.6217	0.5929	0.4505	0.419	0.4673	0.5105	0.2053	0.6694
**λ3**	282.732	289.325	278.054	284.3	282.513	281.106	243.157	281.135	274.999	319.71
**f_0**	0.1781	0.0931	0.1202	0.1977	0.2189	0.1889	0.2197	0.1678	0.2629	0.2075
**λ4**	262.194	278.476	245.269	216.673	216.388	214.459	214.479	245.606	210.691	267.396
**f_0**	0.1674	0.1427	0.1382	0.1756	0.1698	0.2447	0.1878	0.2249	0.1423	0.1694
**Enol Emission ($\lambda_{ems,}$ nm) in DMF solvent medium**										
**R**	**NH_2**	**OCH_3**	**OH**	**CH_3**	**H**	**F**	**Cl**	**Br**	**CF_3**	**NO_2**
**λ1**	639.217	513.81	516.62	489.378	427.494	466.397	444.905	469.707	405.793	370.289
**f_0**	0.1397	0.2467	0.2244	0.2052	0.3179	0.2755	0.31	0.2671	0.4393	0.1119
**λ2**	349.621	337.684	345.086	349.829	347.967	344.926	346.856	347.171	341.289	336.594
**f_0**	0.894	0.9766	0.9975	1.0385	0.9387	0.908	0.941	0.9585	0.7517	0.7989
**λ3**	344.78	300.502	253.644	247.63	235.768	241.056	258.141	243.961	224.364	268.431
**f_0**	0.1671	0.0697	0.1011	0.1628	0.0964	0.1088	0.0305	0.1426	0.1399	0.2186
**λ4**	237.957	213.874	235.93	237.366	231.221	234.145	233.874	223.448	209.387	264.033
**f_0**	0.0241	0.0617	0.1032	0.077	0.1404	0.0847	0.1488	0.1371	0.1848	0.3032
**Keto Emission ($\lambda_{ems,}$ nm) in DMF solvent medium**										
**R**	**NH_2**	**OCH_3**	**OH**	**CH_3**	**H**	**F**	**Cl**	**Br**	**CF_3**	**NO_2**
**λ1**	681.24	604.416	592.334	562.952	537.481	539.861	540.021	538	544.942	510.188
**f_0**	0.261	0.274	0.2769	0.2374	0.2234	0.2833	0.285	0.2952	0.2032	0.3369
**λ2**	346.787	349.09	349.082	349.593	349.709	345.832	346.675	346.751	347.51	356.844
**f_0**	0.9167	0.1476	0.3577	0.8766	0.8635	0.7269	0.7688	0.7876	0.7259	0.7308
**λ3**	284.81	339.65	355.715	263.605	252.441	313.221	315.007	314.61	244.73	366.211
**f_0**	0.0344	0.2374	0.2971	0.0824	0.0687	0.0101	0.1241	0.1217	0.1216	0.1866
**λ4**	243.81	335.388	342.865	225.438	239.724	237.492	237.108	237.712	233.135	336.26
**f_0**	0.212	0.5792	0.2778	0.0634	0.1146	0.1	0.1668	0.2187	0.0884	0.1727

Table S8. Calculated electronic properties [λ_{abs} and λ_{ems} in nm and f] of BSSMO derivatives at B3LYP/6- 311+G(d,p) in the DCM solvent along with the respective δ_p .

Enol Absorption (λ_{abs}, nm) in DCM solvent at Gas Phase										
**R**	**NH_2**	**OCH_3**	**OH**	**CH_3**	**H**	**F**	**Cl**	**Br**	**CF_3**	**NO_2**
**λ1**	450.32	410.59	404.32	372.23	356.64	368.91	368.97	371.36	343.47	353.8
**f_0**	0.179	0.297	0.293	0.396	0.504	0.438	0.406	0.374	0.627	0.328
**λ2**	323.4	319.08	321.64	318.95	316.54	319.46	320.02	320.89	309.37	307.49
**f_0**	0.775	0.613	0.614	0.587	0.449	0.418	0.468	0.511	0.207	0.044
**λ3**	282.7	289.07	278.33	284.22	282.44	279.56	279.68	282.13	275.08	276.28
**f_0**	0.181	0.097	0.082	0.201	0.22	0.177	0.203	0.055	0.268	0.107
**λ4**	262.33	278.77	245.28	236.29	216.34	214.19	214.13	217.33	211.03	228.68
**f_0**	0.145	0.101	0.129	0.041	0.181	0.182	0.146	0.068	0.143	0.067
**Enol Emission (λ_{ems}, nm) in DCM solvent at Gas Phase**										
**R**	**NH_2**	**OCH_3**	**OH**	**CH_3**	**H**	**F**	**Cl**	**Br**	**CF_3**	**NO_2**
**λ1**	638.21	515.01	518.7	491.93	428.97	469	446.95	472.79	407.03	366.09
**f_0**	0.142	0.248	0.225	0.204	0.315	0.273	0.306	0.264	0.434	0.123
**λ2**	349.9	338.24	345.71	350.48	348.59	345.83	347.68	348.15	342.36	337.05
**f_0**	0.936	0.963	0.983	1.029	0.931	0.0892	0.926	0.942	0.729	0.695
**λ3**	284.14	300.03	301.16	306.29	305.77	311.07	310.9	313.1	296.76	265.16
**f_0**	0.062	0.023	0.024	0.034	0.041	0.061	0.063	0.067	0.044	0.391
**λ4**	264.2	286.53	289.99	248.13	217.54	217.75	218.64	221.29	214.16	241.52
**f_0**	0.0006	0.026	0.033	0.161	0.049	0.002	0.028	0.008	0.038	0.043
**Keto Emission (λ_{ems}, nm) in DCM solvent at Gas Phase**										
**R**	**NH_2**	**OCH_3**	**OH**	**CH_3**	**H**	**F**	**Cl**	**Br**	**CF_3**	**NO_2**
**λ1**	679.57	606.64	595.14	567.11	542.53	543.77	544.59	542.53	550.28	516.34
**f_0**	0.265	0.275	0.277	0.237	0.221	0.282	0.284	0.294	0.201	0.324
**λ2**	382.19	349.97	350.06	350.36	350.61	346.59	347.62	347.66	348.68	358.34
**f_0**	0.02	0.13	0.301	0.861	0.854	0.711	0.753	0.771	0.711	0.731
**λ3**	346.77	305.36	307.76	309.02	311.97	314.61	317.02	311.46	303.6	285.57
**f_0**	0.909	0.048	0.038	0.031	0.03	0.113	0.095	0.072	0.027	0.09
**λ4**	283.74	289.68	273.61	264.26	238.61	238.3	237.67	238.3	234.52	243.87
**f_0**	0.033	0.015	0.039	0.079	0.022	0.105	0.194	0.238	0.091	0.012

Table S9. Calculated electronic properties [λ_{abs} and λ_{ems} in nm and f] of BSSMO derivatives at B3LYP/6- 311+G(d,p) in the TOLUENE solvent along with the respective δ_p.

**Enol Absorption ($\lambda_{abs,}$ nm) in Toluene solvent at Gas Phase**										
**R**	**NH_2**	**OCH_3**	**OH**	**CH_3**	**H**	**F**	**Cl**	**Br**	**CF_3**	**NO_2**
**λ1**	451.53	413.35	407.95	374.89	359.83	373.16	373.69	376.32	346.51	348.64
**f_0**	0.192	0.311	0.308	0.409	0.509	0.441	0.404	0.371	0.63	0.728
**λ2**	325.13	320.52	323.37	320.55	318.3	321.61	322.22	323.14	311.43	307.79
**f_0**	0.762	0.61	0.604	0.576	0.448	0.422	0.476	0.52	0.21	0.011
**λ3**	282.75	288.58	277.69	284.24	282.56	280.57	280.5	280.64	275.43	278.41
**f_0**	0.181	0.113	0.15	0.211	0.217	0.254	0.248	0.254	0.262	0.025
**λ4**	261.57	277.9	245.09	235.36	216.11	213.34	214.84	216.92	211.69	229.45
**f_0**	0.108	0.179	0.085	0.106	0.216	0.051	0.141	0.014	0.057	0.056
**Enol Emission ($\lambda_{ems,}$ nm) in Toluene solvent at Gas Phase**										
**R**	**NH_2**	**OCH_3**	**OH**	**CH_3**	**H**	**F**	**Cl**	**Br**	**CF_3**	**NO_2**
**λ1**	637.36	520.35	526.86	501.48	435.04	479.04	455.12	484.45	412.49	362.65
**f_0**	0.156	0.261	0.239	0.212	0.321	0.277	0.31	0.266	0.439	0.0002
**λ2**	352.23	341.26	349.19	354.2	352.11	350.29	352.11	352.87	347.65	340.14
**f_0**	1.013	0.925	0.942	1.007	0.903	0.844	0.873	0.89	0.637	0.627
**λ3**	283.69	301.42	301.08	313.52	313.45	318.74	319.04	321.01	302.97	268.9
**f_0**	0.044	0.016	0.029	0.053	0.066	0.103	0.111	0.115	0.064	0.3
**λ4**	264.69	291.01	284.07	248.74	217.72	219.25	222.39	225.38	217.33	242.18
**f_0**	0.0005	0.026	0.02	0.151	0.062	0.042	0.024	0.037	0.002	0.047
**Keto Emission ($\lambda_{ems,}$ nm) in Toluene solvent at Gas Phase**										
**R**	**NH_2**	**OCH_3**	**OH**	**CH_3**	**H**	**F**	**Cl**	**Br**	**CF_3**	**NO_2**
**λ1**	678.12	616.47	606.92	582.06	560.3	558.36	561.28	559.09	569.16	537.92
**f_0**	0.286	0.287	0.289	0.247	0.224	0.289	0.291	0.302	0.199	0.302
**λ2**	384.39	348.13	339.62	353.9	354.5	350.28	351.62	351.68	353.45	360.22
**f_0**	0.014	0.158	0.433	0.831	0.836	0.671	0.713	0.728	0.674	0.385
**λ3**	348.23	309.52	312.86	314.09	317.09	319.6	318.22	317.18	307.05	285.63
**f_0**	0.898	0.068	0.058	0.041	0.041	0.183	0.143	0.142	0.045	0.012
**λ4**	285.36	289.01	290.09	266.6	240.13	240.47	238.53	240.93	236.55	245.78
**f_0**	0.029	0.028	0.015	0.071	0.029	0.072	0.002	0.182	0.108	0.025

Table S10. Calculated electronic properties [λ_{abs} and λ_{ems} in nm and f] of BSSMO derivatives at B3LYP/6- 311+G(d,p) in the CYCLOHEXANE solvent along with the respective Hammett sigma para constants (δ_p).

**Enol Absorption ($\lambda_{abs,}$ nm) in cyclohexane solvent at Gas Phase**										
**R**	**NH_2**	**OCH_3**	**OH**	**CH_3**	**H**	**F**	**Cl**	**Br**	**CF_3**	**NO_2**
**λ1**	450.94	412.99	407.8	374.63	359.68	373.22	373.96	376.68	346.35	347.91
**f_0**	0.189	0.305	0.301	0.397	0.493	0.427	0.39	0.358	0.614	0.748
**λ2**	324.84	320.349	323.2	320.4	318.27	321.69	322.31	323.21	311.7	306.75
**f_0**	0.741	0.597	0.589	0.565	0.442	0.414	0.469	0.512	0.206	0.046
**λ3**	282.56	288.36	277.5	284.04	282.45	280.41	280.37	280.5	275.29	278.24
**f_0**	0.175	0.115	0.15	0.215	0.151	0.247	0.241	0.247	0.257	0.013
**λ4**	260.76	277.71	244.89	235.19	216.14	216.19	223.14	226.26	214.57	229.67
**f_0**	0.073	0.179	0.073	0.095	0.198	0.178	0.024	0.018	0.007	0.047
**Enol Emission ($\lambda_{ems,}$ nm) in Cyclohexane solvent at Gas Phase**										
**R**	**NH_2**	**OCH_3**	**OH**	**CH_3**	**H**	**F**	**Cl**	**Br**	**CF_3**	**NO_2**
**λ1**	635.48	519.99	527.1	502.39	435.37	479.97	455.86	485.85	412.68	352.3
**f_0**	0.153	0.256	0.234	0.206	0.309	0.269	0.299	0.258	0.422	0.268
**λ2**	351.69	341.04	348.96	353.89	351.87	350.4	352.22	353.03	348.29	339.98
**f_0**	0.985	0.899	0.915	0.986	0.885	0.813	0.843	0.857	0.6	0.562
**λ3**	283.49	301.82	300.91	315	315	320.22	320.6	322.53	304.23	269.29
**f_0**	0.027	0.015	0.029	0.06	0.076	0.12	0.129	0.133	0.073	0.295
**λ4**	265.28	291.91	284.07	248.7	217.69	219.4	223.14	226.26	217.73	242.16
**f_0**	0.0005	0.026	0.019	0.145	0.064	0.04	0.024	0.018	0.106	0.045
**Keto Emission ($\lambda_{ems,}$ nm) in Cyclohexane solvent at Gas Phase**										
**R**	**NH_2**	**OCH_3**	**OH**	**CH_3**	**H**	**F**	**Cl**	**Br**	**CF_3**	**NO_2**
**λ1**	674.01	615.55	606.33	582.72	561.95	558.9	562.4	560.22	571.43	540.77
**f_0**	0.282	0.282	0.284	0.242	0.218	0.282	0.284	0.295	0.192	0.291
**λ2**	384.85	337.86	339.57	353.72	354.32	350.36	351.74	351.81	353.72	365.99
**f_0**	0.882	0.714	0.458	0.801	0.815	0.643	0.685	0.698	0.646	0.431
**λ3**	347.55	310.35	313.9	315.12	318.12	320.31	319.06	318.02	308.39	283.06
**f_0**	0.882	0.076	0.066	0.046	0.046	0.205	0.161	0.158	0.059	0.014
**λ4**	285.53	288.85	272.64	266.95	238.54	241.22	240.99	240.89	235.86	246.08
**f_0**	0.025	0.027	0.0004	0.067	0.0007	0.086	0.123	0.039	0.001	0.027

Table S11. Topological parameters obtained from QTAIM analysis at the bond critical points (BCPs) for the enol forms of BSSMO derivatives. *ρ(r)*: electron density; *G(r)*: kinetic energy density; *V(r)*: potential energy density; *L(r)*: total energy density (L(r) = G(r) + V(r)); λ_1 and λ_2: negative eigenvalues of the Hessian matrix of ρ(r), representing the curvature perpendicular to the bond path.

**Molecules**	**Bonds**	**Bond distance**	***ρ (r)***	***L (r)***	***G (r)***	***V (r)***	**λ1**	**λ2**
$^{E}BSSMO_{NH2}$	O-H	0.993	0.308784	0.389916	0.452394	0.514873	-1.550003	-1.530664
	N-H	1.776	0.04905	-0.032601	0.003865	0.040333	-0.079686	-0.07817
$^{E}BSSMO_{OCH3}$	O-H	0.995	0.314779	0.402372	0.062698	0.527769	-1.584131	-1.562447
	N-H	1.766	0.046211	-0.03148	0.034741	0.038001	-0.072967	-0.071529
$^{E}BSSMO_{OH}$	O-H	0.994	0.314259	0.401401	0.062523	0.526448	-1.581042	-1.559699
	N-H	1.771	0.046161	-0.031358	0.034625	0.037892	-0.072894	-0.071439
$^{E}BSSMO_{CH3}$	O-H	0.995	0.315546	0.404218	0.062588	0.529395	-1.589131	-1.567218
	N-H	1.766	0.045634	-0.031189	0.034334	0.03748	-0.071681	-0.070297
$^{E}BSSMO_{H}$	O-H	0.996	0.313991	0.400842	0.062539	0.525922	-1.579729	-1.558545
	N-H	1.764	0.046397	-0.031494	0.034804	0.038115	-0.073466	-0.072003
$^{E}BSSMO_{F}$	O-H	0.995	0.314369	0.401831	0.062574	0.526979	-1.582869	-1.561327
	N-H	1.765	0.046297	-0.031494	0.034767	0.038041	-0.073234	-0.071829
$^{E}BSSMO_{Cl}$	O-H	0.996	0.313155	0.399336	0.062489	0.524315	-1.575913	-1.55489
	N-H	1.766	0.046805	-0.031681	0.035062	0.038443	-0.074405	-0.072979
$^{E}BSSMO_{Br}$	O-H	0.996	0.313034	0.399038	0.062503	0.524045	-1.575085	-1.554117
	N-H	1.760	0.046899	-0.031728	0.035128	0.038529	-0.074622	-0.073187
$^{E}BSSMO_{CF3}$	O-H	0.998	0.311364	0.395462	0.062467	0.520396	-1.565153	-1.544878
	N-H	1.752	0.047711	-0.032063	0.035636	0.03921	-0.076506	-0.0750325
$^{E}BSSMO_{NO2}$	O-H	1.000	0.308784	0.389916	0.062478	0.514873	-1.550003	-1.530664
	N-H	1.740	0.04905	-0.032601	0.036467	0.040333	-0.079686	-0.078177

Table S12. Topological parameters obtained from QTAIM analysis at the bond critical points (BCPs) for the Keto forms of BSSMO derivatives. *ρ(r)*: electron density; *G(r)*: kinetic energy density; *V(r)*: potential energy density; *L(r)*: total energy density (L(r) = G(r) + V(r)); λ_1 and λ_2: negative eigenvalues of the Hessian matrix of ρ(r), representing the curvature perpendicular to the bond path.

Molecules	Bonds	Bond distance	*ρ (r)*	*L (r)*	*G (r)*	*V (r)*	λ1	λ2
$^{K}BSSMO_{NH2}$	O-H	1.71	0.049097	-0.038828	0.040908	0.042989	-0.079515	-0.079422
	N-H	1.04	0.303061	0.372766	0.041953	0.456674	-1.226601	-1.194273
$^{K}BSSMO_{OCH3}$	O-H	1.692	0.051182	-0.040162	0.042525	0.044888	-0.084587	-0.084395
	N-H	1.044	0.299837	0.366774	0.042251	0.451276	-1.212271	-1.181155
$^{K}BSSMO_{OH}$	O-H	1.709	0.050199	-0.039359	0.041617	0.043874	-0.082202	-0.082078
	N-H	1.041	0.300657	0.368445	0.042021	0.452488	-1.215861	-1.184391
$^{K}BSSMO_{CH3}$	O-H	1.701	0.049263	-0.038967	0.041051	0.043135	-0.079915	-0.079851
	N-H	1.043	0.302756	0.372301	0.041916	0.456134	-1.225802	-1.193666
$^{K}BSSMO_{H}$	O-H	1.692	0.05117	-0.040015	0.042394	0.044773	-0.084593	-0.084414
	N-H	1.044	0.299243	0.365811	0.042167	0.450145	-1.209657	-1.178703
$^{K}BSSMO_{F}$	O-H	1.699	0.050276	-0.039609	0.041821	0.044033	-0.082396	-0.082309
	N-H	1.043	0.30098	0.369165	0.041953	0.453073	-1.218299	-1.187044
$^{K}BSSMO_{Cl}$	O-H	1.699	0.050313	-0.039529	0.041762	0.043995	-0.082535	-0.082442
	N-H	1.043	0.30048	0.368393	0.041862	0.452117	-1.216203	-1.185291
$^{K}BSSMO_{Br}$	O-H	1.7	0.050224	-0.039435	0.041664	0.043893	-0.082323	-0.082224
	N-H	1.043	0.300479	0.368423	0.04183	0.452083	-1.216231	-1.185332
$^{K}BSSMO_{CF3}$	O-H	1.699	0.050299	-0.03939	0.041641	0.043891	-0.082534	-0.082468
	N-H	1.044	0.299913	0.367534	0.041737	0.451009	-1.213807	-1.183299
$^{K}BSSMO_{NO2}$	O-H	1.697	0.050524	-0.03951	0.041774	0.044038	-0.083215	-0.083153
	N-H	1.045	0.299202	0.366576	0.041538	0.449653	-1.211399	-1.181814

Table S13. Calculated electronic properties [λ_{abs} and λ_{ems} in nm and f] of Cu(II) Complexes at B3LYP/6- 311+G(d,p) in the gas phase along with the respective δ_p.

**Enol Absorption ($\lambda_{abs,}$ nm) of the Cu(II) Complex at Gas Phase**										
**R**	**NH_2**	**OCH_3**	**OH**	**CH_3**	**H**	**F**	**Cl**	**Br**	**CF_3**	**NO_2**
**λ1**	778.61	708.92	695.9	629.69	594.02	614.71	604.6	605.68	551.59	543.14
**f_0**	0.004	0.006	0.0034	0.0023	0.002	0.002	0.0022	0.002	0.002	0.0001
**λ2**	652.65	613.22	596	529.72	500.4	537.2	520.56	518.98	473.91	446.81
**f_0**	0.0001	0.0001	0.0002	0.0002	0.0003	0.0002	0.0002	0.0002	0.0003	0.0006
**λ3**	482.42	462.78	448.85	419.56	410.71	417.27	412.49	413.21	400.44	398.04
**f_0**	0.099	0.012	0.1236	0.1435	0.089	0.155	0.1444	0.141	0.044	0.052
**λ4**	447.62	427.76	437.42	403.3	394.01	402.17	396.56	392.88	387.7	377.48
**f_0**	0.223	0.017	0.0031	0.0053	0.099	0.0021	0.0039	0.021	0.0165	0.0117

Table S14. Comparison of emission maxima (λ_{emi}, nm) and oscillator strengths (f_0) of BSSMO derivatives in keto and enol forms, calculated in the gas phase, along with their corresponding docking score.

**Substituents (R)**	**λ_{emi} (keto) (nm)**	***f_0***	**λ_{emi} (enol) (nm)**	***f_0***	**Docking Score (Keto) (kcal/mol)**	**Docking Score (Enol) (kcal/mol)**
NH_2	676.789	0.1688	637.197	0.1333	-7.20	-4.94
OCH_3	613.020	0.2839	519.880	0.2355	-7.36	-4.43
OH	588.204	0.2790	514.490	0.2141	-6.71	-4.26
CH_3	559.442	0.3777	487.538	0.1952	-7.06	-3.65
H	534.221	0.4850	425.974	0.2997	-7.20	-5.41
F	536.231	0.4245	464.376	0.2628	-6.97	-5.29
Cl	536.460	0.3939	443.185	0.2947	-6.83	-4.79
Br	534.490	0.3636	467.736	0.2549	-6.96	-4.05
CF_3	541.961	0.6113	404.243	0.4165	-5.12	-5.30
NO_2	512.438	0.2419	375.709	0.0990	-3.35	-4.93

The spectral calculations provide insights into the electronic transitions and substituent effects on excited-state stabilization, while docking reflects the interaction strength of these derivatives with the biological target. A clear correlation emerges: electron-donating substituents (NH_2, OCH_3, OH) red-shift both absorption and emission, indicating enhanced charge transfer and conjugation, which also supports stronger hydrogen bonding and polar interactions in the docking studies (keto form, docking scores ranges from −7.36 to −6.71 kcal/mol). Conversely, strong electron-withdrawing groups (CF_3, NO_2) induce blue-shifted, weaker emissions with reduced oscillator strengths, consistent with their tendency toward nonradiative decay; this parallels their less favorable docking affinities in the keto form (−5.12 to −3.35 kcal/mol). Importantly, the keto tautomer is generally more stabilized electronically, which explains its consistently stronger docking scores compared to the enol tautomer. Thus, the gas-phase spectral calculations not only rationalize the photophysical trends but also correlate with the molecular recognition ability, suggesting that these derivatives are better candidates for dual optoelectronic and biological applications.

Table S15. Acronyms and their Expanded Forms.

Acronyms	Expanded form
DFT	Density Functional Theory
TD-DFT	Time-dependent Density Functional Theory
ESIPT	Excited State Intramolecular Proton Transfer
GSIPT	Ground-state Intramolecular Proton Transfer
FMO	Frontier Molecular Orbitals
QTAIM	Quantum Theory of Atoms in Molecules
NCI	Non-covalent Interactions
ADMET	Absorption, Distribution, Metabolism, Excretion, Toxicity

References

1. Nolte, R. T.; Wisely, G. B.; Westin, S.; Cobb, J. E.; Lambert, M. H.; Kurokawa, R.; Rosenfeld, M. G.; Willson, T. M.; Glass, C. K.; Milburn, M. V. Ligand Binding and Co-Activator Assembly of the Peroxisome Proliferator-Activated Receptor-γ. *Nature* **1998**, *395 (6698),* 137–143.
2. Lu, C.; Wu, C.; Ghoreishi, D.; Chen, W.; Wang, L.; Damm, W.; Ross, G. A.; Dahlgren, M. K.; Russell, E.; Von Bargen, C. D.; Abel, R.; Friesner, R. A.; Harder, E. D. OPLS4: Improving Force Field Accuracy on Challenging Regimes of Chemical Space. *J. Chem. Theory Comput.* **2021**, *17 (7),* 4291–4300.
3. Malgija, M. B.; Rajendran, H. A.; Darvin, S. S.; Nachimuthu, S.; Priyakumari, J. In Silico Exploration of HIV Entry Co-Receptor Antagonists: A Combination of Molecular Modeling, Docking and Molecular Dynamics Simulations. *Acta Sci. Pharm. Sci.* **2019**, *3*, 60–67.
4. Samuel, J. G.; Malgija, B.; Ebenezer, C.; Solomon, R. V. Insight into Designing of 2-Pyridone Derivatives for COVID-19 Drug Discovery—A Computational Study. *Struct. Chem.* **2023**, *34 (4),* 1289–1308.

5. Schrödinger Release 2021-4: LigPrep, Schrödinger, LLC, New York, NY, **2021**.
6. Lipinski, C. A.; Lombardo, F.; Dominy, B. W.; Feeney, P. J. Experimental and Computational Approaches to Estimate Solubility and Permeability in Drug Discovery and Development Settings. *Adv. Drug Deliv. Rev.* **1997**, *23 (1–3*), 3–25.
7. Mahmoud, W. H.; Deghadi, R. G.; Mohamed, G. G. Preparation, Geometric Structure, Molecular Docking Thermal and Spectroscopic Characterization of Novel Schiff Base Ligand and Its Metal Chelates: Screening Their Anticancer and Antimicrobial Activities. *J. Therm. Anal. Calorim.* **2017**, *127*, 2149–2171.
8. El-Gammal, O. A.; El-Bindary, A. A.; Mohamed, F. S.; Rezk, G. N.; El-Bindary, M. A. Synthesis, Characterization, Design, Molecular Docking, Anti COVID-19 Activity, DFT Calculations of Novel Schiff Base with Some Transition Metal Complexes. *J. Mol. Liq.* **2022**, *346*, 117850.
